# Patching a leak in an R1 university gateway STEM course

**DOI:** 10.1371/journal.pone.0202041

**Published:** 2018-09-06

**Authors:** Stephen Lee, Brian R. Crane, Thomas Ruttledge, Dominique Guelce, Estella F. Yee, Michael Lenetsky, Matthew Caffrey, Walter De Ath Johnsen, Anthony Lin, Shuting Lu, Marc-Anthony Rodriguez, Aboubacar Wague, Kane Wu

**Affiliations:** Department of Chemistry and Chemical Biology, Cornell University, Ithaca, NY 14853-1301, United States of America; Seattle University, UNITED STATES

## Abstract

A cognitively intensive companion service course has been introduced to the main fall general chemistry class at Cornell University. For years 2015 and 2016, priority students (those from groups under-represented and economically disadvantaged) show respectively improvement of +0.67 and +0.51 standard deviations in final course grade compared to priority students not in the program. Non-priority students show respectively a +0.66 and +0.62 standard deviation improvement. Progressive improvement (as measured by higher than expected Final Exam scores than what would have been expected solely from a given student’s earlier Exam 1 score) demonstrates conclusively the service course’s role in the enhanced outcomes. Progressive retention (as measured by the following year fall semester’s organic chemistry exam scores compared to what would have been expected based on a given student’s general chemistry final exam score) demonstrates that, on the average, the earlier observed progressive improvement is significantly retained in a chemistry course one year later. Preliminary retention statistics suggest a significant increase in first year to second year retention. A meta analysis of results from previously reported chemistry service courses indicate that such performance gains are difficult to achieve and hence common elements of the few effective programs may be of high value to the STEM education community.

## Introduction

Scientific achievement in introductory STEM gateway courses is a national concern [1, 2]. Recent educational advances, broadly centered on student-learning through flipped classes, student-led peer workshops, process-oriented guided-inquiry learning (POGIL), and other innovative teaching strategies have had a noted effect in improving student learning outcomes [3–10]. But college educators still struggle with the leaky educational pipeline: the attrition in student numbers caused by limitations of pedagogical scope in introductory STEM classes [11, 12].

Nowhere is this concern greater than for college students from under-represented minority and economically-disadvantaged backgrounds [13, 14]. We can be sharp in our framing of this concern. Starting forty years ago, Uri Treisman and others at the University of California, Berkeley, University of Texas at Austin, University of Wisconsin-Madison, Univ. of Illinois at Urbana-Champaign, and other leading schools demonstrated that through an intensive mathematics service program (Emerging Scholars Program), under-represented minority students could achieve grade distributions equal to and in some cases superior to that of the class as a whole [15–20].

Given Dr. Treisman’s success, the well-known nature of his work among college educators, and the broad advances in STEM education since the start of his work, how is it that similar achievements have not been found across the STEM fields? For under-represented minority and economically disadvantaged students, why do problems of the type that Dr. Treisman noted forty years ago (i.e. an inability of certain student groups to benefit from traditional lecture-style courses) remain so difficult to resolve even now in introductory chemistry? Here we signal introductory chemistry, because general chemistry (gen-chem) is taken early in the chemistry course progression and is considered by many undergraduates as the first great hurdle to enter the STEM fields [21, 22].

In this paper, we question whether the original insights from Dr. Treisman have been diluted in the diaspora [8, 23] of his methods to the chemical sciences. The Treisman paradigm requires a large time commitment on part of students and educators alike. The key sociological observation that Dr. Treisman noted was a six hour-a-week difference in collaborative work time between more vs. less STEM-successful demographic groups. It was this deficit which caused Dr. Treisman to initiate four hours a week of peer-led mathematics workshops for each participating student.

Dr. Treisman’s requirements for these workshops are exacting. He noted, “Most visitors to [our Emerging Scholars] program thought the heart of the project was group learning. They were impressed by the enthusiasm of the students and the intensity of their interactions as they collectively attacked challenging problems. But the real core was the problem sets which drove the group interaction” [16]. In Dr. Treisman’s eyes these workshops were based on problems which provided: (1) “the skills to earn a final grade of A in first-semester calculus, (2) a foundation of mathematics that will enable … graduates to continue to excel in … upper-division mathematics [without the assistance of the program], and (3) … [the] identif[ication] of areas in mathematical knowledge that students must strengthen to survive” [15].

Over the last two years, we have put into place an intensive service course based on the Treisman model as a companion to our first semester gen-chem course. Course content follows the stringent content model which Dr. Treisman described above. Our current realization of this Treisman methodology incorporates more recent pedagogical advances: students work through a combination of flipped classes (two hours/week), peer-assisted workshops (two hours/week), and scored practice exams (one-and-a-half hours/week). (The specific mix of course activities was chosen on an trial-and-error basis. While the workshops and flipped class appear of have the greatest pedagogical value, the feedback from the scored practice exams provide a strong motivation for students to join and continue in the program. Moreover, the scored exams provide rapid assessment of lesson effectiveness to the instructors.) To complete the course, students are required to complete 80% of the given tasks. Course materials corresponding to the first six weeks of the service course are presented in the supplementary material. A direct comparison between the service course material and current university level gen-chem textbooks are also presented in the supplementary material, highlighting cognitive differences between the service course worksheets and current textbook material and problems. A workbook based on the service course workshops designed for a full two-semester gen-chem sequence is currently under preparation.

We report on this Treiman-based service course in the context of other interventions. For context, we draw upon results connected to the Cornell gen-chem summer bridge program for pre-freshmen as well as the Cornell-Howard Hughes Accelerated Medical Scholars Program summer organic chemistry course for selected rising sophomores. In the supplementary material, we further analyze literature reported R1 university gen-chem interventions, to assess the degree to which Treisman-like programs have hitherto been adopted and to provide a uniform analysis of the efficacy of these interventions with an eye toward the import of R1-university based flipped classes and peer-assisted learning.

## Materials and methods

This work was reviewed by Cornell’s Institutional Review Board (protocol 17100707497) and granted an exemption on October 10, 2017 based on use of aggregate data. Course data were taken from chemistry department course records and were analyzed through Microsoft Excel.

## Results

### Overall statistics

Fall semester enrollment in our predominantly premedical student cohort gen-chem class is between 750-800 students per year. Students belonging to under-represented minorities or coming from economically disadvantaged backgrounds are considered educational priorities: 250-300 students, roughly a third of the class total, are priority students.

For the past two years, all students, priority and non-priority alike, have been invited to join a pass-fail companion gen-chem service course of the type described above as part of their fall gen-chem studies. Faculty involved in this two-credit fall service course are completely independent from those in the main class: the only information companion course faculty receive about the main class are course notes and documents received by the main chemistry class as a whole.

**Fig 1:**
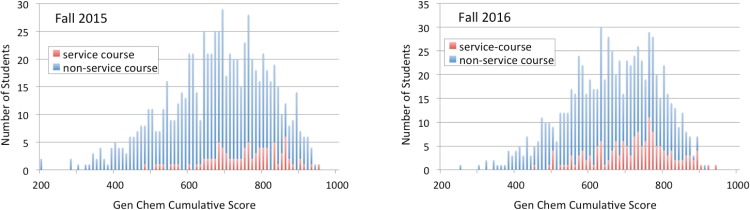
Comparison of service-course and non-service-course gen-chem scores. Results shown are for first semester gen-chem falls 2015-2016. Respective class-wide means for the two years were 663 and 647 (out of 1000 possible pts.). In both years, a disproportionate number of service course attendees scored above the class mean. *P*-values between service course and non-service course students are respectively 1.31 × 10^−6^ and 6.68 × 10^−10^.

**Fig 2:**
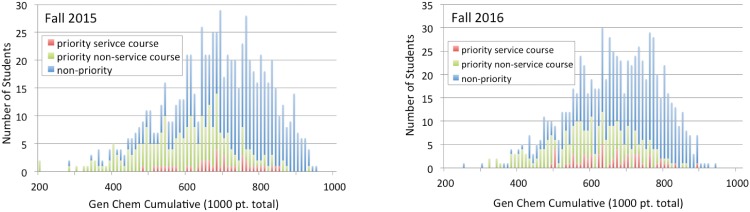
Comparison of priority service-course and priority non-service-course gen-chem scores. Results shown include non-priority students for comparative purposes and are for first semester gen-chem falls 2015-2016. In both years, while a disproportionate number of non-service course priority students scored below the class mean, the service course priority students posted results qualitatively similar to those of the class as a whole. *P*-values between service course priority and non-service course priority students are for both years respectively 5.52 × 10^−6^ and 1.18 × 10^−4^.

The overall results from these two years are tabulated in Tables 1 and 2. Histograms illustrating these results are given in Figs 1 and 2. The results are encouraging, particularly in regards to the outcomes for the priority students. While priority students not in our intensive service course scored -0.63 and -0.58 standard deviations below the class average, priority students in the service class averaged respectively at +0.04 and -0.07 standard deviations compared to the class as a whole. A similar upwards shift among non-priority students can be observed. Non-priority students not in our service course scored, by year, +0.22 and +0.12 standard deviations above the mean compared to, by year, +0.88 and +0.74 standard deviations for those non-priority students in the service course.

**Table 1:** Fall 2015 gen chem performance.

Cohort	Cohort Size	Total Course pts. (z-score)	Std. Dev.
All students	770	663	137
All non-priority	501	702 (+0.28)	126
All priority	269	591 (-0.52)	128
Non-service course, all	679	654 (-0.06)	139
Non-service course, non-priority	454	693 (+0.22)	127
Non-service course, priority	225	576 (-0.63)	129
Service course, all	91	727 (+0.47)	106
Service course, non-priority	47	784 (+0.88)	84
Service course, priority	44	669 (+0.04)	95

**Table 2:** Fall 2016 gen chem performance.

Cohort	Cohort Size	Total Course pts. (z-score)	Std. Dev.
All students	784	647	122
All non-priority	525	675 (+0.23)	116
All priority	259	591 (-0.47)	113
Non-service course, all	630	634 (-0.11)	123
Non-service course, non-priority	429	661 (+0.12)	117
Non-service course, priority	201	577 (-0.58)	115
Service course, all	154	700 (+0.44)	102
Service course, non-priority	96	737 (+0.74)	87
Service course, priority	58	639 (-0.07)	95

Cohort size, which grew by 70% from the first to second year, may continue to grow as the service course becomes better known by the student body. Note that currently participation is ∼20% of the class. However, cohort size is naturally limited by the large 5.5 hour required weekly effort on the part of students. (Overrides for service courses based on credit limits are typically granted without administrative difficulty and have not proved a limitation.) Primary program costs are the establishment of a teaching space lined with whiteboards, a semester faculty teaching line, and peer leader staffing. This last expense is lessened as first-time peer leaders do their work for course credit, as part of the introduction to chemistry pedagogy class. Nonetheless, outside of physical resource limitations, the scalability of the course depends on the availability of well-trained peer course assistants. We have largely relied on students who have excelled themselves in the service course and there are only so many such students.

### Progressive improvement

**Fig 3:**
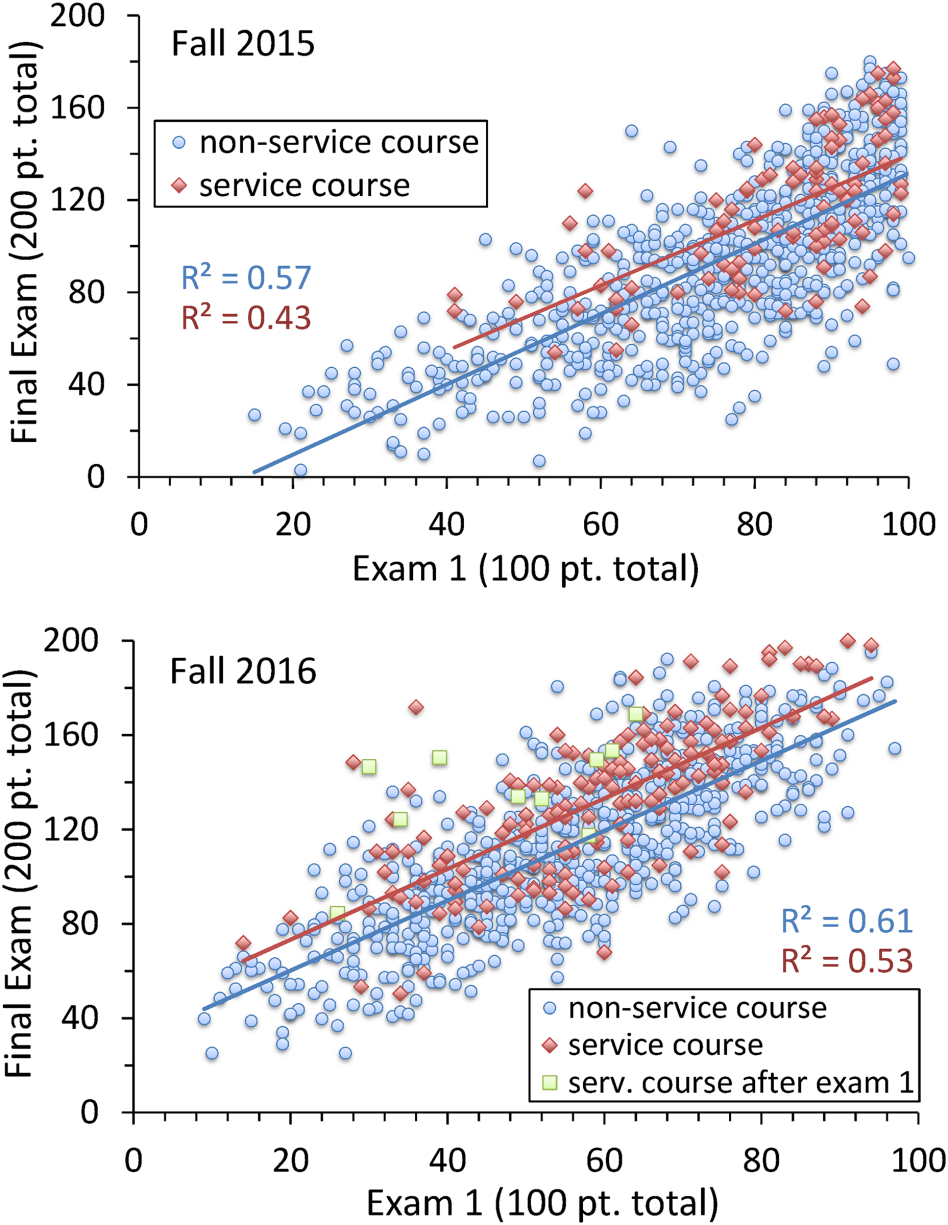
Correlation between first and final exam scores for service-course and non-service-course students. Results shown are the fall gen-chem Exam 1 score and Final Exam score for service and non-service course cohorts. In 2015 and 2016 service course students posted respectively +0.38 and +0.27 standard deviations higher scores on the Final Exam than what would have been predicted based on Exam 1 scores. These numbers measure course improvement in the later half of the course. Note the nearly parallel linear trend lines for the two cohorts, indicating that progressive improvement is independent of class quintile.

While the above results are encouraging, they do not prove service course efficacy. Students self-select to join the program. The reported statistics could be due to the selection process itself, rather than an improvement engendered by the service course. To begin resolving these competing effects, we generated a scatterplot comparing student results on the first gen-chem exam of the fall semester given in October (two-fifths through the semester) with their results on the final fall gen-chem exam given in December, see Fig 3. With R^2^ values ranging from 0.43 to 0.61, the first exam score correlates reasonably well with the final exam score.

As inspection of the figure shows, students in the service course fare significantly better with respect to their final exam score than what would have been indicated based solely on their first exam results. We consider the 2016 data first. For the service and non-service cohorts, linear fits between the two exams run roughly parallel with one another. However, the service-course students fit lies on the average 13.2 final exam points above non-service course students. With a standard deviation on the final exam of 34.8 points, 2016 service-course students exhibit an improvement on the average of 0.38 standard deviations higher with respect to the class as a whole.

Similar results are found in 2015. In this year, however, the gen-chem Exam 1 did not achieve a bell-shaped distribution; rather, due to the ease of the exam, the grade distribution arbitrarily truncates at 100 points. Overall, this Exam 1 is not as strong a predictor for higher scoring students: an average improvement of only 9.9 points on a final exam which had a class-wide standard deviation of 37.0 points is observed. In 2015, service-course students’ measured improvement lies 0.27 standard deviations above non-service course students.

**Fig 4:**
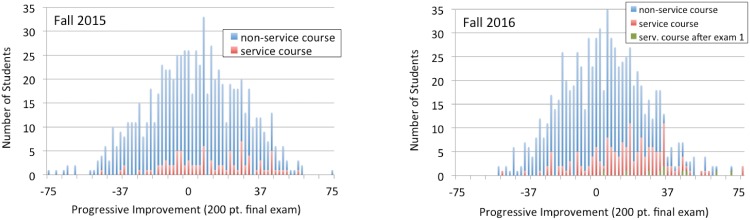
Progressive improvement of service-course and non-service-course students. The +/- shift (progressive improvement) by student Final Exam scores from what would have been predicted based on Exam 1 scores alone. By definition, non-service course students have a +/- mean of zero points. For 2015 and 2016, the difference between service and non-service course student populations corresponds to respectively *p* = 1.9 × 10^−4^ and 1.6 × 10^−10^.

Both data sets were converted into histogram form, (Fig 4). In preparing this figure, we used non-service course data to calculate a trend line predicting final exam score based on a student’s first exam score. We then determined, for all students, the difference between a given student’s actual final-exam score and the same student’s predicted score. A positive deviation measures progressive improvement during the second half of the fall semester; negative values indicate regressive decline.

As Fig 4 shows, in both years, service-course students are distinguished by strong progressive improvement when compared to non-service-course students. The *p*-value measuring differences in service and non-service-course student populations are, by year, respectively 1.9 × 10^−4^ and 1.6 × 10^−10^. Unlike the histograms shown in the previous section, student data presented in this way factor in variations of a given student with respect to prior work-ethic, prior-chemistry knowledge, and pre-existing STEM intellectual affinity. We conclude that the fall service course significantly improves student gen-chem performance.

The measured progressive improvement of +0.38 and +0.27 derives from skills acquired after Exam 1: Progressive improvement from the start of the semester to Exam 1 remains unmeasured. With a +0.38 standard deviation in progressive improvement from Exam 1 to the Final Exam, and with a course-overall shift of +0.55 standard deviations, we deduce that 2016 students, who selected the service course, had neither substantially greater prior-chemistry knowledge nor higher pre-existing STEM intellectual affinity.

The 2016 data has a further point of interest. Ten students decided to join the service course after taking the main course’s first exam. (In 2015, there were no such students, presumably due to the ease of the 2015 first exam.) As these ten 2016 students could only complete 80% of the given activities after the first exam and therefore did not meet the overall service course pass requirement, they are not counted among the service class cohort. Nonetheless, these students performed well on the final exam at a much higher value than that predicted by their first exam results. The two-tailed Student’s *t*-test comparing these ten students’ progressive improvement to the non-service-course cohort results in a *p* = 4.4 × 10^−4^.

The results of Fig 3, demonstrate an increase in student performance almost independent of class quintile. Thus, the service course fulfills the promise of a Treisman-like model in that the interventions are open to multiple demographic groups and they aid all enrolled students. For a first semester gen-chem service course, with a composition of students who are experiencing a university STEM class for the first time, the raising of students scores, irrespective of incoming ability, should not be viewed as a defect but as a virtue. That being said, it should be examined as to whether with an efficient introductory service courses in place, the gap between priority and non-priority students in both performance and placement might narrow later in their college careers.

### Priority students

The progressive improvement metric discussed above is marred by statistical variations in individual exam results. Fortunately, for service-course priority students, a more accurate metric is available. In both 2015 and 2016, roughly half the service-course priority students took an intensive six-week chemistry summer bridge program (taught by one of the authors (SL) and Dr. Steven Johnson of the Cornell Learning Services Center) directly prior to their fall gen-chem class. This summer bridge course is taught with the principal objective of preparing selected priority students for fall gen-chem.

In Table 3, we directly compare total course scores from this summer-bridge course with those obtained in the subsequent fall semester. (Also included in this table as a separate line-item are results for service-course priority students who were not part of the summer-bridge program.) In both 2015 and 2016, both summer-bridge attendees and non-attendees had very different fall gen-chem outcomes. Summer-bridge service-course students scored respectively, by year, +0.04 and -0.04 standard deviations with respect to the class mean. Summer-bridge non-service-course students scored, by year, -0.73 and -0.78 standard deviations with respect to the class mean, 0.7 standard deviations below their service-course counterparts.

**Table 3:** Priority student performance by cohort.

Course Cohort	Cohort Size	Total Course pts. (z-score)	Std. Dev.
2015 summer-bridge, fall service	20	668 (+0.04)	90
2015 non-summer-bridge, fall service	24	669 (+0.04)	100
2015 summer-bridge, fall non-service	47	563 (-0.73)	134
2016 summer-bridge, fall service	25	642 (-0.04)	68
2016 non-summer-bridge, fall service	33	638 (-0.07)	112
2016 summer-bridge, fall non-service	29	552 (-0.78)	126

**Fig 5:**
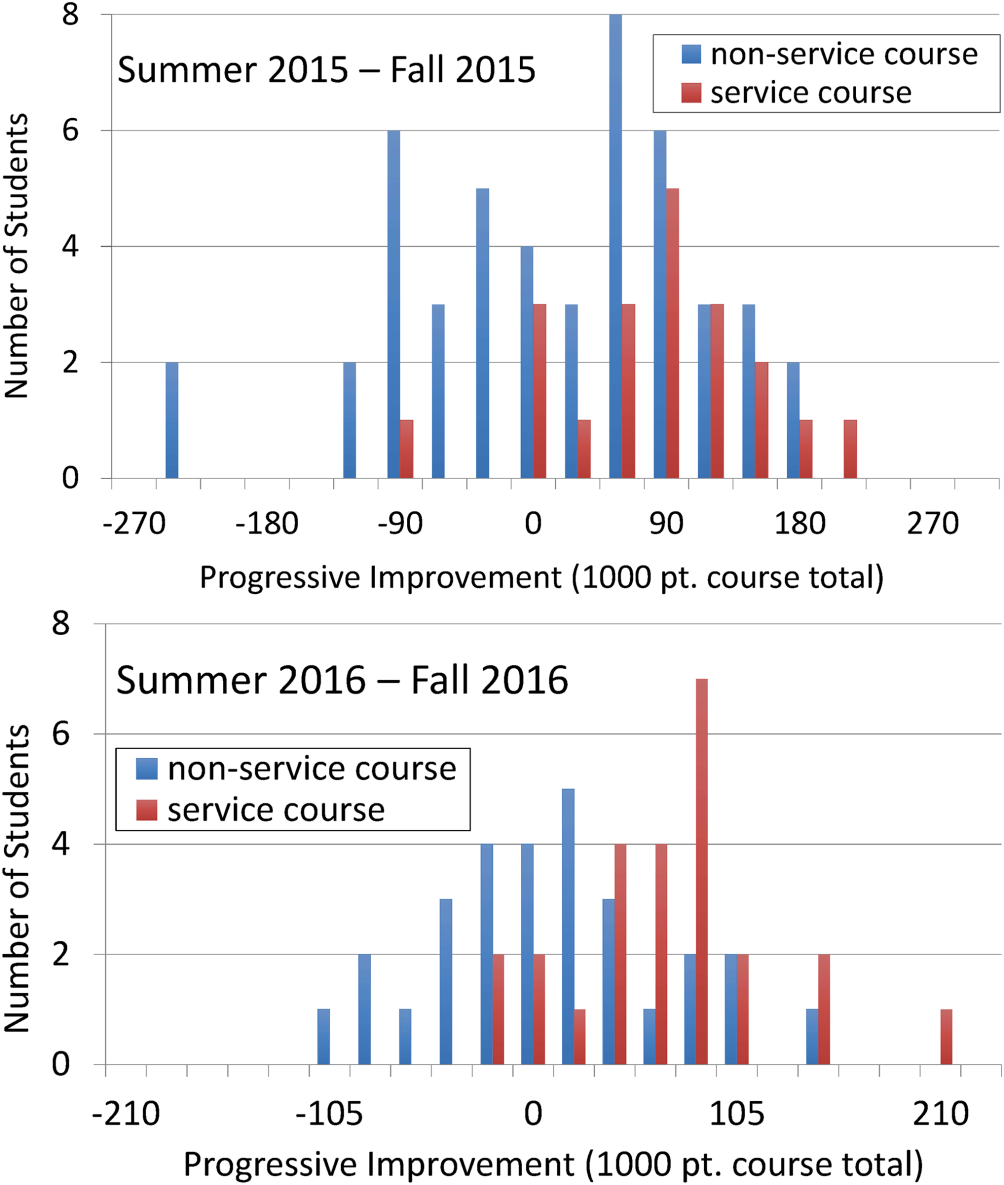
Progressive improvement of summer-bridge service-course and non-service-course students in fall gen-chem. The +/- shift (progressive improvement) in student fall gen-chem total score from what would have been predicted based on summer bridge course totals. By definition, fall gen-chem non-service course students have a +/- mean of zero points. For 2015 and 2016 the difference between service and non-service course student populations corresponds to respectively *p* = 4.5 × 10^−3^ and 5.4 × 10^−4^.

Histograms comparing individual summer-bridge total scores with the same individual’s total fall course score are presented in Fig 5. Progressive improvement is now calculated by comparing a given student’s actual fall gen-chem course total with the score which would have been predicted based on that same student’s summer-bridge course total (predicted scores are derived solely from non-service-course student data). Scatterplots of the same progressive improvement data are given in S1 Fig in the supplementary material.

In 2015 and 2016 summer-bridge service-course students scored respectively 63 and 58 points above their predicted cumulative scores, respectively 0.46 and 0.48 course-wide standard deviations above their summer-bridge non-service-course counterparts. The differences between the two populations correspond to *p*-values of 4.5 × 10^−3^ and 5.4 × 10^−4^. Despite small cohort sizes, a statistically different outcome between summer-bridge-course service-course and non-service-course attendees is apparent.

A future study which analyzes in depth the demographic breakdown of Cornell’s priority student cohort, could help distinguish the overlapping socioeconomic and ethnic factors in the above results. A revised summer gen-chem bridge-program, based more heavily on the Treisman methodology, is currently in progress. However, it should be noted, that the effects of the summer bridge course alone are difficult to ascertain with the data presented here because participation in the service course by a subset of motivated summer bridge students will remove a group from the control (summer bridge no service course) possibly biased toward improved outcomes. There may be strong social incentives for teams of achieving students to matriculate from the summer bridge into the service course.

### Progressive retention

A natural question is whether gains in gen-chem test scores deriving from the service-course improvement are retained to any extent in later chemistry courses. We have therefore tracked the fall 2015 gen-chem service-course cohort to fall 2016 organic chemistry (orgo-chem) one year later. These results are presented in Table 4. Of the 679 non-service-course students (the service course referred to here being the gen-chem service course), 361 of these students, (53%), were enrolled in orgo-chem one year later. By contrast, retention for gen-chem service-course students one year later was higher at 74%. As Table 4 shows, differences in retention rates are even more striking among priority students (47% vs. 77%).

**Table 4:** 2015 fall gen-chem retention in 2016 fall orgo-chem.

Cohort	Original Cohort size	Cohort Retained (% of original)	Orgo-Chem. Cumulative Total (z-score)	Std. dev.
Gen-chem, all	770	428 (55.6%)	335	78
Gen-chem, non-priority	501	289 (57.7%)	352 (+0.21)	72
Gen-chem, priority	269	139 (51.7%)	302 (-0.43)	81
Non-service course, all	679	361 (53.2%)	333 (-0.03)	78
Non-service course, non-priority	454	256 (56.4%)	348 (+0.17)	71
Non-service course, priority	225	105 (46.7%)	296 (-0.51)	83
Service course, all	91	67 (73.6%)	348 (+0.16)	77
Service course, non-priority	47	33 (70.2%)	377 (+0.53)	70
Service course, priority	44	34 (77.3%)	320 (-0.19)	75
CHAMPS	20	19 (95.0%)	361 (+0.32)	59

Relevant to this retention study is a post-freshman summer bridge program, introduced to Cornell roughly at the same time as the intensive gen-chem service course. For the past two years, roughly 20 priority students are selected each year as Cornell-Howard Hughes Accelerated Medical Program Scholars (CHAMPS) (in 2015, of the nineteen CHAMPS, five and fourteen of these students came from the non-service and service course cohorts respectively). As part of this program, CHAMPS receive a six week post-freshman summer course in orgo-chem. As CHAMPS are selected from among priority students who have already declared their intention of taking orgo-chem the following fall, the CHAMPS program does not directly alter orgo-chem retention rates. We therefore include CHAMPS in our retention statistics but not in our statistical evaluations of orgo-chem performance.

**Fig 6:**
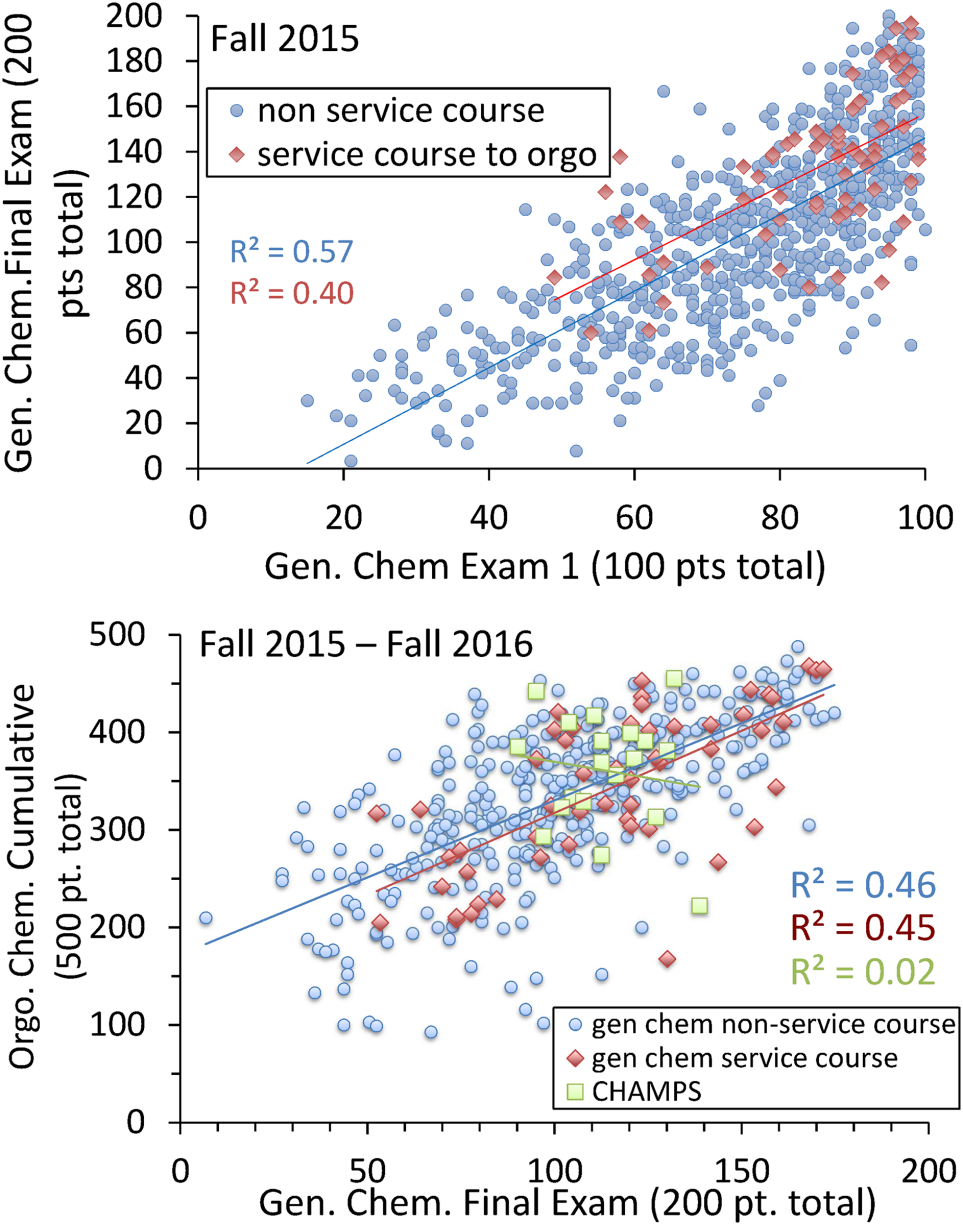
Correlation between final gen-chem and subsequent orgo-chem exam scores. Left: Gen-chem Exam 1 and Final Exam scores for fall gen-chem service course (continuing on to organic chemistry) and for full non gen-chem service course cohorts. Right: Fall gen-chem Final Exam score and orgo-chem total score for continuing-in-chemistry cohorts. A portion of the progressive improvement gained by service course students, in going from gen-chem Exam 1 to the gen-chem Final Exam, is retained in passing from the fall gen-chem final to fall orgo-chem.

In Fig 6, we consider just the gen-chem service cohort of students who continued on to orgo-chem the following year. As before, we use gen-chem Exam 1 scores as a predictor of gen-chem Final Exam scores. The continuing service course student cohort show a +0.32 standard deviation progressive improvement relative to the non-service cohort (this number being the ratio of the average of 12.0 pts. increase in final exam scores divided by the class-wide exam standard deviation of 37.0 pts).

The right panel of Fig 6 then goes on to measure what portion of this progressive improvement is retained one year later in orgo-chem. Here we use fall 2015 gen-chem Final Exam scores as predictors of fall 2016 orgo-chem cumulative exam scores. A drop of -0.14 standard deviation in orgo-chem scores is observed (this number being the ratio of a -11 pt drop on cumulative orgo-exam scores divided by a class-wide 78 pt. cumulative exam score standard deviation). This progressive retention, roughly half the value of the prior progressive improvement, is in agreement with the statistics presented in Tables 1 and 4, where for non-priority students the initial elevation in gen-chem results of +0.66 (0.88-0.22) standard deviations was seen to reduce by half, a +0.36 (0.53-0.17) elevation for orgo-chem.

S2 Fig in the supplementary material presents the data in histogram form. The histograms show graphically the statistical difference in progressive improvement in going from gen-chem Exam 1 to the gen-chem final and the progressive retention of this improvement in passing from the gen-chem final exam to first semester orgo-chem. The difference in progressive improvement populations is statistically significant at *p* = 5.6 × 10^−4^. But the difference in populations for progressive retention is statistically insignificant at *p* = 0.40. One year after the initial teaching intervention, non-priority students continue to reap benefits. We note that for priority students, the CHAMPS program is invaluable in continued progressive retention, and that no data have been presented for additional longitudinal benefits [24].

## Conclusion

S1 Table and the accompanying analysis in the supplementary material gives context to our results. It highlights a startling difference between the earlier Treisman mathematics programs and other more recent gen-chem educational interventions at R1 universities [7, 8, 18, 19, 23, 25–31]. The Treisman Emerging Scholars Programs raised average student calculus grades by two-fifths to three-quarters of a letter grade with respect to the class as a whole. Less quantitative gateway courses, biology, biochemistry, and organic chemistry have been able to post similar increases [32–36]. But as S1 Table shows, as a standard bearer for a quantitative science gateway course, gen-chem service courses (as opposed to bridge courses [8, 28]) have not been able to obtain equally strong results, with a few exceptions [7, 31, 37].

Of particular interest in this table are the results from the SAGE program at Duke and the PLTL program at Washington [7, 8, 31]. Both programs draw fully from recent educational advances. The SAGE program bridge course brings at-risk students up to the same achievement levels as the not-at-risk students, but the SAGE service course does not report similar progress. The Washington program is able to achieve significant improvement through a gen-chem service course alone. To what, can the differences in gen-chem service course results be attributed?

A critical examination of three well-known gen-chem textbooks show only 1-2% of the problems access the upper levels of Bloom’s taxonomy [38, 39]. Could the difference in gen-chem results be in the level of posed problems? That is, could different gen-chem service courses achieve different results due to the cognitive sophistication of the posed worksheet problems? In this paper, we hypothesized that a modern gen-chem service program needs to emulate the Treisman Emerging Scholars Program by (1) adopting modern transformative teaching methods, (2) concentrating on cognitively challenging worksheets, and (3) increasing the number of contact hours. The results of this study are that such a program allows not just a strong upwards trend for the lowest quintile students (as found in the Washington University study), but also creates similar upwards shifts for upper-quintile cohorts as well.

At a school such as Cornell, the first semester general chemistry GPA class average is 2.8. Nationally, the average GPA of admitted medical school students ranges from 3.4 for under-represented minorities to 3.7 for Asian-Americans [40]. At Cornell, the 0.5 to 0.7 standard deviations increase reported in this paper leads, in general, to numerically similar increases in GPA. Placing these numbers together, the reported increase puts a significant fraction of the pre-med cohort, priority and non-priority alike, on track for a career as a doctor in the health and allied sciences.

Antonin Scalia, the late Supreme Court justice, citing the mismatch hypothesis, [41] famously said “There are those who contend that it does not benefit African-Americans to get them into the University of Texas (an R1 university) where they do not do well, as opposed to having them go to a less-advanced school, a slower track school where they do well…. One of the briefs pointed out that most of the black scientists in this country don’t come from schools like the University of Texas, they come from lesser schools where they do not feel that they’re being pushed ahead in classes that are too fast for them [42].” Analysis of graduation data both at the regional level (for California) and at the national level clearly refute the mismatch hypothesis. [43, 44]

While this paper provides no new demographic data, it makes one additional point: college education is not immutable. The university gen-chem pedagogy of ten years ago is not the one of today. All student cohorts, priority and non-priority alike, can be taught effectively at an R1 university in such a manner that they achieve success in general chemistry, a course viewed by many as the gateway course to a STEM career.

## Supporting information

S1 Fig Correlation between summer bridge and fall gen-chem course totals for fall gen-chem service and non-service course cohorts. Both cohorts received the same summer bridge course but, in 2015 and 2016, fall gen-chem service course students scored respectively 63 and 58 pts. more than what would have been expected based on their summer bridge course work. Note the greatest upwards shift in fall gen-chem scores are for intermediate scoring summer-bridge students.
(EPS)

S2 Fig Progressive improvement for fall gen-chem and progressive retention for subsequent fall orgo-chem for service-course and non-service-course students. Left: The +/- shift, progressive improvement, in gen-chem Final Exam scores for service (continuing on to orgo-chem) and non-service course students from those predicted by gen-chem Exam 1 scores. Right: The +/- shift, progressive retention, (for the continuing cohort) in orgo-chem cumulative scores from those predicted by previous gen-chem Final Exam scores. The difference in progressive improvement between the two populations is significant, *p* = 5.6 × 10^−4^; those of progressive retention are insignificant, *p* = 0.40.
(EPS)

S1 File Service course worksheets and practice exams. (PDF)

S2 File Meta-analysis of gen-chem teaching initiatives at R1 universities. (PDF)

S1 Table Summary of teaching initiatives at R1 universities: Emerging Scholars programs vs. gen-chem programs. (PDF)

## References

[1] Chubin DE, May GS, Babco EL. Diversifying the Engineering Workforce. J Eng Educ. 2005;94:73–86. 10.1002/j.2168-9830.2005.tb00830.x

[2] National Research Council. Expanding Underrepresented Minority Participation: America’s Science and Technology Talent at the Crossroads. Washington, D.C.: The National Academies Press; 2011. 22379652

[3] Mazur E. Peer Instruction: A User’s Manual. Reading, MA: Addison-Wesley; 1996.

[4] Gosser DK Jr, Kampmeier JA, Varma-Nelson P. Peer-Led Team Learning: 2008 James Flack Norris Award Address. J Chem Educ. 2010;87(4):374–380. 10.1021/ed800132w

[5] Hanson DM. Foundations of Chemistry: Applying POGIL Principles. 4th ed Lisle. IL: Pacific Crest; 2010.

[6] Slavich GM, Zambardo PG. Transformational teaching: Theoretical underpinnings, basic principles, and core methods. Educ Psych Rev. 2012;24:569–608. 10.1007/s10648-012-9199-6 PMC3498956 23162369

[7] Shields SP, Hogrebe MC, Spees WM, Handlin LB, Noelken GP, Riley JM, et al A transition program for underprepared students in general chemistry: diagnosis, implementation, and evaluation. J Chem Educ. 2012;89:995–1000. 10.1021/ed100410j

[8] Hall DM, Curtin-Soydan AJ, Canelas DA. The science advancement through group engagement program: Leveling the playing field and increasing retention in science. J Chem Educ. 2014;91:37–47. 10.1021/ed400075n

[9] Kirschner PA, Sweller J, Clark RE. Why minimal guidance during instruction does not work: An analysis of the failure of constructivist, discovery, problem-based, experiential, and inquiry-based teaching. Educational Psychologist. 2006;41(2):75–86. 10.1207/s15326985ep4102_1

[10] Freeman S, Eddy SL, McDonough M, Smith MK, Okoroafor N, Jordt H, et al Active learning increases student performance in science, engineering, and mathematics. Proc Nat Acad Sci. 2014;111:8410–8415. 10.1073/pnas.1319030111 24821756 PMC4060654

[11] Olson S, Riordan DG. Engage to Excel: Producing One Million Additional College Graduates with Degrees in Science, Technology, Engineering, and Mathematics. Washington, D.C.: President’s Council of Advisors on Science and Technology; 2012.

[12] Graham MJ, Frederick J, Byars-Winston A, Hunter AB, Handelsman J. Increasing persistence of college students in STEM. Science. 2013;341:1455–1456. 10.1126/science.1240487 24072909 PMC10167736

[13] Gándara P. Priming the Pump: Strategies for Increasing the Achievement of Underrepresented Minority Undergraduates. New York: The College Board; 1999.

[14] Alexander C, Chen E, Grumbach K. How leaky is the health career pipeline? Minority student achievement in college gateway courses. Acad Med. 2009;84:797–802. 10.1097/ACM.0b013e3181a3d948 19474563

[15] Fullilove RE, Treisman PU. Mathematics achievement among African American Undergraduates at the University of California, Berkeley: An Evaluation of the Mathematics Workshop Program. J Negro Educ. 1990;59:463–478. 10.2307/2295577

[16] Treisman U. Studying students studying calculus: A look at the lives of minority mathematics students in college. College Math J. 1992;23:362–372. 10.2307/2686410

[17] Alexander BB, Burda AC, Millar SB. A community approach to learning calculus: Fostering success for underrepresented ethnic minorities in an Emerging Scholars program. J Women Minor Sci Eng. 1997;3:145–159. 10.1615/JWomenMinorScienEng.v3.i3.20

[18] Murphy TJ, Stafford KL, McCreary P. Subsequent course and degree paths of students in a Treisman-style workshop calculus program. J Women Minor Sci Eng. 1998;4:381–396. 10.1615/JWomenMinorScienEng.v4.i4.50

[19] Moreno SE, Muller C, Asera R, Wyatt L, Epperson J. Supporting minority mathematics achievement: The Emerging Scholars Program at the University of Texas at Austin. J Women Minor Sci Eng. 1999;5:53–66. 10.1615/JWomenMinorScienEng.v5.i1.40

[20] Hsu E, Murphy TJ, Treisman U. Supporting high achievement in introductory mathematics courses: What we have learned from 30 years of the Emerging Scholars Program In: Carlson MP, Rasmussen C, editors. Making the Connection: Research and Teaching in Undergraduate Mathematics. Washington, DC: Mathematical Association of America; 2008 p. 205–222.

[21] Barr DA, Gonzalez ME, Wanat SF. The leaky pipeline: Factors associated with early decline in interest in premedical studies among underrepresented minority undergraduate students. Acad Med. 2008;83:503–511. 10.1097/ACM.0b013e31816bda16 18448909

[22] Barr DA, Matsui J, Wanat SF, Gonzalez ME. Chemistry courses as the turning point for premedical students. Adv Health Sci Educ. 2010;15:45–54. 10.1007/s10459-009-9165-3 PMC2814029 19504170

[23] Nocera DC, Harrison JF, Reed DA, Roberts CH. Enhanced Performance in Chemistry by Minorities at the University Level. J Chem Educ. 1996;73:1131–1137. 10.1021/ed073p1131

[24] Lewis SE. Investigating the longitudinal impact of a successful reform in general chemistry on student enrollment and academic performance. J Chem Educ. 2014;91:2037–2044. 10.1021/ed500404q

[25] Pickering M. Helping the High Risk Freshman Chemist. J Chem Educ. 1975;52:513–514. 10.1021/ed052p512

[26] Dougherty RC, Bowen CW, Berger T, Rees W, Mellon EK, Pulliam E. Cooperative learning and enhanced communication: Effects on student performance, retention, and attitudes in general chemistry. J Chem Educ. 1995;72:793–797. 10.1021/ed072p793

[27] Gutwill-Wise JP. The impact of active and context-based learning in introductory chemistry courses: An early evaluation of the modular approach. J Chem Educ. 2001;78:684–690. 10.1021/ed078p684

[28] Bentley AB, Gellene GI. A six-year study of the effects of a remedial course in the chemistry curriculum. J Chem Educ. 2005;82:125–130. 10.1021/ed082p125

[29] Oliver-Hoyo MT, Allen D, Hunt WF, Hutson J, Pitts A. Effects of an active learning environment: Teaching innovations at a Research 1 Institution. J Chem Educ. 2004;81:441–448. 10.1021/ed081p441

[30] Lewis SE, Lewis JE. Seeking effectiveness and equity in a large college chemistry course: an HLM investigation of peer-led guided inquiry. J Res Sci Teach. 2008;45:794–811. 10.1002/tea.20254

[31] Hockings SC, DeAngelis KJ, Frey RF. Peer-led team learning in general chemistry: Implementation and evaluation. J Chem Educ. 2008;85(7):990–996. 10.1021/ed085p990

[32] Tien LT, Roth V, Kampmeier JA. Implementation of a Peer-Led Team Learning instructional approach in an undergraduate organic chemistry course. J Res Sci Teach. 2002;39:606–632. 10.1002/tea.10038

[33] Matsui J, Liu R, Kane CM. Evaluating a science diversity program at UC Berkeley: More questions than answers. Cell Bio Educ. 2003;2:117–121. 10.1187/cbe.02-10-0050 12888847 PMC162187

[34] Anderson WL, Mitchell SM, Osgood MP. Comparison of student performance in cooperative learning and traditional lecture-based biochemistry classes. Biochem and Mol Bio Educ. 2005;33:387–393. 10.1002/bmb.2005.49403306387 21638607

[35] Haak DC, HilleRisLambers J, Pitre E, Freeman S. Increased structure and active learning reduce the achievement gap in introductory biology. Science. 2011;332:1213–1216. 10.1126/science.1204820 21636776

[36] Stockwell BR, Stockwell MS, Cennamo M, Jiang E. Blended learning improves science education. Cell. 2015;162:933–936. 10.1016/j.cell.2015.08.009 26317458

[37] Ryan MD, Reid SA. Impact of the flipped classroom on student performance and retention: A parallel controlled study in general chemistry. J Chem Educ. 2016;93:13–23. 10.1021/acs.jchemed.5b00717

[38] Dávila K, Talanquer V. Classifying end-of-chapter questions and problems for selected general chemistry textbooks used in the United States. J Chem Educ. 2010;87(1):97–101. 10.1021/ed8000232

[39] Bloom BS, Engelhart MD, Furst EJ, Hill WH, Krathwohl DR. Taxonomy of Educational Objectives: The Classification of Educational Goals Handbook I: Cognitive Domain. New York: David McKay; 1956.

[40] Association of American Medical Colleges. Applicants and matriculants data; 2015.

[41] Sander R, T S Jr. Mismatch: How Affirmative Action Hurts Students It’s Intended to Help, and Why Universities Won’t Admit It. New York: Basic Books; 2012.

[42] Ross J. Antonin Scalia’s strange idea that blacks might do better in “less advanced schools”. Washington Post. 2015;Dec. 10.

[43] Kurlaender M, Grodsky E. Mismatch and the Paternalistic Justification for Selective College Admissions. Sociology of Education. 2013;86:294–310. 10.1177/0038040713500772

[44] Carnevale AP, Strohl J, and Van Der Werf M. The Concept of “Mismatch” at Play in the Supreme Court Fisher Decision is Empirically Unsound. Georgetown: Georgetown University Center on Education and the Workforce; 2016.

[45] Novak JD, Gowin DB. Learning How to Learn. Cambridge: Cambridge University Press; 1984.

[46] Zumdahl SS, Zumdahl SA, and DeCoste DJ. Chemistry 10th Edition. Boston: Cengage Learning; 2018.

[47] Petrucci RH, Herring FG, Madura JD, and Bissonnette C. General Chemistry: Principles and Modern Applications 11th Edition. Canada: Pearsons; 2017.

