# Supplementary material for: Patching a leak in an R1 university gateway STEM course
Source: PLoS One. 2018 Sep 6;13(9):e0202041. doi: 10.1371/journal.pone.0202041 (PMC6126828; doi:10.1371/journal.pone.0202041)
Supplement: S1 File — (PDF) [file pone.0202041.s003.pdf]

Service course worksheets and examinations

Table of contents:

Introduction **2**
Workshops .... **3 – 37**
1.1 Workshop 1: Moles and unit conversion .... 3 – 4
1.2 Workshop 2: Proportionality .... 5 – 6
1.3 Workshop 3: Reactions and combustion analysis .... 7 – 8
1.4 Workshop 4: Limiting reagents .... 9 – 10
1.5 Workshop 5: Calculator free chem-math I .... 11 – 12
1.6 Workshop 6: The stoichiometry diagram and stoichiometry problem types .... 13 – 14
1.7 Workshop 7: Core stoichiometry problems .... 15 – 16
1.8 Workshop 8: A-level stoichiometry problems .... 17 – 18
1.9 Workshop 9: The cathode-ray and oil-drop experiments and mass spectrometry .... 19 – 20
1.10 Workshop 10: Electronegativity, oxidation states, and reduction states .... 21 – 22
1.11 Workshop 11: Balancing redox reactions and compound names .... 23 – 24
1.12 Workshop 12: Classifying reactions .... 25 – 26
1.13 Workshop 13: Reaction chemistry II .... 27 – 28
1.14 Workshop 14: Reactions review I .... 29 – 30
1.15 Workshop 15: A-level stoichiometry problems II .... 31 – 32
1.16 Workshop 16: Overview for balancing chemical reactions 33
1.17 Workshop 17: Percentages, ratios, and averages in gen chem .... 34 – 35
1.18 Workshop 18: A deeper understanding of the experiments .... 36 – 37
Practice prelims .... **38 – 47**
1.19 Chem 1070 Practice prelim 1-1 .... 38 – 39
1.20 Chem 1070 Practice prelim 1-2 .... 40 – 41
1.21 Chem 1070 Practice prelim 1-3 .... 42 – 43
1.22 Chem 1070 Practice prelim 1-4 .... 44 – 45
1.23 Chem 1070 Practice prelim 1-5 .... 46 – 47
Comparison of the cognitive requirements of service course worksheets to text and problems in current university level general chemistry textbooks .... **48 – 52**

1 Introduction

With the idea of fleshing out the pedagogical nature of our service course, this section presents all service course worksheets and practice exams given to students up to the first fall main class gen-chem examination date in October. At no time do service course instructors consult with main course instructors. Contact between main and service course is limited to main course documents available to all gen-chem students.

1. **Service course components**

   For students, the service course has the following components:

   (a) a weekly two-hour workshop in which groups of twelve to twenty service course students, either singly or in pairs, solve problems, simultaneously, on ten adjacent large whiteboards. Monitoring their work are two undergraduate course assistants, selected these days from former service course students who, in a previous year, scored in the top quintile of the fall gen-chem class. Attention is paid to demographic diversity among this teaching cohort.

   (b) a weekly ninety minute scored practice exam taken by the service class as a whole, which is used by student and teaching staff alike in monitoring service course student progress.

   (c) a weekly two-hour flipped class, which the professor of the class (SL) gives to groups of 50-100 service course students at a time.

   (d) Starting two to three weeks prior to the main course gen-chem exam, the opportunity to do further workshop problems is afforded to the service course students. Service course students who have scored poorly on the scored practice prelims are especially encouraged to attend, but attendance at these extra sessions is open to the service class as a whole.

   In addition to the activities mentioned above, required activities for course assistants include:

   (a) a weekly three-and-a-half hour meeting where course assistants score practice prelims; work out, on whiteboards, the weekly workshop questions; and develop a lesson plan for the following week.

   (b) Prior to the beginning of the course, a weekend-long training program devoted to teaching methods for a diverse student body is given to the upcoming semester's service course assistants.

2. **Service course material**

   Material given to service class students as they prepare for the first main-class gen-chem examination are presented below. Presented are the eighteen workshop worksheets and the five scored practice exams. Each workshop presents two worksheets (worksheets 1-2, 4-13: worksheet 3 being given to students but not actually worked on in workshop). A further five review worksheets (worksheets 14-18) are given to students one to three weeks before the actual first main class gen-chem exam date.

   To the reader interested in the specifics of gen-chem pedagogy, the presented material diverges from standard gen-chem textbooks in several respects:

   (a) Equal pedagogical weight is given to unit conversion and proportionality thinking (worksheets 1 and 2).

   (b) Students develop a number sense through working worksheets where calculators, and sometimes even hand-written arithmetic, are not allowed (worksheets 5 and 6).

   (c) The treatment of stoichiometry problems is adapted from concept maps (worksheets 6, 7, and 8). (For an introduction to concept maps see reference 42 of the main paper.)

   (d) A proportionality-based treatment of the limiting reagent problem is given (worksheet 4).

   (e) Concepts from introductory general physics are presented (worksheets 9 and 18).

   (f) A systematic approach to balancing *all* chemical reactions is given (worksheets 3, 10-11, and 16).

   (g) A hierarchical classification of reactions based on redox, acid-base, dissolution/precipitation and insertion/elimination reactions is presented (worksheets 10, 12, 13, and 14).

   (h) Additional cognitively challenging stoichiometry problems, extending those given in most modern gen-chem textbooks, are given (worksheet 15 and selected questions in worksheet 17).

1.1 Chem 1070 workshop 1: Moles and unit conversion

1. **Oxygen in your lungs and moles**

   (a) Oxygen is an element, one of the 90+ atomic elements listed in the periodic table. When we breathe in oxygen, we are breathing in oxygen atoms. And in our breath these oxygen atoms are bound together in pairs, as oxygen molecules, O_2.

   (b) When a person takes a deep breath, their lungs fill with approximately 3.4×10^{22} oxygen molecules. This is an absurdly large number. It would quickly become tiresome to continuously use numbers as large as these whenever we wanted to describe the number of atoms in important places like our lungs. We could divide this number by twelve and that would give the number of dozens of oxygen molecules the lungs are holding, but that would be 2.8×10^{21} dozens, which isn't much more useful than the original number.

   (c) Because chemists deal with such large numbers of molecules and atoms at a time, they group the molecules and atoms by *moles*, rather than dozens or hundreds. A mole is a specific number, just like a dozen is 12. But a mole, often called a mol, is equal to 6.022×10^{23}. (The number a mole is also called *Avogadro's number.*)

   (d) We can find the number of moles of oxygen molecules exactly the same way we find the number of dozens. We divide the number of molecules, 3.4×10^{22} by 6.022×10^{23} and get 0.056 moles. The number of moles of oxygen molecules, 0.056 moles, is a lot easier number to work with than the earlier numbers! Writing down the math we used, we find:

$$3.4 \times 10^{22}\,\text{molecules} \times \frac{1\,\text{mole}}{6.022 \times 10^{23}\text{molecules}} = 0.056\,\text{mole}$$

   (e) Based on the above, please answer the following questions:

      i. How many moles of individual oxygen atoms would be contained in the same deep breath described above?

      ii. How many M&Ms are there in 1.6 moles of M&Ms?

      iii. Is the mol a good unit to use when describing the number of people on the Earth?

      iv. Which is more money, a mol of pennies or a million billion dollars?

2. **Moles and mass**

   (a) So when we talk about a mol, we're talking about a specific quantity. But we can all clearly see that a mol of people does not weigh the same amount as a mol of M&Ms, right? The same goes for different elements. What makes an element different from another element is its number of protons. And what makes the weight of an element different from the weights of other elements are different numbers of protons and neutrons.

   (b) An important aside here is that an atom has three components: protons, neutrons and electrons. As we said above, the weight for an element is mainly due to the protons and neutrons in the atom. That's because electrons are very light: 1800 times lighter than the protons and neutrons.

   (c) This atomic weight is recorded in the periodic table. As protons and neutrons have small masses, 1.7×10^{-27} kilograms, the periodic table keeps track of their masses on a per mol basis. Carbon with exactly 6 protons and on the average roughly 6 neutrons per atom has an *atomic mass* of 12.01 grams per mole.

   (d) Based on the above, please answer the following questions:

      i. What is the molar mass of water, H_2O, ie., what is the mass of a mole of H_2O molecules?

      ii. The molar mass of carbon, C, is 12.01 g/mol. Could you use this number to find the mass of a single carbon atom?

      iii. Boron, B, has a larger molar mass than helium, He. What does this tell you about the relative weights of a boron and a helium atom?

iv. Can you use molar masses to explain why a block of gold, Au, is heavier than the same sized block of iron, Fe?

v. In one mol of KBr molecules, how many moles of potassium, K, atoms and how many moles of bromine, Br, atoms are there?

vi. Approximately how many moles of helium, He, atoms would you need to have the same weight as 1 mol of sodium, Na atoms?

3. **Unit conversion**

(a) Chemistry involves a large number of units, and sometimes you're given units that you don't want. Luckily, we can convert between these units. For example, if you're told that you have 4 and half dozen eggs, you can find out how many total eggs you have by using the conversion principle:

$$4.5\ \text{dozen} \times \frac{12\ \text{eggs}}{1\ \text{dozen}} = 54\ \text{eggs}$$

(b) We use these sort of conversions every day. Moneywise, you could ask how many quarters would it take to make \$712? We would figure it as follows:

$$712\ \text{dollars} \times \frac{4\ \text{quarters}}{1\ \text{dollar}} = 2848\ \text{quarters}$$

(c) The trick is to put the unit that you want in the numerator. This way, the unit that you start with will cancel with the unit in the denominator. Let's try one with a little more chemistry to it. How much does half a mol of lithium weigh, in grams? We need the molar mass of Li, 6.941 g/mol. With it, the answer is:

$$0.5\ \text{mol Li} \times \frac{6.941\ \text{grams}}{1\ \text{mol Li}} = 3.4705\ \text{grams Li}$$

(d) The molar mass has the units of grams/moles, meaning that with the conversion principle, you can convert from moles to grams. By flipping the fraction, you can even use the molar mass to turn grams into moles. For example,

$$3.4705\ \text{grams Li} \times \frac{1\ \text{mol Li}}{6.941\ \text{grams}} = 0.5\ \text{mol Li}$$

(e) Here are some questions which use unit conversion:

i. What is the volume of 124 grams of neon, Ne, if the density of Ne is 0.901 g/L?

ii. If a runner can consistently keep a pace of 1.5 miles per 10 minutes, how far could he run in 43 minutes?

iii. Any good cookie needs 6 chocolate chips. If the cookies are packaged in 3's, how many packages of cookies will be made with 78 chocolate chips?

iv. If you have 1 mol of the previously mentioned perfect cookies, how many mols of chocolate chips were used?

v. How many moles of chlorine, Cl, are there in 0.75 moles of $MgCl_2$? How many chlorine atoms?

vi. A tea cup can hold 28 mL. How many grams of strong tea (with a density of 0.00105 kg/mL) can it hold if the cup is 86% full? (1 Liter = 1000 mL)

vii. There are 7 billion people on the Earth. And the average human lifespan is between 2 and 3 billion seconds. Let's say everyone on the Earth started a new project. Together, as a species, everyone would eat together one mole of M&Ms. To within a factor of two or three, how many M&Ms would everyone have to eat for *every* second for the remainder of their lives?

1.2 Chem 1070 workshop 2: Proportionality

1. **Proportionality**

   (a) Using efficiently proportionality is worth one notch in your Chem 2070 grade. Proportionality relations can be direct, indirect, or follow yet another mathematical relation.

   (b) For each of the pairs of quantities below, please determine if a proportionality relation exists. If a proportionality relation does exist:

      i. Write the equation relating the quantities. Choose sensible letters for each of the quantities (eg., p for pressure and V for volume). You will need to identify the proportionality constant, giving this constant an appropriate letter designation as well. Remember the letter describes the quantity, **not** the unit of the quantity.
      ii. If the proportionality relation is approximate use the $\approx$ symbol instead of the $=$ sign.
      iii. Next to each quantity and proportionality constant write its typical unit.
      iv. Write the proportionality relationship using the proportionality symbol $\propto$. Note constants do not appear in proportionality relations.

   To answer some of these questions you will need to know the ideal gas law: $pV = nRT$, where p is pressure, V volume, n the number of moles, R the gas constant, and T the absolute temperature. You will also need to know that specific heat, C, is the proportionality constant relating the energy put into a sample, E, and the rise in temperature, ΔT, of a given sample mass.

      i. The money spent at the gas pump and the amount of gas pumped.
      ii. Mass of a chemical sample of a molecule and the number of moles in the same sample.
      iii. The distance one travels on a US interstate and the amount of time one drives (assume one drives at a steady pace).
      iv. The temperature of an ideal gas and the volume of the same gas (assume the number of moles and the pressure are both constant).
      v. The temperature outside and the amount of clothes one wears.
      vi. The radius of a sphere and the volume of the same sphere.
      vii. The number of moles in a pure sample of a molecule, n, and the number of molecules in the same sample, N.
      viii. The volume of a liquid, V, and the mass of the liquid.
      ix. The temperature in Kelvin and the same temperature in degrees Celsius
      x. A person's height in feet and the same person's height in inches.
      xi. 1 L of solvent in which solute has been dissolved: the number of moles of solute and the volume of the solvent.
      xii. The amount paid in FICA (social security tax) and the amount you earn.
      xiii. The mass of a piece of iron and the volume of a piece of iron.
      xiv. The amount you eat and the amount you weigh.
      xv. The volume of water drunk and the number of molecules of water drunk.
      xvi. The amount you spend on education and the amount you earn.
      xvii. The amount of energy one heats a gram of compound and the temperature that the sample increases.
      xviii. The pressure of an ideal gas in a sealed flask and the the temperature of the gas.
      xix. A person's height and the same person's age.
      xx. The mass of a sample in kilograms and the mass of the same sample in pounds.
      xxi. The length of the side of a square and the area of the square.
      xxii. The specific heat of a substance and the mass of the substance.
      xxiii. The number of books on a bookshelf and the amount of available shelf space.
      xxiv. Consider the amount of the solute sodium dissolved in sea water. The Molarity of the solute and the number of moles of the solute.

xxv. Consider a 1 L sample of water in which varying amounts of NaCl have been dissolved. The Molarity of solute and the number of moles of solute.

(c) Proportionality relations can be used to answer the following questions. No calculators please.

i. An ideal gas with T and p constant: one doubles n, what happens to V?

ii. One heats a kg of iron metal tripling its energy: what happens to the specific heat?

iii. An ideal gas in a sealed flask: one halves the pressure, what happenes to the volume?

iv. One halves the number of molecules in a sample of solid iron: what happens to the volume of the sample?

v. A sealed sample of the gas molecule NO completely undergoes the reaction $2NO(g) \longrightarrow N_2O_2(g)$. What happens to the number of moles, n?

vi. An ideal gas: p triples, n doubles, and V halves, what happens to T?

vii. Upon cooling, a liquid becomes 5% more dense, what happens to the liquid volume?

viii. The density of water is 1 g/mL. How much does a L of water weigh?

ix. An ideal gas in a sealed balloon in a lab : one heats the balloon until temperature doubles:, what happens to p, n, and V?

x. One mole of any ideal gas at STP ie., $0^oC = 273$ K and 1 atm pressure, always occupies 22.4 L. (Please remember the volume 22.4 L) A sealed flask contains an ideal gas. The flask initially is at STP (O ^oC and 1 atm). The flask is 2240 L big. How many moles of gas does the flask contain?

xi. The amount of energy, in Joules, required to heat a gram of substance 1 degree Celsius is the specific heat in J/g^{o}C . The specific heat of water is 4.18 J/g^{o}C. How many Joules are required to heat 1 kg of water 10 degrees Celsius?

xii. A sealed flask contains an ideal gas. The flask initially is at STP (0 ^oC and 1 atm). The flask is 14.9 L big. The flask is heated until it reaches a pressure of 3 atm. What is its final temperature? (What is the relation between 22.4 and 14.9?)

xiii. The specific heat of brass is 1/10 that of water. To two significant figures, how much energy is required to heat 100 g of brass 20 ^oC?

xiv. A sealed flask contains an ideal gas. The flask initially is at 457° C (what's the approximate relation between 457 and 273?) and 20 atm. The flask is 14.9 L big. Should the flask be heated or cooled to reach a pressure of 5 atm?

xv. When one strikes a metal coin with a hammer, energy is transferred from the hammer to the coin. A gold coin struck by a hammer gets warm quicker than a copper coin. Does gold or copper have a higher specific heat?

xvi. Ethyl alcohol has a density 4/5 that of water. How much less does 3 L of ethyl alcohol weigh than 3 L of water?

1.3 Chem 1070 workshop 3: Reactions and combustion analysis

1. **Reactions**

   (a) In chemistry, it's a reaction if molecules come together to make new molecules very different from the old. In a chemist's mind a reaction is a bit like what happens when two bicycle wheels come together with a bicycle frame. Put the right way, the wheels and the frame transform into a bicycle, something which forms a new unit and whose properties and uses are vastly different from its former configuration.

   (b) Chemists might call a wheel Wh and a frame Fr (we love calling things by one or two letter abbreviations.) But please note a bicycle would not be called Bi, but rather $FrWh_2$. For in a chemist's mind much more important, than the object itself, are the parts from which it is derived. The transformation from wheels and frame to bicycle would be expressed as,

   $$1\,\mathrm{Fr} + 2\,\mathrm{Wh} \longrightarrow 1\,\mathrm{FrWh_2}.$$

   It is the long arrow in the above expression which represents the chemical reaction, the transformation by which new things are unexpectedly made from the old.

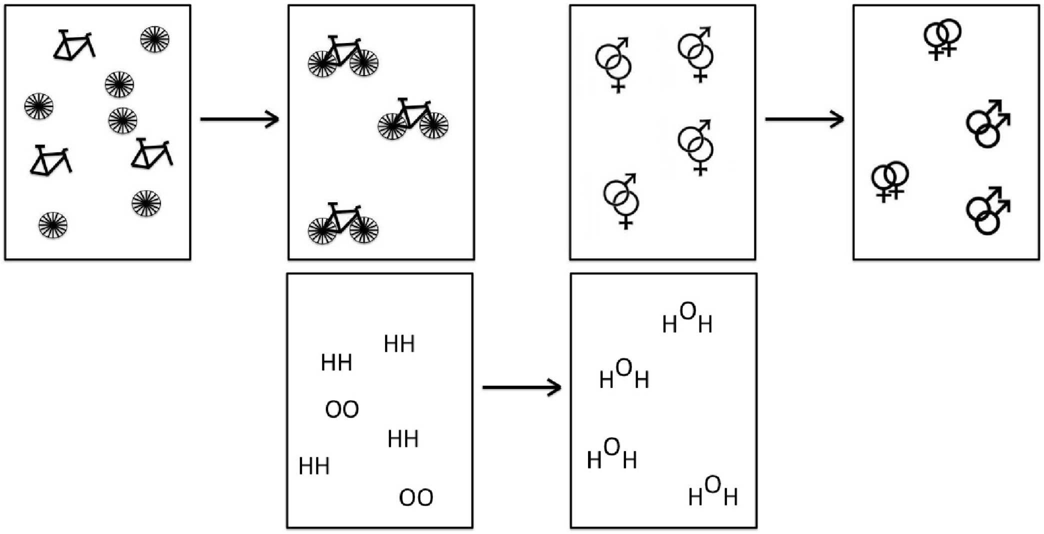

   (c) The pictures above describe the $1\,\mathrm{Fr} + 2\,\mathrm{Wh} \longrightarrow 1\,\mathrm{FrWh_2}$, as well as two more "reactions", one involving males and females, and the other one involving hydrogen and oxygen gas molecules coming together to make water, H_2O. Can you find chemical equations for each of these situations?

   (d) Perhaps in answering the last question you noticed that we could write the hydrogen plus oxygen to water reaction either as $H_2 + O_2 \longrightarrow H_2O$, telling ourselves *which* molecules made the new molecule, *or* $2\,H_2 + 1\,O_2 \longrightarrow 1\,H_2O$, further telling ourselves *how many* of each the old molecules are needed to make each new one.

   Chemists call the first kind of reaction an unbalanced and the second kind a balanced reaction. We find both formats useful. We find them both so useful that one of the very first problem types general chemistry students learn is translating the former unbalanced equations into the latter balanced ones.

   (e) The figure below teaches you a useful and general procedure of how to do so. It works in all but the most complicated situations. We'll teach you about these harder balancing problems when we discuss *oxidation-reduction* reactions in the third week of the course.

   (f) Using these procedure we could balance the following reactions,

      i. $Fe + HCl \longrightarrow H_2 + FeCl_2$

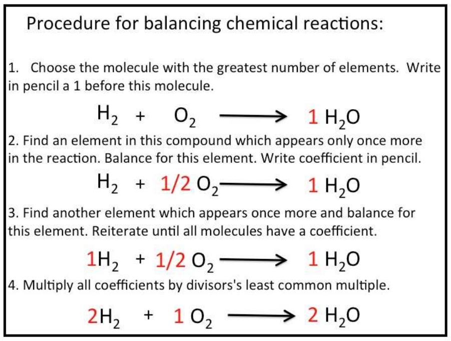

ii. $\mathrm{Na} + \mathrm{Cl} \longrightarrow \mathrm{NaCl}$

iii. $\mathrm{HBr} + \mathrm{Mg(OH)_2} \longrightarrow \mathrm{MgBr_2} + \mathrm{H_2O}$

iv. $\mathrm{C_2H_6} + \mathrm{O_2} \longrightarrow \mathrm{CO_2} + \mathrm{H_2O}$

v. $\mathrm{C_4H_{10}} + \mathrm{O_2} \longrightarrow \mathrm{CO_2} + \mathrm{H_2O}$.

2. **Combustion analysis**

(a) We present the combustion problem using the panels below. Please solve the question in the last panel.

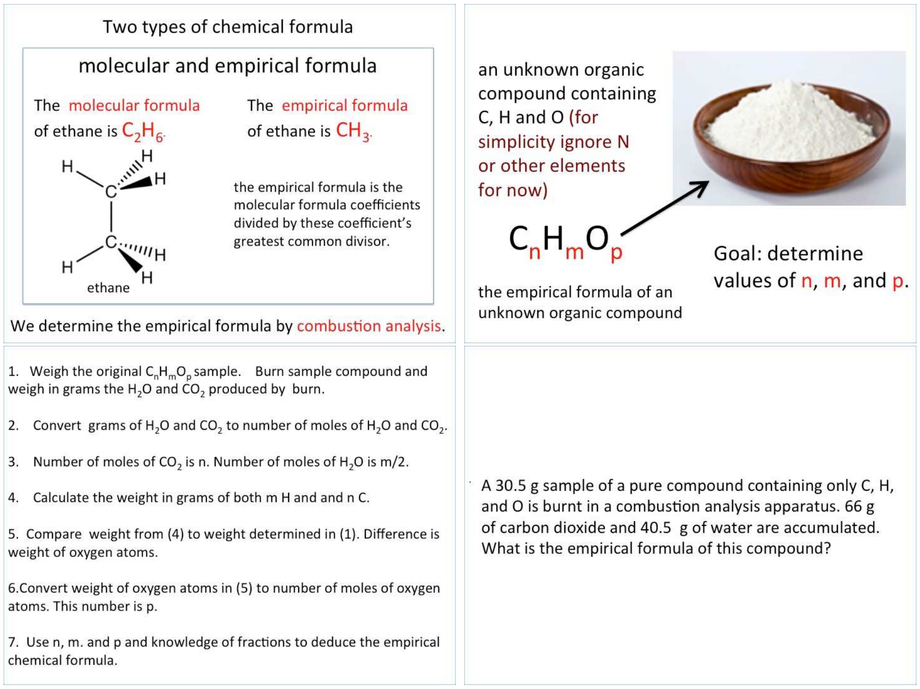

(b) The combustion problem here is an example of a whole class of problems. You should learn to recognize combustion problems when you see them. Combustion problems:

i. will pretty much always have a molecule whose chemical formula you don't know,

ii. and they will always have specified amounts of different product molecules.

3. **Problem taken from H. B. Fine's** ***College Algebra*****:** A and B run 440 yard races. In the first race A gives B a start of 20 yards and beats him by 2 seconds. In the second race A gives B a start of 4 seconds and beats him by 6 yards. How fast doea A run the 440?

1.4 Chem 1070 workshop 4: Limiting reagents

1. **Limiting reagents**

   (a) *Some helpful pictures*

   i. The pictures below help illustrate the concept of limiting reagents. The first picture shows that when only three frames are mixed with ten wheels, only three bicycles can be made. Note in this case, the number of frames, not the number of wheels, control the number of bicycles produced. In fact, as long as we had at least six wheels, we could have had any number of wheels; 9, 17, 71 wheels; and as long as we had three frames, we would still have made only three bicyclyes.

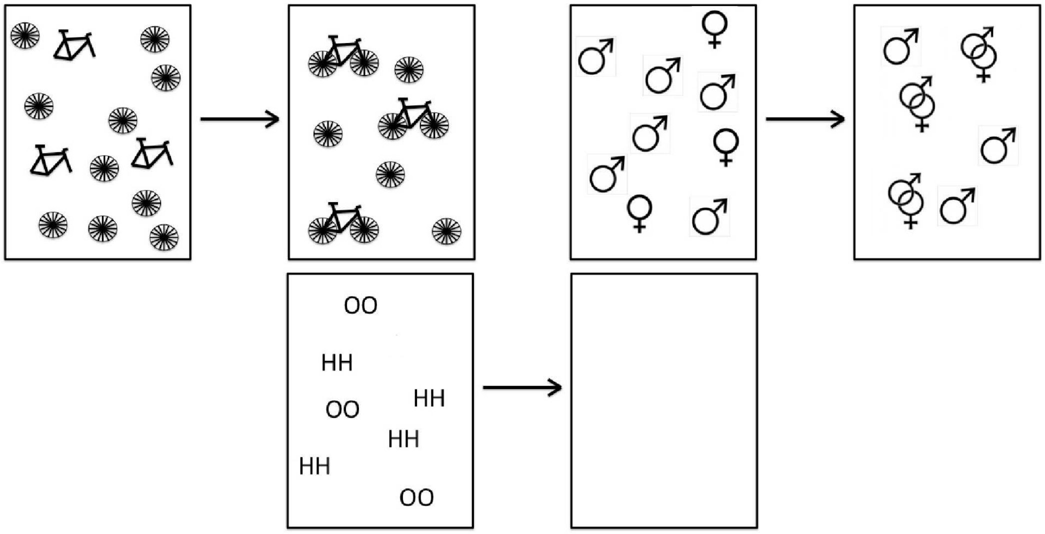

   ii. In this bicycle example, it was the number of frames which was the *limiting reagent*, not the number of wheels. In just the same way two more pictures are shown.

   A. What is the limiting reagent in the first of these pictures, the male-female picture?

   B. Turning to the last picture, can you correctly fill in the product in the empty H_2-O_2-H_2O box?
   I. How many H_2O molecules could be formed?
   II. Which reactant molecules would be left behind?
   III. What is the limiting reagent in this specific H_2O example?

   C. In the last problem, it was the H_2 molecules that were the limiting reagent, even though initially there were more of them (4 H_2 vs. 3 O_2). Why was this? See if you can put the balanced equation, $2\,H_2 + O_2 \longrightarrow 2\,H_2O$, into your explanation.

   (b) *Limiting reagents are all about the recipe.*

   i. Balanced reactions are recipes. And it is the recipe which determines the limiting reagent. Consider this not very good recipe for pizza: 1 dough (Do) + 2 packages of cheese (Ch) + 3 tomatoes (To) makes one pizza. The recipe can be written as

$$1\,\mathrm{Do} + 2\,\mathrm{Ch} + 3\,\mathrm{To} \longrightarrow \mathrm{Do_1Ch_2To_3}.$$

   (Note again the chemist's obsession with what elements an object comes from rather than a symbol like Pi for pizza representing the final product itself.)

   A. Consider the situation where you have 1 dozen Do + 1.5 dozen Ch + 4 dozen To. Please answer the following questions
   I. What is the maximum number of *dozens* of pizzas that can be made?
   II. What was the limiting reagent?

B. Now consider the following improbable situation. You have 2 moles Do + 5 moles Ch + 7 moles To.
I. What is the maximum number of *moles* of pizzas that can be made?
II. What was the limiting reagent?

ii. And now a chemical example:

$$2\,KMnO_4 + 5\,C_2H_2O_4 + 3\,H_2SO_4 \longrightarrow 2\,MnSO_4 + 10\,CO_2 + 8\,H_2O + K_2SO_4$$

A. With 15 moles of $C_2H_2O_4$, what is the maximum number of times the above reaction can be run?
B. With 0.60 moles of $KMnO_4$, what is the maximum number of times the above reaction can be run?
C. If there are 8 moles of $KMnO_4$, 20 moles of $C_2H_2O_4$, and 10 moles of H_2SO_4, what is the limiting reagent?
D. If there are 5.9 moles of $KMnO_4$, 10.6 moles of $C_2H_2O_4$, and 8.4 moles of H_2SO_4, what is the limiting reagent?
E. If there are an equal number of moles of $KMnO_4$, $C_2H_2O_4$, H_2SO_4, what is the limiting reagent?

(c) The *ICE* method for solving limiting reagent problems:

i. This method involves the making of a table with either four or five lines.
A. The first line of the table is the balanced chemical reaction.
B. The second line specifies (for Chem 2070 typically in moles, but in Chem 2080 typically in moles/liter) the *I*nitial amount of each compound.
C. The third line lists the *C*hange in the number of moles of a compound assuming maximal reaction.
D. The fourth line (often omitted) is the change in the number of moles of a compound if the reaction is only partially complete.
E. The fifth line specifies the *E*nd amount of compound. It is the sum of the *I* and *C* lines.

Important check: the numbers in your change line(s) should be in the same ratio to one another as the coefficients in the balanced reaction line.

(d) Please solve the following problems:

i. A student is capable of writing a term paper with 3 hours (Hr) and 2 cups of coffee (Cf). Write a balanced chemical equation. Remember a term paper does not get its own two letter symbol. If a student has 2921 minutes and 38 cups of coffee, which is the limiting reactant, time or coffee?

ii. There are 20 g C_2H_4 and 58 g O_2. How many grams of CO_2 are produced by the following reaction, if the following reaction goes to its maximal possible completion?

$$C_2H_4 + 3\,O_2 \longrightarrow 2\,CO_2 + 2\,H_2O$$

iii. Ca reacts with As to form Ca_3As_2. We start initially with 10.0 g of both of the two elements, The reaction proceeds to only 75.0% of maximal completion. How many grams are there of elemental As, elemental Ca, and calcium arsenide at the end of the reaction?

iv. Ca reacts with As to form Ca_3As_2 and with O_2 to form CaO. We start initially with 10.0 g of both Ca and As but only 3.0 g of O_2. Assuming that the maximal amount of CaO is produced and given this constraint the maximal amount of Ca_3As_2 is synthesized, how many g are there of all the possible compounds at reaction end?

2. **Problem taken from H. B. Fine's** ***College Algebra***

Two points move at constant rates along the circumference of a circle whose length is 150 feet. When they move in opposite senses they meet every 5 seconds; when they move in the same sense they come together every 25 seconds. What are their individual speeds?

1.5 Chem 1070 workshop 5: Calculator-free chem-math I

1. We do these problems without calculators (if you can, just write down the answer or say it aloud).

   (a) Consider the reaction

   $$4\,NH_3 + 3\,O_2 \longrightarrow 2\,N_2 + 6\,H_2O$$

   i. With 0.40 moles of NH_3, what is the maximum number of times the above reaction can be run?

   ii. If there are 8 moles of NH_3 and 8 moles of O_2, what is the limiting reagent?

   iii. If there are 8 moles of NH_3 and 8 moles of O_2, how many moles of H_2O were made

   iv. If there are 8.2×10^{-3} moles of NH_3 and 9.5×10^{-3} moles of O_2, what is the limiting reagent?

   v. If there are 11.2×10^{-5} moles of NH_3 and 6.5×10^{-5} moles of O_2, what is the limiting reagent?

   vi. If there are 8.2×10^{-7} moles of NH_3 and 9.5×10^{-5} moles of O_2, what is the limiting reagent?

   vii. If there are twice as many moles of O_2 than NH_3, what is the limiting reagent?

   (b) Convert as best you can to a fraction (whose numerator and denominator are both 1-digit integers):

   i. 1.50

   ii. 2.67

   iii. 1.33

   iv. 0.167

   v. 2.25

   vi. 1.80

   (c) Without a calculator please either convert the following gram or volume values into moles or mole values into grams:

   i. 24 g of ozone molecules, O_3

   ii. 24 g of oxygen molecules, O_2

   iii. 24 g of oxygen atoms

   iv. 1 L of water

   v. 4.4×10^5 g of carbon dioxide

   vi. 10.0 moles of $C_{10}H_{16}$

   (d) Please determine to one sig fig:

   i. $(2 \times 10^3)^2 + 2 \times 10^4 =$

   ii. $(2 \times 10^3)^2 \times 2 \times 10^4 =$

   iii. $(2 \times 10^3)^2 \div 2 \times 10^4 =$

   iv. $(2 \times 10^3)^{-2} - 8 \times 10^4 =$

   v. $4 \times 10^4 \div (2 \times 10^3)^2 =$

   (e) Which weighs more:

   i. 20 g CO_2 or 0.5 moles CO_2

   ii. 3/4 moles CO_2 or 25 g CO_2

   iii. a mole of N or a mole of N_2

   iv. 2 moles of water or 1 L of water

   v. 4 moles of CH_4 or 1 mole of C_4H_4

   vi. 6 moles of NaCl or 100 g NaCl

   (f) Please find either whole number or fractional answers for the following ratio questions.

   i. 8 is to 2 as what number is to 4?

   ii. 5 is to 2 as what number is to 7?

   iii. 4 fish are to 3 toucans as 8 fish are to what?

iv. 5 moles of C are to 3 moles of H as 15 moles of C are to what?

v. 3 moles of CO_2 are to 1 mole of H_2O as 1.8 moles of CO_2 is to what?

(g) Please convert to the simplest accurate empirical formula. Allow for rounding and experimental errors.

i. $Ge_{1.32}As_{1.00}$

ii. $Fe_3C_{12}O_6$

iii. $C_{1.65}H_{2.32}O_{1.00}$

iv. $C_{1.167}Si_{1.335}H_{1.000}$

(h) Please convert:

i. 70 nm = ? μm

ii. 20 μL = ? nL

iii. 1.2×10^3 nm = ? μm

iv. 1 cm^3 = ? m^3

(i) Please state what p equals. As p stands for pressure it is always a number bigger or equal to zero.

i. $\frac{7T}{p} = \frac{14}{5}$

ii. $p^2 - 4 = 0$

iii. $\frac{2.4\times10^{-3}}{p} = 4.8 \times 10^{-2}$

(j) Please find the following averages:

i. A group of students is 3/4 right-handed amd 1/4 left-handed. Strangely the right-handed people are all 5 feet tall while the left handed people are 6 feet tall. What is the average height of a person in this group? Remember, no calculator should be used.

ii. 99% of carbon atoms are ^{12}C, almost all the remaining are ^{13}C. To four significant figures, what is carbon's atomic weight?

iii. 93% of lithium atoms are ^{7}Li, almost all the remaining are ^{6}Li. To three significant figures, what is lithium's atomic weight?

iv. 76% of chlorine atoms are ^{35}Cl, almost all the remaining are ^{37}Cl. To three significant figures, what is chlorine's atomic weight?

1.6 Chem 1070 workshop 6: The stoichiometry diagram and stoichiometry problem types

1. **The stoichiometry diagram**

   The stoichiometry diagram is used to illustrate gram, molarity, and molar relations, the key relations which underlie complex stoichiometry problems.

   Stoichiometry diagrams consist of the following parts:

   (a) **Circles** are placed around all compound **amounts** in the problems (and/or elements which are parts of a compound). Information about the compound, e.g., its mass, number of moles, volume, etc. are placed inside the circles. Mixtures of two compounds can be represented as overlapping circles.

   (b) **Lines** are placed between compound/element amounts stating the exact gram, molarity, or molar relation between the circled compounds or elements. *Lines should state molar, molarity, or gram relationships; they are not reaction pathways.*

   (c) **Balanced chemical reactions** are represented as molar lines with the mole ratios, based on the balanced chemical reaction, written on the lines.

   (d) **Empirical and molecular formulas** represent elemental molar ratios and therefore can also be represented as molar lines with the atomic mole ratios written on the lines.

   (e) **Limiting reagent** problems are represented with appropriately boxed ICE tables. (*The entries in the change line of the ICE table corresponds exactly to a balanced chemical reaction molar line.* This is so as the entries in the ICE table change line are always in the same ratio as the coefficients of the balanced chemical reaction.)

   (f) **Titrations** *are always* represented by horizontal molar lines. We note that a titration is the exact converse of the limiting reagent problem: in a titration problem, unlike a limiting reagent problem, all reactants are exactly used up by the reaction end. *Going further, while we generally do not use ICE tables for titration experiments, the numbers of a titration problem can always be thought of as the change line of an ICE table.*

   (g) **Multiple proportion** problems are typically best handled with gram and/or mole tables. An algebraic variable n will often need to be introduced to the molar table as typically we do not know the exact numerical value of moles for any single element.

2. **Stoichiometry problem types**

   We consider five categories of stoichiometry problems:

   (a) limiting reagent

   (b) combustion analysis

   (c) titration

   (d) chemical proportions

   (e) algebraic

   Please state the category of each of the following problems. Please then solve problems *without a calculator.*

   (a) 8.0 grams of sulfur and 9.5 grams of element Z both react completely to yield the compound SZ_2. Identify element Z. (simplified numbers but based on a 2070 prelim question)

   (b) NaOH is a base. It reacts with the acid HCl to form table salt dissolved in water. How many grams of HCl are required to exactly neutralize 80. g of NaOH?

   (c) 17 g of H_2S react to the maximal extent possible with 24 g of O_2 to make water and sulfur. How many moles of O_2, H_2S, and S are present at the reaction end?

(d) An unknown compound which contains no fluorine is placed in an excess of fluorine gas. 22 g of CF_4 and 26 g of CH_2F_2 are produced. What might this compound be?

(e) At STP a 44.8 L flask contains an equal molar mixture of H_2 and O_2. These molecules react forming water to the maximum extent possible. The flask remains at 273 K. What is the final pressure of the flask? Assume water vapor pressure is 0. (*Note that at STP standard temperature, 273 K, and standard pressure, 1 atm), 1 mole of an ideal gas always occupies 22.4 L.*)

(f) HCl is a gas. At STP, how many L of HCl gas are needed to neutralize 148 g of calcium hydroxide?

(g) A chemist completely reacts 25 grams of arsenic(a shiny gray solid) with excess bromine (a brown oily liquid). At the end of the reaction, she isolates 105 grams of pale yellow crystals. What is the empirical formula of the compound?(simplified numbers but based on a 2070 prelim question)

(h) 3.0 L of 0.5 M $KMnO_4$ reacts to maximal completion with 14 g of C_2H_4 in an excess of sulfuric acid according to the reaction:

$$6\,KMnO_4 + 5\,C_2H_4 + 4\,H_2SO_4 \longrightarrow 6\,MnSO_4 + 10\,HCO_2H + 3\,K_2SO_4 + 4\,H_2O$$

How many grams of C_2H_4 remain at reaction end? How many moles of HCO_2H were produced?

(i) A 150 g amalgam of Ru and Hg is oxidized to 174 g of RuO_2 and HgO. What was the original mass percent Ru?

(j) Hydrocarbons contain only carbon and hydrogen atoms. An unknown gaseous hydrocarbon is, by weight, 5/6 carbon. At STP, 13 g of the gas occupy 11 L. What is the molecular formula of the compound?

(k) The elements Q and R form two compounds. The first is Q_2R_3. What is the empirical formula of the second?

Compound	Mass of X	Mass of Y
Q_2R_3	24 g	24 g
2nd cmpd.	8 g	12 g

(l) A chemist has 100 mL of 4.00 M HBr. What exactly needs to be done in order to turn this entire solution into 3.00 M HBr?

(m) An unknown pure hydrocarbon is completely burned in excess oxygen to make 4.40 g of CO_2 and 1.800 g of H_2O. A gaseous sample of this gas is mixed with an equal number of moles of N_2. The N_2 gas proves to effuse twice as fast through a small hole. What is the molecular formula of this unknown hydrocarbon? (*Note that this problem requires prior knowledge of effusion.*)

1.7 Chem 1070 workshop 7: Core stoichiometry problems

1. An essential gen-chem skill is the ability to identify and solve the four core stoichiometry problem types: (1) combustion analysis, (2) limiting reagents, (3) titrations, and (4) chemical proportions. The questions below, where cited, are adapted from current gen-chem textbooks. Please identify the problem types and solve. *Questions based on the cited textbooks below have been modified, to reflect the reaction chemistry, inorganic nomenclature, and redox properties which you are expected to have already mastered.*

   (a) When 18.0 g of iron nitrate are mixed with a solution containing 50.0 g of sodium hydroxide, a dark precipitate forms. Determine the mass of the precipitate formed if the reaction goes to completion. (McQuarrie, *General Chemistry*)

   (b) 100.0 kg of an impure copper ore are recovered from a potential mining site. All the copper in the ore is in the Cu(I) state. Only the copper can be oxidized in the presence of KIO_3 following the equation (under acidic conditions)

$$Cu^{1+} + KIO_3 \longrightarrow Cu^{2+} + KI.$$

   8.86 L of 0.50 M KIO_3 are required to exactly needed to react with the Cu(I) in the impure ore. Calculate the mass percent copper in the mining sample.

   (c) The element X forms the compound $XOCl_2$ containing 59.6% Cl. What is element X? (Petrucci, *General Chemistry*)

   (d) Hydrogen gas is passed over solid iron(III) oxide. Water vapor is formed together with a black residue - a compound consisting of 72.3% iron and no hydrogen. Write a balanced equation for the reaction.(Petrucci, *General Chemistry*)

   (e) The amount of I_3^- (aq) in a solution can be determined by reaction with a second solution containing a known concentration of thiosulfate ion, $S_2O_3^-$ (aq). The determination is based on the reaction

$$I_3^- \text{ (aq)} + S_2O_3^- \text{ (aq)} \longrightarrow I^- \text{ (aq)} + S_4O_6^{2-}.$$

   Given that it requires 36.4 mL of 0.330 M $Na_2S_2O_3$ (aq) to exactly react with 15.0 mL of a KI_3 solution, what is the KI_3 molarity? (McQuarrie, *General Chemistry*)

   (f) Pyridine is recovered from coke-oven gases and is extensively used in the chemical industry, in particular in the synthesis of vitamins and drugs. Pyridine contains carbon, hydrogen, and nitrogen. A 0.5460 g sample was burned to produce 1.518 g of CO_2 and 0.311 g H_2O. Determine the empirical formula of pyridine. (McQuarrie, *General Chemistry*)

   (g) A compound X is placed in a box with oxygen and then combusted (see schema below left). What was the empirical formula of X? If the sample X originally consisted of two molecules, what was the compound's molecular formula?

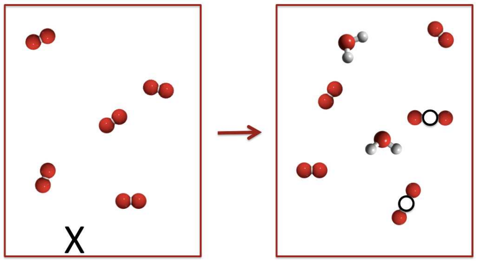

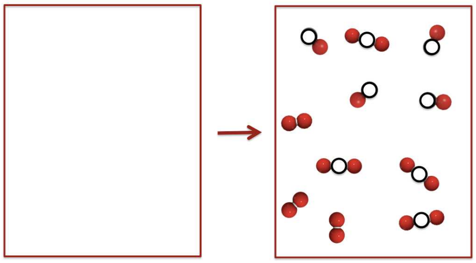

   (h) The reaction $CO(g) + O_2(g) \longrightarrow CO_2(g)$ (written in unbalanced form) has taken place, the outcome of which is shown above, right. Assuming there was no CO_2 in the reaction container before reaction, draw the original content of the reaction vessel. What was the percent yield of the reaction?

(i) A small piece of zinc is dissolved in 50.00 mL of 1.035 M HCl. At the conclusion of the reaction, the concentration of the 50.00 mL sample is redetermined and found to be 0.812 M HCl. What must the mass of the piece of zinc have been? (Petrucci, *General Chemistry*)

(j) An ore is to be analyzed for its iron content. A 4.24 g sample of the ore is dissolved in hydrochloric acid and passed over a reducing agent so that all the iron is in the form Fe^{2+} (aq). The Fe^{2+} is completely oxidized by 31.6 mL of a 0.0512 M of $KMnO_4$ (aq). The reaction can be espressed as:

$$KMnO_4\,(aq) + HCl\,(aq) + FeCl_2\,(aq) \longrightarrow MnCl_2\,(aq) + FeCl_3\,(aq) + H_2O\,(l) + KCl\,(aq).$$

Calculate the iron mass percentage of in the original ore. (McQuarrie, *General Chemistry*)

(k) Two compounds of Cl and X are found to have molecular masses and mass percents of chlorine of respectively 137 g/mol with 77.5% Cl and 208 g/mol with 85.1% Cl. What is element X? (Petrucci, *General Chemistry*)

(l) Silver nitrate is expensive. For a particular experiment, you need 100.0 mL of 0.0750 M $AgNO_3$ (aq), but only 60 mL of 0.0500 M $AgNO_3$ is available. You decide to pipet exactly 50.00 mL of the solution into a 100.0 mL flask, add an appropriate mass of $AgNO_3$, and then dilute the resulting solution to exactly 100.0 mL. What mass of silver nitrate must you use? (Petrucci, *General Chemistry*)

(m) An unknown carbon-hydrogen-nitrogen compound yields 0.458 g CO_2 and 0.374 g H_2O. A 48.6 g sample of the compound has 22.6 g of nitrogen. What is the compound's empirical formula? (Petrucci, *General Chemistry*)

(n) When calcium carbonate is added to a hydrochloric acid solution, a gas is produced. Write a balanced chemical equation for this reaction. If we started with 25.0 g of calcium carbonate and 15.0 g of hydrochloric acid, how many grams of gas would have been produced? (McQuarrie, *General Chemistry*)

1.8 Chem 1070 workshop 8: A-level stoichiometry problems

1. **Numerical stoichiometry diagram problems**

   Gen-chem textbooks are chock-full of stoichiometry problems all of which can pretty much be solved by the diagrammatic procedure outlined in the previous workshop. The goal of this worksheet is to give the student more challenging versions of these questions, A-level questions, beyond the level of most current textbooks. Please solve:

   (a) Prof. La Cheville-Tordue needs your help. She wishes to make a limiting reagent examination problem involving the **unbalanced** reaction:

   $$C_2H_6 + O_2 \longrightarrow CO_2 + H_2O.$$

   But the professor is in a rush to go to a scientific conference in Lausanne, Switzerland and asks you to write the problem in her stead.

   She asks that you write a limiting reagent problem where the following two things are true:

   i. At reaction end (the reaction having gone to maximal completion) the ratio of moles of O_2 and CO_2 is exactly 1:1.

   ii. Before the reaction started, there were exactly 10.0 g of C_2H_6.

   In setting up this problem, what must you choose as the initial amount of O_2 in the sealed flask?

   (b) A 0.578 g sample of pure tin is treated with gaseous fluorine until the weight of the resulting compound is constant at a value of 0.944 g. What is the empirical formula of the tin fluoride formed? (adapted from Mahan *University Chemistry*)

   (c) An aqueous solution of NaH_2PO_4 is treated with ammonium and magnesium ions to precipitate $MgNH_4PO_4{\cdot}6H_2O$. This is heated and decomposed to make $Mg_2P_2O_7$. This final reaction occured as:

   $$2\,MgNH_4PO_4 \cdot 6H_2O \longrightarrow Mg_2P_2O_7 + 2\,NH_3 + 13\,H_2O.$$

   1.054 g of $Mg_2P_2O_7$ were obtained. What is the miniumum amount of NaH_2PO_4 in solution? (adapted from Mahan *University Chemistry*)

   (d) Chromium, Cr, is known to make two compounds with the naturally occuring element, Z: Cr_2Z_3 and CrZ_2. When 54.232 g of one of these two compounds is heated, the compound loses Z (as a gas) and turns into 46.744 g of the other of the two compounds. No Cr is lost in this reaction. Which element in the periodic table is Z?

   (e) Iodine, I_2, and fluorine, F_2, react to form IF_3 very quickly at 100° C. This reaction is irreversible, ie., once the reaction product is made, the products will not reconvert back into the reactants.

   The reaction between iodine and chlorine, Cl_2, at 100^oC to produce ICl is much much slower, so much slower that we can assume that this latter reaction does not even start until the former reaction is entirely finished.

   If a sealed flask starts with 100.0 g of iodine, 20.0 g of fluorine, and 40.0 g of chlorine, and assuming all the above statements are true what is the theoretical maximal yield of ICl at 100° C?

   (f) Z is a naturally occurring element. 1.50 g of ZBr_2 is heated in a stream of chlorine gas and is completely converted to 1.05 g of ZCl_2 Which element in the periodic table is Z? (adapted from Mahan *University Chemistry*)

   (g) A 2.07 g sample of pure lead is dissolved in nitric acid to give a lead nitrate solution. This solution is treated with hydrochloric acid, chlorine gas, and ammonium chloride. The result is a precipitate of ammonium hexachloroplumbate, $(NH_4)_2PbCl_6$. What is the maximum weight of this product that could have been obtained from the lead sample? (adapted from Mahan *University Chemistry*).

   (h) Three new and previously unidentified elements have been discovered, elements X, Y, and Z. Elements X and Y are known to form a compound containing no Z, with chemical formula X_2Y_3. Elements Y and Z are known to make a second compound, containing no X, with chemical formula

YZ_2. Finally, all three elements are known to make a third compound, compound **Q**, of unknown stoichiometry. Compound **Q** is only composed of elements X, Y, and Z. Based on the data below, what is the empirical formula of compound **Q**?

Compound	Mass of X	Mass of Y	Mass of Z
X_2Y_3	58.40 g	115.39 g	—
YZ_2	—	86.40 g	40.38 g
**Q**	47.11 g	186.17 g	72.51 g

(i) Carbon is known to make two compounds with the element A: A_3C_2 and A_5C_3. When a 31.4461 g chunk of one of these two compounds is heated, graphite (carbon) is exuded, which is then gently scraped off the chunk. Experimentation shows that under these conditions the chunk has transformed into 30.9954 g of the other of the two compounds. No element A is lost nor has any gas been emitted in these processes. A is which element in the periodic table?

(j) A 4.07 g sample of zinc and magnesium is reacted completely with hydrochloric acid. If 0.204 g of hydrogen gas are produced by the reaction, calculate the mass percentage of zinc in the sample. *This stoichiometry diagram will require the use of one or two well-chosen algebraic variables.*

(k) Q and R are two naturally occuring elements. 123.98 g of QF reacts with 23.11 g of R_2Se to form 90.23 g of Q_2Se and 56.86 g of R_2QF_3, with no other compounds formed and no reactants remaining at reaction end. What is the element Q?

(l) The American food chemist James Currie discovered that the black mold *Aspergillus niger* could be used in the industrial synthesis of citric acid, $C_6H_8O_7$. In this procedure the mold is fed a diet (simplified for this problem) solely of sucrose ($C_{12}H_{22}O_{11}$), potasium nitrate, sodium phosphate, oxygen, and water. After appropriate feeding time, the mold is filtered out of the resulting solution, citric acid is isolated by precipitating it with calcium hydroxide to yield calcium citrate, from which citric acid is regenerated by treatment with sulfuric acid. These latter reactions can be expressed in unbalanced form as:

$$Ca(OH)_2 + C_6H_8O_7 \longrightarrow Ca_3(C_6H_5O_7)_2 + H_2O$$

$$H_2SO_4 + Ca_3(C_6H_5O_7)_2 \longrightarrow C_6H_8O_7 + CaSO_4.$$

With the current world production of citric acid at 1.6×10^9 kg/year, what is the best case scenario for the mass of sucrose required to produce this citric acid by the above process?

(m) 25.2 mL of a solution which is both 0.144 M KIO_3 and 6.00 M HCl is combined with 46.4 mL of a solution which is both 0.213 M $KHSO_3$ solution and 6.00 M HCl. Two irreversible **balanced** chemical reactions can occur, both shown below. The first of the two reactions proceeds much more quickly than the second reaction, so much quicker that you can assume **the first reaction is maximally completed BEFORE the second reaction even starts**. Both reactions proceed eventually to maximal completion. What is the maximum number of grams of I_2 gas which can be produced?

$$IO_3^-(aq) + 3\ HSO_3^-(aq) \longrightarrow I^-(aq) + 3\ HSO_4^-(aq).$$

$$IO_3^-(aq) + 5\ I^- + 6\ H^+(aq) \longrightarrow 3\ I_2(g) + 3\ H_2O(aq).$$

(n) Upon heating, 120.34 g of a mixed $AlCl_3$ and $CaCl_2$ sample is reduced into 33.456 g mixture of pure Al(s) and Ca(s), neither of which contains any chlorine. What was the molar ratio of the $AlCl_3$ to the total number of moles of compound in the initial chloride-containing sample?

1.9 1070 workshop 9: The cathode-ray and oil-drop experiments and mass spectrometry

1. This worksheet provides the student with the grounding in physics needed to understand the Thomson cathode ray experiment, the Millikan oil drop experiment, and mass spectrometry.

   (a) Forces have direction. Velocity, $\vec{v}$, and acceleration, $\vec{a}$, like forces, have direction. Forces and acceleration come together in Newton's second law, which states that the sum of all forces acting on an object equals the mass of the object multiplied by the acceleration of this same object:

   $$\sum \vec{F}_i = m\vec{a}.$$

   Individual forces acting on the object are $\vec{F}_1$, $\vec{F}_2$, and so forth. Key individual forces are listed below.

   (b) In the case of the classic experiments (Thomson's cathode-ray experiments, Millikan's oil-drop experiment, and mass spectrometry), three individual forces are needed:

   $$\vec{F}_{electrostatic} = q\vec{E}$$

   $$F_{electrostatic} = k_C \frac{q_{other} q}{r^2} \quad \text{(in the case of just one } \textit{other} \text{ charged particle)}$$

   $$\vec{F}_{magnetic} = q\vec{v} \times \vec{B}$$

   $$\vec{F}_{gravitational} = m\vec{g},$$

   where $\vec{E}$, $\vec{B}$, and $\vec{g}$ are respectively the electric field, the magnetic field, and the Earth's gravity; q is the charge of the object; and m is its mass. The electric field points from the positively charged plate to the negatively charged plate, the magnetic field points from the magnet's North Pole to its South Pole, and the Earth's gravity is pointed downwards.

   (c) Multiplicative negative numbers in the above expressions, reverse the direction.

   (d) The magnetic force requires use of the right-hand rule. The thumb, forefinger, and middle finger are each pointed into mutually perpendicular directions. For a moving positively charge particle, the right-hand thumb points in the direction of the velocity of the particle, the right-hand forefinger points in the direction of the magnetic field and the right-hand middle finger indicates the direction of the magnetic force. Conversely, for a moving negatively charge particle, the left-hand thumb points along the direction of the particle's velocity, the left-hand forefinger points in the direction of the magnetic field, and the left-hand middle finger indicates the direction of the magnetic force.

2. The questions below test the student's ability to use the above facts in the understanding of these three experiments.

   (a) **Mass spectrometry**

   i. Which of the three forces play a role in mass spectrometry?

   ii. In mass spectrometry, the masses being observed are typically positively charged. If the observed masses are deflected downwards, is the positive charged plate above or below the particle beam? Use an equation to justify your answer.

   iii. Use Newton's second law to show the role of mass in the angle of beam deflection.

   iv. Can the direction of deflection determine the sign of the charge of the particles within the beam? Use an equation to justify your answer.

   v. If the charges of the particle in the beam are increased, does this increase or decrease the angle of deflection? Use an equation to justify your answer.

   (b) **The cathode-ray experiment**

   i. Which of the three forces play a role in Thomson's cathode-ray experiment?

ii. If the cathode ray is deflected downwards by the electric field, is the positive charged plate above or below the particle beam? Use an equation to justify your answer.
iii. If the cathode ray is deflected downwards by the magnetic field, is the North Pole *either* above or below the particle beam? Use an equation to justify your answer.
iv. Can the direction of deflection by the electric field determine the sign of the charges in the cathode ray? Use an equation to justify your answer.
v. Can the direction of deflection by the magnetic field determine the sign of the charges in the cathode ray? Use an equation to justify your answer.
vi. Derive a proportionality relation for the angle of deflection in the electric field using q_e/m_e and E.
vii. Derive a proportionality relation for the angle of deflection in the magnetic field using q_e/m_e and B. Ignore the role of velocity.
viii. Did the Thomson experiment lead to the determination of q_e, m_e, or q_e/m_e?

(c) **The oil-drop experiment**

i. Which of the three forces play a role in Millikan's oil drop experiment?
ii. In the Millikan oil drop experiment, what was used to charge the oil drops?
iii. Was the upper plate in the oil drop experiment positively or negatively charged? Explain.
iv. Did the oil drop experiment determine the sign of the charge of the oil drops. Explain.
v. In the Millikan oil drop experiment the oil drops were suspended. What equation does this suspension lead to?
vi. In the Millikan oil drop experiment, does one directly determine q_e or Nq_e, where N are the integers 1,2,3, and so forth? Explain.
vii. Did the Millikan oil drop experiment by itself lead to the determination of q_e, m_e, or q_e/m_e? Explain.
viii. Did the Millikan oil drop experiment together with the Thomson cathode ray experiment lead to the determination of q_e, m_e, or q_e/m_e? Explain.

1.10 Chem 1070 workshop 10: Electronegativity, oxidation states, and reduction states

1. **Electronegativity:** Electronegativity measures the degree to which elements are able to keep and attract valence electrons. High electronegativity means a high ability to both keep and attract electrons.

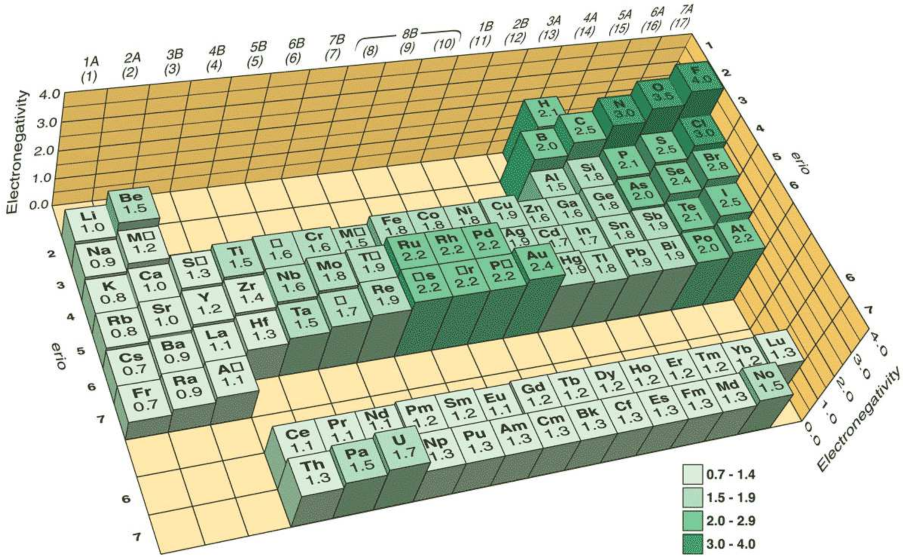

   (a) Two statements worth memorizing:

      i. The closer a main group element (columns 1-2 and 13-17) is to fluorine the more electronegative it usually is.

      ii. The closer a transition or noble metal element (columns 3-12) is to gold the more electronegative it usually is.

   (b) Without looking up the electronegativity values of these elements, please rank from highest to lowest electronegativity:

      i. Cl, Ca, Cs

      ii. Ru, Sc, Nb

      iii. Zn, Cd, Hg

      iv. Se, Te, Po

2. **Redox** (**Red**uction-**ox**idation: Reduction is the *gaining* of electrons. Oxidation is the *loss* of electrons. Electronegative elements tend to become reduced, electropositive elements become oxidized. *Noble gas electron configurations* are often particularily stable.

   (a) *Shortcut method of determining oxidation states*: Wherever possible, the most electronegative element reaches its normal negative oxidation state. Wherever possible, the most electropositive element reaches its normal positive oxidation state. Please find the *oxidation states for all atoms* in the molecules and ions below.

      i. SO_4^{2-}, the sulfate ion.

      ii. SO_2Cl_2

      iii. CH_4

      iv. elemental Ge

      v. PO_4^{3-}, the phosphate ion.

      vi. $P_2O_7^{4-}$

vii. C_2H_5OH
viii. HOOH, hydrogen peroxide
ix. FOOF
x. BH_3
xi. NO_3^-, the nitrate ion.
xii. HCl, hydrochloric acid.
xiii. MnO_4^-, the permanganate ion.
xiv. XeF_5^+
xv. HCOOH
xvi. KrO_2F^+
xvii. AsF_3
xviii. SiF_6^{2-}
xix. $ClOF_2^+$
xx. I_2
xxi. NaOCl, one of main components of bleach
xxii. $Cr(OH)_4^-$
xxiii. SO_2F_2

1.11 Chem 1070 workshop 11: Balancing redox reactions and compound names

1. **Balancing redox reactions:** The procedure given below correctly balances all redox reactions in aqueous solution which you will see in Chem 2070/2080.

   (a) *Establishing the two half reactions:*

      i. Find an atom, whose location in a product **and** a reactant compound/ion is self-evident and whose oxidation state undergoes a change during the reaction. Place the compounds/ions containing this atom into the beginning of your first half reaction.

      ii. Balance your first half reaction for all elements other than oxygen or hydrogen. Do not, at this point, balance for charge.

      iii. Choose all compounds/ions not contained in your first half-reaction as a start for your second half-reaction.

      iv. Balance your second half reaction for all elements other than oxygen or hydrogen. Do not, at this point, balance for charge.

   (b) *Correct order for final balancing of half-reactions:*

      i. Balance both half reactions for O using the H_2O molecule.

      ii. Balance both half reactions for H using the H^+ ion.

      iii. Balance both half reactions for charge using electrons, e^-.

   (c) *Combining the half reactions:*

      i. Multiply each half reaction by a whole number so that both half-reactions have the same total number of electrons.

      ii. Sum the two half reactions from this last step so as to make a single whole balanced reaction. This summed reaction is the balanced reaction **in acidic aqueous solution**.

      iii. If the reaction is carried out in base, add to both sides hydroxide ions, OH^- ions , equal to the number of H^+ ions. In doing so, you have converted all the H^+ ions on one side of the reaction into H_2O molecules. The other side of the reaction has now been loaded with the correct number of OH^- ions. The reaction with no remaining H^+ ions is the balanced redox reaction **in basic aqueous solution**.

   Please balance the following equations. Unless otherwise told, please assume acidic conditions.

   (a) $Mn^{2+}(aq) + ClO_3^-(aq) \longrightarrow MnO_2(s) + ClO_2(aq)$

   (b) $Mn^{2+}(aq) + H_2O_2(aq) \longrightarrow MnO_2(s)$; basic conditions

   (c) $MnO_4^-(aq) + CH_3OH(aq) \longrightarrow Mn^{2+}(aq) + HCOOH(aq)$

   (d) $S_8 + O_2 \longrightarrow SO_4^{2-}(aq)$

   (e) $OCl^-(aq) + Cr(OH)_4^-(aq) \longrightarrow CrO_4^{2-}(aq) + Cl^-(aq)$; basic conditions

   (f) $PbO_2(s) + Cl^-(aq) \longrightarrow PbCl_2(s) + O_2(g)$

   (g) $Fe(OH)_2(s) + O_2(g) \longrightarrow Fe(OH)_3(s)$; basic conditions

   (h) $CuSCN + KIO_3 + HCl \longrightarrow CuSO_4 + KCl + HCN + ICl$

2. **Inorganic compound names:** Prof. Lancaster (see also chapter 10 of the textbook) has introduced you to common elemental oxidation states and the names of common polyatomic ions. (*Note: The worksheet is referring to Prof. Lancaster, one of the instructors of the fall 2016 main gen-chem course.*) Please use this information to deduce the chemical formulas for the following compounds:

   (a) sodium hypochlorite

   (b) aluminium nitrate

   (c) iodic acid

(d) magnesium bicarbonate

(e) gallium oxide

(f) ammonium sulfite

(g) barium nitrite

(h) nitrous acid

(i) lithium thiocyanate

(j) cesium perbromate

1.12 Chem 1070 workshop 12: Classifying reactions

1. The reaction classification scheme given in this worksheet replaces the one given in the textbook: it is more in line with the thinking of inorganic chemistry professors such as Kyle Lancaster and myself. Mastering this reaction classification scheme allows the student to predict whether a reaction can or can not occur.

2. **The 1070 inorganic reaction classification**:

   (a) Four reaction types: reaction type identified in the following **fixed order**. Once a reaction type has been identified, subsequent reaction types are not considered.

      i. First, **redox reactions**: Comb the elements in the reaction for those which either reduce or oxidize. If you locate an element which reduces/oxidizes, there will be another element which oxidizes/reduces. Consider the relative electronegativities of this pair of reduced/oxidized elements. At STP, redox reaction favorability is determined by these relative electrongativies: the more electronegative atom reduces; the more electropositive oxidizes.
      At higher temperatures, there is an additional complication (which, as is discussed in Chem 2080, is based on the exothermicity/endothermicity of the reaction). For now, just note that if a redox reaction (i) does occur and (ii) does not follow the course predicted by electronegativity, it is because the reaction is run at high temperature. Note the converse statement is not true: high temperature reactions can follow electronegativities: they just don't always do so.
      In the exercises below: State, in writing, the oxidation states before-and-after of the pair of oxidized/reduced elements. State if, based on electronegativity, the reaction is favorable or not. Finally, if not favored by electronegativity, but the redox reaction nevertheless does occur, note that the reaction takes place at higher temperature.

      ii. Second, **acid-base reactions**: Chem 2070/2080 acid-base reactions always involve proton (H^+) transfer. Amongst the reactants identify the compound/ion acting as an acid and the one acting as a base. Find the same amongst the products. If the product acids and bases are conjugates, identify them as such. Acid-base reactions always favor the stronger acid and base as reactants and the weaker acid and the weaker base as products. In order to determine the direction of reaction you therefore must always compare *either* the strength of the reactant acid with that of product acid *or* the reactant base with that of the product base.
      State, in writing, the reactant acid, the reactant base, the product acid, and the product base. If there are any conjugates, identify their conjugate nature. Based on your knowledge of acid/base strengths, decide, in writing, if the reaction is favorable. In just a couple of words, state the source of information used to identify the relative acid/base strengths.

      iii. Third, **precipitation/dissolution reactions**: *State briefly the solubility rule which supports or does not support the given reaction.*

      iv. Fourth **insertion/elimination reactions**. Insertion/elimination reactions are the "irregular verbs" of reaction types: they must be memorized individually. *Identify, in writing, the inserted or eliminated species. Memorize the reaction.*

   (b) Shown on the next page are 20 different reactions taken from Chapter 10 of your textbook. Based on the above classification scheme. Please carry out the instructions given above for each of the twenty reactions.

      i. H_3O^+ (aq) + OH^- (aq) $\longrightarrow$ 2 H_2O (l)
      ii. Na (s) + S (s) $\longrightarrow$ Na_2S (s)
      iii. H_2CO_3 (aq) $\longrightarrow$ H_2O (l) + CO_2 (g)
      iv. Na_2O (s) + H_2O (l) $\longrightarrow$ Na^+ (aq) + 2 OH^- (aq)
      v. 2 $KClO_3$ (s) + heat $\longrightarrow$ 2 KCl (s) + 3 O_2 (g)
      vi. 2 Mg (s) + $TiCl_4$ (g) $\longrightarrow$ 2 $MgCl_2$ (s) + Ti (s)
      vii. NaCl (aq) + $AgNO_3$ (aq) $\longrightarrow$ $NaNO_3$ (aq) + AgCl (s)
      viii. Br_2 (l) + 2 NaI (aq) $\longrightarrow$ 2 NaBr (aq) + I_2 (s)

ix. HNO_3 (aq) + H_2O (l) $\longrightarrow$ H_3O^+ (aq) + NO_3^- (aq)
x. 3 C (s) + 2 Fe_2O_3 (s) + heat $\longrightarrow$ 2 Fe (l) + 3 CO_2 (g)
xi. Al_2O_3 (s) + H_2O (l) $\longrightarrow$ no reaction
xii. $CaSO_3$ (s) + heat $\longrightarrow$ CaO (s) + SO_2 (g)
xiii. 2 HNO_3 (aq) + $Ba(OH)_2$ (aq) $\longrightarrow$ $Ba(NO_3)_2$ (aq) + 2 H_2O (l)
xiv. Zn (s) + H_2O (l) + heat $\longrightarrow$ ZnO (s) + H_2 (g)
xv. SO_3 (g) + H_2O (l) $\longrightarrow$ H_2SO_4 (aq) (added to compare to Na_2O and Al_2O_3 rxns with H_2O)
xvi. NaCl (aq) + $AgNO_3$ (aq) $\longrightarrow$ $NaNO_3$ (aq) + AgCl (s)
xvii. 2 HgO (s) + heat $\longrightarrow$ 2 Hg (l) + O_2 (g)
xviii. Cu (s) + 2 $AgNO_3$ (aq) $\longrightarrow$ $Cu(NO_3)_2$ (aq) + 2 Ag (s)
xix. F_2 (g) + 2 KCl (s) $\longrightarrow$ 2 KF (s) + Cl_2 (g)
xx. $MgSO_4$ (s) + heat $\longrightarrow$ MgO (s) + SO_3 (g)

3. Please take a few minutes to memorize the following table:

ion name	chemical formula
nitrate	NO_3^-
carbonate	CO_3^{2-}
sulfate	SO_4^{2-}
phosphate	PO_4^{3-}
hydroxide	OH^-
ammonium	NH_4^+

Please find the values of n for each of the compounds below:

(a) Al_nPO_4, aluminium phosphate
(b) H_nSO_4, sulfuric acid
(c) $(NH_4)_nNO_3$, ammonium sulfate
(d) NaH_nCO_3, sodium bicarbonate
(e) $Sc(OH)_n$

1.13 Chem 1070 workshop 13: Reaction chemistry II

1. In each of the three "reactions" below, state what reaction or reactions have taken place. If there is a limiting reagent in one of the reactions, please state what this limiting reagent is.

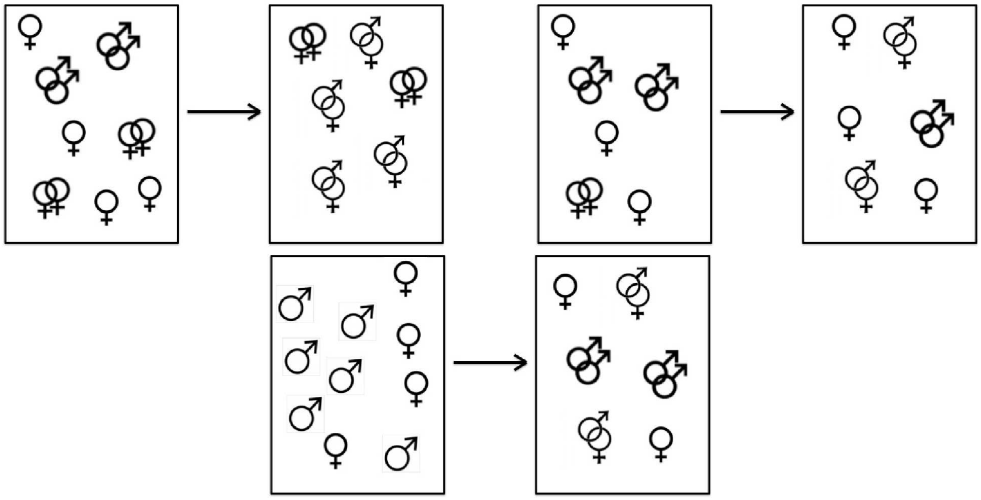

2. Please write down unbalanced chemical reactions which correspond to the following situations. Remove all non-participating molecules/ions from your chemical reactions. *Remember to keeep separate reactions separate.*

   (a) The combustion of butene, C_4H_8.

   (b) Water is poured on to a mixture of sodium phosphate and barium chloride powder. (State the three reactions which actually occur in, first, the dissolution of the powder mixture and, then, subsequently in precipitation.)

   (c) Lithium metal powder is dropped into a container of liquid oxygen.

   (d) Solid magnesium carbonate is heated leading to the release of a gas.

   (e) A mixture of barium carbonate and lithium carbonate powder are heated releasing gas.

3. State two powders, which when mixed will lead to the precipitation of barium sulfate.

4. In each of the reactions below, copy down the reaction. Identify reaction type. In doing so:

   (a) For redox reactions: Identify the element being reduced and the element being oxidized. Write down the oxidation states of these elements on both the reactant side and product side of the equation. Deduce and state favorability based on the relative electronegativity of the oxidized and reduced elements (with temperature an additional potential factor).

   (b) For acid-base reactions: Identify the reactant acid, reactant base, product acid, and product base. Compare apples to apples and oranges to oranges, ie., compare either relative acid strength or relative base strength and deduce if the reaction is favorable. State in a couple of words the source of your info.

   (c) For precipitation/dissolution reactions: Base favorability of reaction on precipitation rule(s). In a couple of words, state the relevant rule.

   (d) For insertion/elimination reactions: Identify the molecule inserted or eliminated. These reactions need to be memorized.

   (a) $CCl_4 + Li \longrightarrow LiCl + C$

   (b) $AlCl_3 + B \longrightarrow BCl_3 + Al$

(c) $Fe_2O_3 + Al \longrightarrow Al_2O_3 + Fe$

(d) $NaOH$ (aq) $+ BaCl_2$ (aq) $\longrightarrow NaCl$ (aq) $+ Ba(OH)_2$ (s)

(e) $LiCl + H_2CO_3 \longrightarrow HCl + Li_2CO_3$

(f) $H_3PO_4 + Na_2CO_3 \longrightarrow NaH_2PO_4 + NaHCO_3$

(g) $CaCO_3$ (s) + heat $\longrightarrow CaO$ (s) $+ CO_2$ (g)

(h) $H_3PO_4 + Na_2CO_3 \longrightarrow NaH_2PO_4 + H_2CO_3$

(i) $MgCl_2$ (aq) $+ Na_2CO_3$ (aq) $\longrightarrow NaCl + MgCO_3$ (s)

1.14 Chem 1070 workshop 14: Reactions review I

1. Please balance the following reactions (adapted from Petrucci, *General Chemistry*):

   (a)

   $$H_5IO_6 + I_2 \longrightarrow IO_3^- \quad \text{(acidic conditions)}$$

   (b)

   $$S_2F_2 \longrightarrow S_8 + H_2S_4O_6 + HF \quad \text{(acidic conditions)}$$

   (c)

   $$O_2^- \longrightarrow O_2 \quad \text{(basic conditions)}$$

   (d)

   $$B_2Cl_4 \longrightarrow BO_2^- + Cl^- + H_2 \quad \text{(basic conditions)}$$

2. Identify the reaction type pf each **unbalanced** reactions given below. Determine whether, at STP, the reaction is thermodynamically favorable. (A thermodynamically favored reaction, when finished, has **greater** than 50% yield) If not a reaction, state NR.

   (a) K_2O (s) + H_2O (l) $\longrightarrow$ K^+ (aq) + OH^- (aq)
   (b) K_2O (s) + CO_2 (g) $\longrightarrow$ K_2CO_3(s)
   (c) HCl (aq) + NH_3 (aq) $\longrightarrow$ Cl^-(aq) + NH_4^+(aq)
   (d) HCl (g) + NH_3 (g) $\longrightarrow$ NH_4Cl(s)
   (e) KH (s) + KF (s) + H_2O (l) $\longrightarrow$ K^+ (aq) + F^- (aq) + H_2(g)
   (f) $CdSO_4$ (s) + Zn (s) $\longrightarrow$ $ZnSO_4$ (s) + Cd (s)
   (g) K_2SO_4 (aq) + $Mg(NO_3)_2$ (aq) $\longrightarrow$ $MgSO_4$ (aq) + KNO_3 (aq)
   (h) AgBr (s) + KNO_3 (aq) $\longrightarrow$ $AgNO_3$ (aq) + KBr (aq)
   (i) $CuSO_4 \cdot 5H_2O$ (s) + heat $\longrightarrow$ $CuSO_4$ (s) + H_2O (g)
   (j) HCl (aq) + SO_4^{2-} (aq) $\longrightarrow$ HSO_4^-(aq) + Cl^-(aq)
   (k) IF_5 (l) $\longrightarrow$ I_2(s) + F_2(g)

3. Which of the following is the stronger reactant (write your answers using chemical formulas),

   (a) as base: phosphate or hypochlorite
   (b) as acid: sulfuric acid or sulfurous acid
   (c) as oxidizer: nitric acid or nitrous acid
   (d) as acid: $H_2PO_4^-$ or HPO_4^{2-}
   (e) as base: $H_2PO_4^-$ or HPO_4^{2-}
   (f) as oxidizer: hydrochloric acid or sodium chlorate
   (g) as base: carbonate or bicarbonate
   (h) as acid: H_2SO_4 or HSO_4^-
   (i) as oxidizer: nitric acid or hydrochloric acid
   (j) as base: Br^- or SO_4^{2-}

4. Write a balanced chemical equation for the sum of all the reactions which occur in the situations below (taken from AP chem exams 1999-2000).

(a) Calcium oxide powder is added to distilled water.

(b) Liquid bromine is shaken with a 0.5 M sodium iodide solution.

(c) Solid lead(II) carbonate is added to a 0.5 M sulfuric acid solution.

(d) A mixture of powdered iron(III) oxide and powdered aluminium metal is heated strongly.

(e) Carbon dioxide gas is passed over wram solid sodium oxide.

(f) A 0.2 M barium nitrate solution is added to an alkaline 0.2 M potassium chromate solution.

(g) A small piece of calcium metal is added to hot distilled water.

(h) A solution of copper(II) chloride is added to a solution of sodium sulfide.

(i) A solution of tin(II) nitrate is added to a solution of silver nitrate.

(j) Excess hydrobromic acid solution is added to a solution of potassium hydrogen carbonate (potassium hydrogen carbonate is typically known as potassium bicarbonate.)

1.15 1070 workshop 15: A-level stoichimetry problems II

1. Two A-level questions in Chem 2070 Problem Set 4 are:

   (a) A metal oxide of formula M_pO (where M is an unknown metal and p is some integer, i.e. $p = 1, 2, 3, 4...$) is heated until it completely decomposes into the pure metal and oxygen gas. If 69.00 g of the metal oxide produces 11.71 g of O_2 what is the identity of the metal M?

   (b) Two unidentified elements X and Y can combine to form three different compounds, see below. If compound 1 is the binary compound XY, what are the simplest formulas for compounds 2 and 3? (answers include X_2Y_5)

Compound	Mass of X	Mass of Y
1	25.2 g	28.8 g
2	72.8 g	41.6 g
3	22.4 g	64.0 g

2. Two previous 1070 questions which bear similarities to these two problems are:

   (a) Two new and previously unidentified elements have been discovered, elements R and Z. Elements R and Z are known to form into two compounds R_2Z_3 and a second compound, which is purely composed of the two new elements, compound **Q**. The stoichiometry of compound **Q** is as yet unknown but chemical analyses have been recently performed. Based on the data below, what is the empirical formula of compound **Q**? For credit, please show your work. (answer in prelim1-1 answer key)

Compound	Mass of R	Mass of Z
R_2Z_3	38.30 g	24.20 g
**Q**	64.60 g	54.42 g

   (b) A new previously unknown element, X, has been discovered to make two different pure compounds, **R** and **Q**, when combined with uranium. The elemental mass compostion of two pure samples of each of these new compounds are reported below. Based on this data, what are the simplest possible chemical formulas for **R** and **Q**? (answer in prelim1-1 answer key)

Compound	Mass of U	Mass of X
**R**	18.000 g	6.682 g
**Q**	46.000 g	25.599 g

3. Given below are three additional like-minded problems together with one problem taken from workshop 8.

   (a) The metallic element Thomascrappium, To, is named after Thomas Crapper, the inventor of the modern toilet. It has an atomic mass appropriately of 222.22 u (as in number 2). If 180.00 g of Thomascrappium reacts with oxygen to form 212.40 g of the metal oxide To_xO_y, what are the values of x and y?

   (b) A new metal Z reacts with all the halogens. The reaction, $ZCl_x + F_2 \longrightarrow ZF_x + Cl_2$, is known to occur. 267.3 g of ZCl_x react to form 244.7 g of ZF_x. What is the value of x? What is the atomic mass of Z? (num answer 218.60 g/mol)

   (c) A new alkali metal, Q, has been discovered in alloys of cobalt, CoQ_x, where x is not a whole number. This alloy reacts in cold water to produce soluble Q^+, hydrogen gas, and pure cobalt metal. Write a balanced equation, incorporating the value of x in the final answer. It is observed that the reaction of 33.2 g of the alloy, at the above conditions, results in 18.4 g of pure cobalt and 0.0187 g of hydrogen gas. What is the atomic mass of Q? (num. answer: 799 g/mol.)

(d) Three new and previously unidentified elements have been discovered, elements X, Y, and Z. Elements X and Y are known to form a compound containing no Z, with chemical formula X_2Y_3. Elements Y and Z are known to make a second compound, containing no X, with chemical formula YZ_2. Finally, all three elements are known to make a third compound, compound **Q**, of unknown stoichiometry. Compound **Q** is only composed of elements X, Y, and Z. Based on the data below, what is the empirical formula of compound **Q**?

Compound	Mass of X	Mass of Y	Mass of Z
X_2Y_3	58.40 g	115.39 g	—
YZ_2	—	86.40 g	40.38 g
**Q**	47.11 g	186.17 g	72.51 g

1.16 1070 workshop 16: Overview for balancing chemical reactions

1. The two approaches to the balancing of chemical reactions are:

   (a) **The half-reaction method**

      i. *Method should always be used if the reaction is an aqueous redox reaction where a reactant or product is an ion, and there is an atom whose oxidized and reduced forms are self-evident.*

      ii. Method can not be used if not a redox reaction.

      iii. Method is preferable for aqueous redox reactions where water's role as a reactant or product is unknown.

      iv. Method can be used to avoid algebra.

   (b) **The iterative method**

      i. *Method should always be used for non-redox reactions.*

      ii. Method can be used in non-aqueous redox reactions.

      iii. Method can be used in aqueous redox reactions if water is listed as product or reactant and there are no ions in the reaction.

      iv. Method can evolve into algebra.

2. Please balance the following reactions:

   (a) $BaCO_3 + HNO_3 \longrightarrow Ba(NO_3)_2 + CO_2 + H_2O$

   (b) $CaSiTe + CaTe_2 \longrightarrow Ca_2Si + SiTe_4$

   (c) $MnO_4^- \longrightarrow MnO_2 + O_2$ (in mild base)

   (d) $Sb_2S_3 + Na_2CO_3 + C \longrightarrow Sb + Na_2S + CO$ (in base)

   (e) $Fe_3Co_2C_2 + Fe_2Co \longrightarrow Fe_4C + Co_2C$

   (f) $NO_2(aq) \longrightarrow NO(g) + NO_3^-(aq)$

   (g) $CuSCN(s) + HCl(g) + AgIO_3(s) \longrightarrow CuSO_4(s) + ICl(aq) + AgCl(s) + HCN(aq)$

   (h) $H_2NCONH_2 + H_2O_2 + C_2H_6 \longrightarrow NH_3 + CO_2$

   (i) $FeSO_4 + K_3[Fe(CN)_6] \longrightarrow Fe_3[Fe(CN)_6]_2 + K_2SO_4$

   (j) $O_2^- \longrightarrow O_2$ (in base)

   (k) $CaHPO_4 \cdot H_2O + NaOH + H_2O \longrightarrow Na_2HPO_4 \cdot 12H_2O + Ca(OH)_2$

   (l) $P_2I_4 + P_4 + H_2O \longrightarrow PH_4I + H_3PO_4$

   (m) ClO_2 (aq) $\longrightarrow HClO_2$ (aq) $+ HClO_3$ (aq)

   (n) $VO_2Cl + NH_4OH \longrightarrow NH_4VO_3 + NH_4Cl + H_2O$

   (o) $I_2 \longrightarrow I^- + IO_3^-$ (in base)

   (p) $NH_4^+ + NO_3 \longrightarrow N_2O + H_2O + H^+$

   (q) $NaO_2H \longrightarrow NaOH + O_2$ (in base)

   (r) $KI + KClO_3 + HCl \longrightarrow I_2 + H_2O + KCl$

   (s) $H_3PO_4 + (NH_4)_2 \cdot MoO_4 + HNO_3 \longrightarrow (NH_4)_3PO_4 \cdot 12MoO_3 + NH_4NO_3 + H_2O$

   (t) $H_2O_2 \longrightarrow H_2O + O_2$

   (u) $K_4Fe(CN)_6 + Ce(NO_3)_4 + KOH \longrightarrow Ce(OH)_3 + Fe(OH)_3 + K_2CO_3 + KNO_3$ (in base)

1.17 1070 workshop 17: Percentages, ratios, and averages in gen-chem

1. This collection of problems is designed firm up students' use of ratios, averages, and percentages. Starred problems, in this set represent questions written at an A-level. A-level questions may help strengthen cognitive abilities, but are at a level rarely seen in actual Chem 2070 examinations.

   (a) A surprising number of students can solve this problem incorrectly: If there is a 2:1 ratio of ^{300}Uus and ^{306}Uus, what is Uus's atomic mass? But note, the answer must be a number twice as close to 300 as 306. The answer is therefore 302 amu. With that is mind, without a calculator, please solve the following three questions from an earlier workshop:

      i. 99% of carbon atoms are ^{12}C, almost all the remaining are ^{13}C. To four significant figures, what is carbon's atomic weight?

      ii. 93% of lithium atoms are ^{7}Li, almost all the remaining are ^{6}Li. To three significant figures, what is lithium's atomic weight?

      iii. 76% of chlorine atoms are ^{35}Cl, almost all the remaining are ^{37}Cl. To three significant figures, what is chlorine's atomic weight?

   (b) A C-H-O compound has a 5:8 molar ratio of carbon to hydrogen and is 48.4% by mass oxygen. What is its empirical formula?

   (c) 119.675 g of a pure but unknown compound is subjected to a hot I_2 gas. The original compound entirely reacts producing 227.110 g of CI_4 and 14.5918 g of I_2O_5 and no other compounds. What is the empirical formula for this compound?

   (d) You wish to make a 10.00 L stock solution which is 0.100 M Na^+ and 0.400 M Cl^-. You have on hand 1.00 L of 2.00 M NaCl and a large bottle of barium chloride. What percent of the NaCl solution should you use? Having decided to first prepare a barium chloride solution to which you will add the chosen fraction of the NaCl solution, thus producing the desired 10.00 L stock solution, what should the molarity and volume of this barium chloride solution be?(num. answer: 0.158 M)

   (e) A pure initial compound containing no chlorine is reacted with pure chlorine gas. The only compounds produced are 5.5398 g HOCl, 3.8503 g HCl, and 16.2437 g CCl_4. After reaction, none of the initial compound remains. What is the empirical formula of the initial compound?

   (f) A C-H-O compound has a 2:5 molar ratio of carbon to hydrogen and a 2.50:1.00 mass ratio of carbon to oxygen. What is its empirical formula?

   (g) The combustion of 1.38 g of a compound, which contains the four elements C, H, O, and N yields 1.72 g of CO_2 and 1.18 g H_2O. Another sample of the same compound with a mass of 22.34 kg is found to contain 6.75 kg of O atoms. What is the empirical formula of the compound?

   (h) Si has three stable isotopes, ^{28}Si with a 92.230% natural abundance and an atomic mass of 27.977 amu and ^{30}Si with a 3.087% natural abundance and an atomic mass of 29.974 amu. What is the third Si isotope, and what is its mass in amu?

   (i) A compound is 62.5 mass percent carbon and 4.19 mass percent hydrogen. The only remaining element is sulfur. 0.782 g of this compound dissolved in 42.0 mL of solution is 0.0968 Molar. What is the molecular formula for this compound?

   (j) A mystery compound, Y, has *empirical* formula $C_2H_3O_2S$. Compound Y reacts with aqueous sodium hydroxide to produce water and the compound Z according to the *balanced* equation, $2NaOH(aq) + Y \longrightarrow 2H_2O(aq) + Z(aq)$. 5.00 g of compound Y and 27.5 ml of 2.0 M NaOH(aq) react completely, with neither material left over. What is the *molecular* formula of Y? (Source: Cornell PSP chem class)

   (k) * To my knowledge, only two elements have a smaller atomic mass than the element immediately preceding it in the periodic table: nickel and iodine. This problem concerns the first of these two elements, nickel. Nickel has five stable isotopes: ^{58}Ni, ^{60}Ni, ^{61}Ni, ^{62}Ni, and ^{64}Ni. The percent abundance of ^{61}Ni, ^{62}Ni, and ^{64}Ni are respectively 1.14%, 3.63%, and 0.93%. The element preceding nickel is cobalt which has only one stable isotope, ^{59}Co. What would the minimum percent abundance of ^{58}Ni have to be in order for nickel's atomic mass to be less than cobalt's?

(l) * A metal oxide forms an oxide X. X has the empirical formula MO_2 and is 13.38 mass percent oxygen. When heated, X gives off oxygen and converts to Y, which is 9.335 mass percent oxygen. What is the identity of the metal M? What is the empirical formula of compound Y? (Source: Cornell PSP chem class)

1.18 Chem 1070 workshop 18: A deeper understanding of the oil-drop and cathode ray experiments

1. In this problem, we explore, in detail, how Millikan determined the charge of the electron. Among the issues explored, is how Millikan used the frictional force to deduce the mass of the oil droplets, a necessary step in the determination of the electron charge.

   (a) The oil drop experiment can be thought to have two parts. The first part leads to the determination of the mass of the tiny oil droplets. Tiny oil droplets quickly reach a state where they fall at constant velocity (the terminal velocity): in other words they fall with zero acceleration. The following equations hold true: (1) $F_{friction}+F_{gravity} = m_{drop}a$; (2) $F_{friction} = 6\pi r\eta v$, where r is the radius of the drop, η is the viscosity, and v is the terminal velocity; and (3) $F_{gravity} = 4\pi\rho g r^3/3$, where ρ and g are respectively the density of oil and the Earth's gravitational constant.

   Find an expression for r in term of ρ, η, v, and g (all of which are experimentally determinable quantities).

   (b) In the second part of the experiment, we slowly turn on the electric field on this same oil droplet whose terminal velocity has been previously observed until the droplet ceases to fall. At this point, the droplet no longer feels $F_{friction}$ (as it is not moving) but experiences both $F_{gravity}$ and $F_{electrostatic}$, where $F_{electrostatic} = q_{drop}E$ and E is the electric field. In this case, $F_{electrostatic} + F_{gravity} = m_{drop}a$, where the acceleration is again zero.

   Using in part your previous answer, find an expression for q_{drop} in term of ρ, η, v, E, and g (all of which are experimentally determinable quantities).

   (c) A student describing the Millikan oil drop experiment writes, "Finding r is in many ways the key step in the Millikan oil experiment. Determination of r allows the scientist to determine the mass of the droplet. As in a suspended droplet $q_{drop}E = m_{drop}g$, knowing m_{drop} is essential in finding q_{drop}. Do you agree with this student or not? Explain your answer.

   (d) A key step in the Millikan oil drop experiment is that the charges of the oil droplets are Nq_e, integer multiples of the electron charge. To understand how we can deduce q_e from Nq_e, please answer this related problem.

   An inspector is given a collection of twelve boxes, all filled with melons. The material from which the boxes are made is very light. The inspector weighs the boxes and discovers the boxes weigh 9, 9, 9, 6, 12, 9, 12, 15, 9, 9, 6, and 12 pounds. Can the inspector deduce out how much the individual melons weigh without opening the boxes, and if she can, how much does she expect the melons to weigh? (Assume all melons weigh the same amount.)

   (e) Please explain how Millikan was able to deduce q_e from Nq_e.

   (f) For the first time, what value did the Millikan oil drop experiment provide to scientists?

2. We now present a full treatment for the Thomson cathode-ray experiments.

   (a) Like the Millikan oil drop experiment, the Thomson experiment consists of two parts. The first part involves ray deflection by an electric field. Useful equations include (1) $F = q_eE$, where E is the electric field; (2) $F = m_ea$, where a is the acceleration; (3) $d = v_xt_F$, where t_F, d, and v_x are respectively the time the electron is between the charged metal plates, the length of the charged plates, and the horizontal electron velocity; and (4) $v_y = at_F$, where v_y is the velocity of the electron in the deflected direction. We note that while neither v_x nor v_y are themeselves directly observable, the ratio v_y/v_x is determinable from the angle of ray deflection. Find an expression for q_e/m_e in terms of d, E, v_y, and v_x.

   (b) The second part of the Thomson experiment involves ray deflection by a magnetic field. The chosen magnetic field deflects the ray the same magnitude as did the previous electric field. Equations are similar here except now $F' = q_ev_xB$ with $F' = m_ea$ and where B is the magnetic field. Find an expression for q_e/m_e in terms of d, B, and v_y.

   (c) Using your two results above, find an expression for v_x in terms of E and B.

(d) Using your last two answers, find an expression for q_e/m_e in terms of v_y/v_x, B, E, and d. (All terms on the right side of this equation are experimentally determinable.)

(e) A student summarizes the cathode ray experiments with the following observation. "The most clever part of the cathode ray experiments were the separate experiments involving either the magnetic field or the electric field. From either the electric field or the magnetic field experiments, it was clear that the angle of ray deflection was proportional to the ratio q_e/m_e. But the proportionality constant relating the angle of deflection to q_e/m_e could not be determined without prior knowledge of the initial electron velocity. By using the two experiments together, Thomson was able to deduce this initial velocity." Explain in your own words what the student meant by the above statement.

(f) In your opinion, what were the key steps in the Thomson experiment?

(g) For the first time, the combination of the Millikan oil drop and Thomson cathode ray experiments provided scientists with what quantity?

1.19 Chem 1070 Practice prelim 1-1

1. (35 pts) Sulfur and arsenic can react to form As_4S_3. A student carried out this reaction by placing 75.0 g of As together with 30.0 g of S in a clean glass tube, sealing the tube, and heating the mixture to 200.0^{o}C for three weeks. After the reaction, the student cooled the tube down to room temperature, broke open the tube, and then using X-ray powder diffraction correctly determined the amount of As, S, and As_4S_3 in her reacted sample.

   The student determined that the reaction occured to only 43.4% of the maximal theoretical yield (ie., 43.4% of maximal completion). How many g of elemental As, elemental S, and As_4S_3 were there in the glass tube after the reaction had occured? For credit, please show your work.

2. (35 pts) A problem taken from Chapter 2 of your textbook is the following: "A 40.0 milligram sample of the compound X_4O_{10} contains 22.5 milligrams of oxygen atoms. What is the atomic mass of element X?" (Note the atomic mass of an element is the weight of one mole of that element.) Please answer this question. For credit, show your work.

3. (35 pts) Two new and previously unidentified elements have been discovered, elements R and Z. Elements R and Z are known to form into two compounds R_2Z_3 and a second compound, which is purely composed of the two new elements, compound **Q**. The stoichiometry of compound **Q** is as yet unknown but chemical analyses have been recently performed. Based on the data below, what is the empirical formula of compound **Q**? For credit, please show your work.

Compound	Mass of R	Mass of Z
R_2Z_3	38.30 g	24.20 g
**Q**	64.60 g	54.42 g

4. (35 pts) A sealed flask contains initially 97.3 g of Li_3Bi and 130.6 g of HgF_2. The flask is heated and the **unbalanced** chemical reaction occurs:

$$Li_3Bi + HgF_2 \longrightarrow Hg_3Bi_2 + LiF.$$

   30.0 g of Li_3Bi remain at the reaction end. How many grams of HgF_2, Hg_3Bi_2, and LiF were there in the flask at the end? What was the percent yield (ie., what was the percent ratio of the actually observed amount of reaction compared to the amount of reaction which would have occurred had the reaction gone to maximal completion)? For credit, show your work.

   A really good way to solve this problem is with an ICE table. In an ICE table, sometimes, the student needs to calculate in both upwards and downwards directions. Remember in setting up this ICE table that you need to first balance the chemical reaction. And don't forget to identify the limiting reagent!

5. (25 pts) The graph below portrays a gaseous chemical reaction. What true **balanced** gaseous chemical reaction equation took place? What is the true chemical reaction's percent yield?

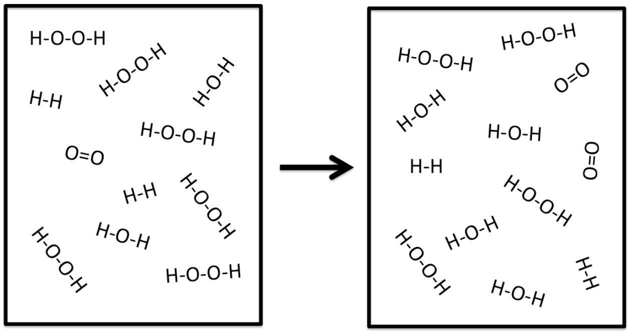

6. (35 pts) A new previously unknown element, X, has been discovered to make two different pure compounds, **R** and **Q**, when combined with uranium. The elemental mass compostion of two pure samples

of each of these new compounds are reported below. Based on this data, what are the simplest possible chemical formulas for **R** and **Q**? For credit, show your work.

Compound	Mass of U	Mass of X
**R**	18.000 g	6.682 g
**Q**	46.000 g	25.599 g

1.20 Chem 1070 Practice prelim 1-2

1. (25 pts) Please provide brief answers.

   (a) Element 108 in the periodic table Hassium (Hs) has an atomic mass of 277 amu while the very next element in the periodic table, element 109, Meitnerium (Mt), has a lighter atomic mass of 268 amu. Explain why this might be so.

   (b) The prefix cath- is from the greek word kata, downwards. What is the ray in the cathode ray made up of. Briefly explain, using an equation in your explanation, why in a magnetic field this ray might be considered downwards?

   (c) A new halide X has been discovered. Please rank the following compounds from smallest mass percent X (**on the left**) to highest mass percent X (**on the right**).

   $$SeX_2,\ BiX_2,\ Bi_4X_7,\ \text{and}\ Bi_2X_3$$

   (d) What does the plum in the plum pudding model consist of?

2. P_4O_{10} can be referred to as phosphoric acid anhydride as water adds to P_4O_{10} resulting in phosphoric acid:

   $$P_4O_{10} + H_2O \longrightarrow H_3PO_4.$$

   14.307 g of P_4O_{10} and 3.600 g of water are reacted together. 8.253 g of P_4O_{10} remain at the end of the reaction. What was the percent yield?

3. (25 pts) An unknown metal, M, is a component of the hydrated phosphate $M_nPO_4 \cdot 3H_2O$. Two samples of this hydrated phosphate are prepared. In the first sample there are 0.312 g of M and 0.147 g of phosphorus. In the second sample there are 0.516 g of the unknown metal M. How many moles of oxygen atoms are there in the second sample?

4. (25 pts) Chlorine has only two stable isotopes, ^{35}Cl and ^{37}Cl, with respective masses of 35.0 and 37.0 amu.

   (a) To three sig figs, calculate the percent of natural chlorine which are ^{35}Cl and ^{37}Cl. For credit, show your work.

   (b) Individual Cl_2 molecules are found to have three different masses. please calculate the percent abundance of each of these three masses in a sample of chlorine gas. For credit, show your work.

5. (25 pts) In an oxidation-reduction (redox) reaction, at least one element/atom is oxidized (loses electrons) and one element/atom is reduced (gains electrons). In each of the unbalanced reactions below, determine if the described reaction is a redox reaction. If the reaction is a redox reaction, identify at least one element/atom in the given reaction which is reduced. For this identified element/atom, specify what the initial and final oxidation states are.

   (a)

   $$Li_2S + HgBr_2 \longrightarrow HgS + LiBr.$$

   (b)

   $$IO_3^-(aq) + I^- + H^+(aq) \longrightarrow I_2(g) + H_2O(aq).$$

   (c)

   $$Ca(OH)_2 + C_6H_8O_7 \longrightarrow Ca_3(C_6H_5O_7)_2 + H_2O$$

6. (25 pts) A pure unknown compound undergoes combustion analysis. The carbon dioxide produced by the combustion reaction is captured by NaOH in the form of $NaHCO_3$, while water produced by the combustion reaction is sequestered by $Mg(ClO_4)_2$ as $Mg(ClO_4)_2 \cdot 6H_2O$.

   The molecular weights of $NaHCO_3$ and $Mg(ClO_4)_2 \cdot 6H_2O$ are respectively 84.01 and 331.30 g/mol.

   If through the combustion and the above procedure 11.85 g of this unknown compound result in 47.95 g of $NaHCO_3$ and 11.819 g of $Mg(ClO_4)_2 \cdot 6H_2O$, what is the empirical formula of the unknown compound?

7. (25 pts) The graph below portrays a chemical reaction where at least one reactant and one product are solids. What chemical reaction equation does the portrayal best describe? What is the chemical reaction's percent yield?

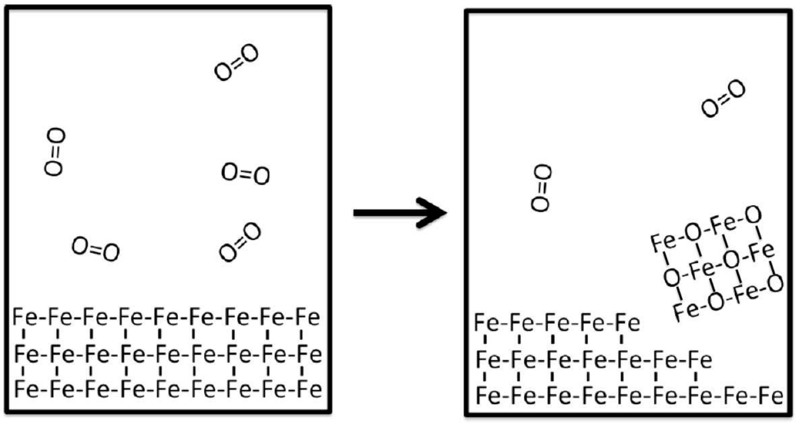

8. (25 pts) Iridium has two stable isotopes, the one being 1.61 times more abundant than the other. There is certainly no stable isotope of iridium with an amu greater than 193.5, What are the two stable iridium isotopes? Which is the more common? For credit, write your answer as **an argument**, which definitively **proves** that your answers to the above questions are correct.

1.21 Chem 1070 Practice prelim 1-3

1. (25 pts) Please answer the following short questions:

   (a) What would be the full symbol name for the single atom which has one more proton, two more electrons, and three more neutrons than an α-particle?

   (b) On the island Bourbaki in the southern Indian Ocean, charges are measured in units of bubus. A Bourbakian scientist carrys out the Millikan oil drop experiment on eight droplets and discovers that the charges of the droplets are respectively 14, 21, 14, 28, 14, 35, 14, and 63 bubus. In bubus, what is the most probable charge of the electron?

   (c) When measuring the resistivity of metals or semi-metals or non-metals, state how resistivity depends on temperature. *Resistivity is the opposite of conductivity.*

   (d) Historically, what experiment(s) needed to be carried out to first determine the mass of the electron?

2. (25 pts) 27.356 g of an unknown compound composed of exactly two elements is heated in the presence of oxygen. Two and only two compounds are produced: B_2O_3 and 53.430 g of water. None of the starting material survives the heating process. What is the empirical formula of this unknown compound?

3. (25 pts) A homogenous rock is to be assayed for its chromium content. 12.0 g of this rock is crushed, dissolved in sulfuric acid, and passed over a reducing agent so that all the chromium is in the form of Cr^{2+} (aq). The Cr^{2+} (aq) is then reacted to completion by exactly 31.0 mL of 0.582 M NaI_3, according to the reaction:

$$I_3^- \text{ (aq)} + Cr^{2+} \text{ (aq)} \longrightarrow Cr^{3+} \text{ (aq)} + I^- \text{ (aq)}.$$

   What mass percent of the rock is chromium? For credit, show your work. (Adapted from a problem in Chapter 24 of your textbook.)

4. (25 pts) 19.0 mL of 0.100 M $Ba(OH)_2$ is mixed with 31.0 mL of 0.150 M HCl. The resulting acid-base reaction proceeds to completion. How many moles of water were produced by the reaction? For credit, show your work.

5. (25 pts) Chemical reactions:

   (a) Write **balanced equations** that include the products of the reaction below. If no reaction occurs designate it as such (NR). Specify the physical state of all products. For products in aqueous solution denote products as precipitates (s) or aqueous ions (aq).

      i. $MgSO_4$ (aq) + $Ba(OH)_2$ (aq) $\longrightarrow$
      ii. H_3PO_4 (aq) + ClO_4^- (aq) $\longrightarrow$
      iii. $HgCH_3CO_2$ (aq) + HI (aq) $\longrightarrow$
      iv. $NaCH_3CO_2$ (aq) + $(NH_4)_2CO_3$ (aq) $\longrightarrow$

   (b) Using the letter designations of the reactions i. to iv. **order** the following four reactions from least (**on the left**) to most reactive (**on the right**).

      i. $Ca(OH)_2$ (s) + H_2O(l) $\longrightarrow$
      ii. $Ca(OH)_2$ (s) + HCl(aq) $\longrightarrow$
      iii. $Ba(OH)_2$ (s) + HCl(aq) $\longrightarrow$
      iv. $Ca(OH)_2$ (s) + HF(aq) $\longrightarrow$

6. (25 pts) Throughout the galaxy, there are only two stable thallium isotopes, ^{203}Tl and ^{205}Tl. Natural thallium from the planet Pantoufle however has an atomic mass of 203.62. By what multiplicative factor is the fraction of abundance of the isotope ^{203}Tl **greater** on the planet Pantoufle than it is on Earth? For credit, show your work.

7. (25 pts) Both Millikan's oil drop experimental apparatus and the mass spectrometer can be thought to contain charged metallic plates. In both cases, between these plates, $F_{electrostatic} = qE$, where q is the charge of the particle and E is the electric field.

   The use of pictures is encouraged in your answers below. Equations/proportionality relations, however, are required. The use of Newton's second law, the sum of the forces acting on any particle equals the mass times the acceleration of this same particle, **is also required.**

   (a) In the oil drop experiment the electrostatic force is combined with the gravitational force, $F_{gravity} = mg$, where g is the Earth's gravitational constant. Using **explicitly** Newton's second law, show how Millikan could deduce that for a suspended droplet

   $$q_{droplet} = \frac{m_{droplet}g}{E}.$$

   (b) Again, using **explicitly** Newton's second law, show how the angle of deflection of a charged particle passing through a mass spectrometer depends on the mass of the charged particle itself, hence the name, mass spectrometer.

8. (25 pts) An 11.34 g mixture of $CaCO_3$ and $MgCO_3$ is heated to drive off carbon dioxide leaving behind 6.01 g of CaO together with MgO. Calculate the mole percent of $CaCO_3$ in the original mixture. For credit, please show your work. (Adapted from a problem in Chapter 11 of your textbook.)

1.22 Chem 1070 Practice prelim 1-4

1. (20 pts) Short answer questions

   (a) What would be the full symbol name for the single atom which has one more neutron, two more protons, and two more electrons than a β-particle?

   (b) The newly discovered element Uus is prepared in a 2:1 ratio of ^{300}Uus to ^{306}Uus. What is this Uus's atomic mass?

   (c) When measuring the resistivity of metals or semi-metals, state how resistivity depends on temperature.

   (d) Please rank the following substances from weakest oxidizing agent (**on the left**) to strongest oxidizing agent (**on the right**). For credit, write your list using **the correct chemical formulas.**

   sodium, nitric acid, and hydrochloric acid

2. (24 pts) In the Rutherford gold foil experiment a stream of alpha particles struck a gold foil. While most of the time the alpha particles passed through the foil unscathed, every now and then an alpha particle was deflected backwards. This question examines several what-ifs.

   (a) Had the plum pudding model been the correct description of matter, what would Rutherford have observed? For credit, explain briefly your answer.

   (b) What would Rutherford have observed had he used a stream of neutrons instead of a stream of alpha particles? For credit, explain briefly your answer.

   (c) Rutherford used gold foil, gold being highly malleable and making the thinnest of all possible foil. What would have happened had Rutherford used another metal, say tin, whose foil was fifty times thicker than that of gold? For credit, explain briefly your answer.

3. (28 pts) Write **balanced equations** that include the products of the reaction below. If no reaction occurs designate it as such (NR). Specify the physical state of all products. For products in aqueous solution denote products as precipitates (s) or aqueous ions (aq).

   (a) $H_3PO_4\,(aq) \quad + \quad ClO_4^-\,(aq) \longrightarrow$

   (b) $CaO\,(s) \quad + \quad H_2O\,(l) \longrightarrow$

   (c) $Fe_2O_3\,(s) \quad + \quad K\,(s) \longrightarrow$

   (d) $H_2SO_4\,(aq) \quad + \quad BaO\,(s) \longrightarrow$

   (e) $HF \quad + \quad Cl_2 \longrightarrow$

   (f) $MgO \quad + \quad CO_2 \quad + \quad \text{high heat} \longrightarrow$

   (g) $NaOH\,(aq) \quad + \quad (NH_4)_2CO_3\,(aq) \longrightarrow$

4. (20 pts) Write **unbalanced reactions** which correctly describe the **sum of all** reactions which take place in the following circumstances. Write for each compound/molecule/ion the correct states of matter. (Adapted from the former syllabus 2005 AP chemistry examination)

   (a) Solid aluminium hydroxide is placed in a concentrated solution of hydrochloric acid

   (b) Lithium metal is strongly heated in nitrogen gas.

   (c) Equal volumes of 0.1 M solutions of lead(II) nitrate and magnesium iodide are combined.

   (d) Carbon dioxide is bubbled into a solution of barium hydroxide

5. (28 pts) The reaction below is a complex one, with components of a redox reaction, an acid-base reaction and a dissolution/precipitation reaction.

$$K_2Cr_2O_7\,(aq) + HCl\,(aq) \longrightarrow KCl\,(aq) + CrCl_3\,(s) + Cl_2\,(g).$$

(a) Please balance the reaction

(b) Three quick questions about this reaction:

i. Which atom is reduced?

ii. Which molecule(s) contains the conjugate base?

iii. Which solubility rule does the reaction not follow?

(c) (12 pts) 250.0 mL of a 0.23 M HCl solution reacts completely with an unknown volume of a 0.51 M $K_2Cr_2O_7$ solution. What was this unknown volume?

6. (24 pts) Consider the reaction involving the polyatomic anion $Fe(CN)_6^{3-}$:

$$FeSO_4\ (aq) + K_3[Fe(CN)_6]\ (aq) \longrightarrow Fe_3[Fe(CN)_6]_2\ (s) + CrCl_3\ (s) + K_2SO_4\ (aq).$$

(a) Please write the balanced net ionic reaction.

(b) Is this reaction:

i. a redox reaction

ii. a Brønsted acid-base reaction

iii. a precipitation/dissolution reaction

iv. a combination of at least two of the above?

(c) (16 pts) If 54.3 mL of 1.2 M $FeSO_4$ and 100.8 mL of 0.53 M $K_3[Fe(CN)_6]$ solutions are mixed and react to 72.0% yield, how many grams of solid are recovered? ($M(Fe_3[Fe(CN)_6]_2)$= 591.49 g/mol)

7. (28 pts) Two combustion problems.

(a) The combustion of 3.295 g of the **first** unknown compound, yields only 7.623 g of CO_2 and 2.601 g H_2O. What is the empirical formula of the first unknown compound?

(b) The **second** unknown compound contains only the three elements C, H, and O. Combustion of a sample of the second unknown compound yields exactly the same amount of carbon dioxide and water as before: 7.623 g of CO_2 and 2.601g H_2O. However a sample of this same second unknown compound weighing 21.515 g is found to contain 13.103 g of O atoms. What is the empirical formula of the second unknown compound?

8. (24 pts) For the Thomson cathode ray experiment:

(a) Had Thomson placed the positive electric plate above the cathode ray and the negative electric plate below the plate, in what direction would the beam have been deflected? For credit, explain, using at least one relevant equation in your answer.

(b) Had Thomson placed the North pole of the magnetic field above the cathode ray and the South Pole below the plate, in what direction would the beam have been deflected? (The magnetic field, B, points from the North Pole to the South Pole.)For credit, explain, using at least one relevant equation in your answer.

(c) Based on the direction of deflection caused by the electric plates, could Thomson deduce the sign of the charge of the particles in the cathode ray? For credit, explain, using at least one relevant equation in your answer.

(d) Based on the direction of deflection caused by the North and South magnetic poles, could Thomson deduce the sign of the charge of the particles in the cathode ray? For credit, explain, using at least one relevant equation in your answer.

1.23 Chem 1070 Practice prelim 1-5

1. (20 pts) Short answer questions

   (a) Mc, moscovium, is element number 115. Its known isotope has 185 neutrons. Write the full symbol name of a moscovium atom which has 112 electrons.

   (b) The newly discovered element nihonium, Nh, is prepared in a 4:1 ratio of ^{285}Nh to ^{290}Nh. What is Nh's atomic mass?

   (c) Metals conduct. State four more properties which metals exhibit.

   (d) Please write the chemical formulas for the following three compounds: potassium peroxide, phosphorous acid, and potassium bicarbonate.

2. (24 pts) Nowadays, scientists can make beams out of positrons (used in modern medicine in PET scans). A positron has the same mass as the electron but has the charge of the proton.

   (a) What would have been observed in the first part of Thomson's cathode ray experiment, involving ray deflection by an electric field, had the experiment been carried out with a ray of positrons instead of electrons? State clearly your reasoning.

   (b) What change would Millikan have had to make, in the design of his oil drop experiment, if he were to have continued to be able to suspend his oil drops, had the oil drops been doped with positrons instead of electrons? State clearly your reasoning.

   (c) What change would have been observed in the ray of particles passing through a mass spectrometer if the magnitude of their charge were to have been doubled, all other factors staying the same? State clearly your reasoning.

   (d) Gold has only one stable isotope, ^{197}Au. An additional isotope is ^{199}Au, an isotope which emits beta-particles. What changes, if any, would Rutherford have observed, in his gold foil experiment, had he used a gold foil entirely comprised of ^{199}Au instead of the stable ^{197}Au? State clearly your reasoning.

3. (32 pts) Write **balanced equations** that include the products of the reaction below. If no reaction occurs designate it as such (NR). Specify the physical state of all products. For products in aqueous solution denote products as precipitates (s) or aqueous ions (aq).

   (a) $Ba\,(s) \quad + \quad H_2O\,(l) \longrightarrow$

   (b) $Ba(OH)_2\,(aq) \quad + \quad Al_2(SO_4)_3\,(aq) \longrightarrow$

   (c) $Co\,(s) \quad + \quad H_2O\,(l) \longrightarrow$

   (d) $Ni\,(s) \quad + \quad HCl\,(aq) \longrightarrow$
   Ni's preferred oxidation state is Ni(II)

   (e) $HBr\,(g) \quad + \quad Cl_2\,(g) \longrightarrow$

   (f) $HClO_3\,(aq) \quad + \quad NaHCO_3\,(aq) \longrightarrow$
   In an open flask and under normal laboratory conditions.

   (g) $Ti\,(s) \quad + \quad Br_2\,(l) \longrightarrow$

   (h) $Ca(OH)_2\,(aq) \quad + \quad (NH_4)_2SO_4\,(aq) \longrightarrow$

4. (24 pts) The reaction below takes place at 400° C.

$$Cu_3CdZn_2\,(s) + CdZn_3\,(s) \longrightarrow CuZn_4\,(s) + Cu_2Cd\,(s).$$

   (a) Please balance the reaction.

   (b) 11.41 g of Cu_3CdZn_2 react with 31.32 g of $CdZn_3$ to completion. How many grams are there of each of the four compounds at the reaction's end? $M(Cu_3CdZn_2) = 433.87$g/mol; $M(CdZn_3) = 308.64$ g/mol; $M(CuZn_4) = 325.18$g/mol; and $M(Cu_2Cd) = 239.50$g/mol.

5. (24 pts) Consider the reaction which takes place in acidic solution:

$$Cu_3P\ (s) + Cr_2O_7^{2-}\ (aq) \longrightarrow Cu^{2+}\ (aq) + H_3PO_4\ (aq) + Cr^{3+}\ (aq).$$

(a) Please write the balanced net ionic reaction (the balanced coefficients are enormous).

(b) Specify the oxidation states of the Cu, P, and Cr atoms before and after the reaction has taken place. Make sure that you have identified clearly whether the oxidation states you have expressed refer to the reactants or products.

(c) If 1.32 g of Cu_3P are required to neutralize 11.3 mL of sodium dichromate, what was the Molarity of the original dichromate solution? $M(Cu_3P) = 221.62$ g/mol.

6. (24 pts) You need to know two facts to solve the problem below. (1) Bicarbonates, HCO_3^-, like nitrates are always soluble. (2) Under normal laboratory conditions, over time, carbonic acid separates completely into water and carbon dioxide. The carbon dioxide, which comes off as bubbles, can be collected and weighed.

A 2.0 L solution has silver nitrate, barium nitrate, ammonium nitrate and cobalt(II) nitrate mixed together in solution. How can we, through precipitation, acid-base reactions, and through the accumulation of gas, determine the original Molarity for each of the four nitrates (silver, barium, ammonium and cobalt (II) nitrates) present in the original solution? For credit state your exact proposed procedure. A flow chart may help in the writing up of your answer. (This problem is adapted from Problem 10-90 from your Chem 2070 Problem Set 3.)

7. (24 pts) Two samples, both 10.00 g and both mixtures of TlCl and InF_3

(a) The **first** 10.00 g sample composed of a mixture of TlCl and InF_3 was exposed to a stream of hot bromine vapor. No metal atoms were detected leaving the sample in the effluent gas. At reaction end, no chlorine nor fluorine atoms remained in the solid sample. The reacted solid sample weighed however 13.62 g.

Assuming that after reaction, the oxidation states of both metals remained unchanged from before, what was the mass percent of InF_3 in the original sample? For credit, show your work.(Problem based on Problem 11-47 of Chem 2070 Problem Set 4.)

M(TlCl) = 239.84 g/mol; $M(InF_3) = 171.82$ g/mol; M(TlBr) = 284.29 g/mol;

and $M(InBr_3) = 354.53$ g/mol.

(b) (6 pts) The **second** 10.00 g sample composed of a mixture of TlCl and InF_3 is exposed to the exact same procedure as the first sample. What is the maximum number of grams of fluorine gas which could have been accumulated for this second sample? For credit, show your work.

8. (24 pts) An unknown compound

(a) An unknown compound weighing 37.65 g is combusted resulting only in carbon dioxide, nitrogen dioxide and water. All water and carbon dioxide resulting from the combustion were trapped separately by respectively magnesium sulfate and calcium hydroxide in the form of magnesium sulfate heptahydrate and calcium carbonate. 33.12 g of $MgSO_4{\cdot}7H_2O$ $\{M(MgSO_4 \cdot 7H_2O) = 246.51$ g/mol$\}$ and 117.66 g $CaCO_3$ $\{M(CaCO_3) = 100.09$ g/mol$\}$ were collected. The mole ratio of oxygen to nitrogen in the unknown compound is 2.00 to 1.00. What is the empirical formula of this compound? For credit, show your work. (Adapted from Chem 2070 Lecture 10 and from Chem 2070 Problem Set 4.)

(b) (4 pts) The molecular mass of the unknown compound is 480.45 g/mol. What is the molecular formula of the unknown compound?

Comparison of the cognitive requirements of service course worksheets to text and problems in current university level general chemistry textbooks

To provide context we compare cognitive differences between our Cornell service course worksheets and two current university-level general chemistry textbooks. We compare differences in the presentation of proportional reasoning. Textbooks chosen are the latest editions of textbooks which the authors have used for their own classes:

1. Steven S. Zumdahl, Susan A. Zumdahl, and Donald J. DeCoste, *Chemistry 10th Edition* Cengage Learning, Boston, 2018

and

2. Ralph H. Petrucci, F. Geoffrey Herring, Jeffry D. Madura, Carey Bissonnette, *General Chemistry: Principles and Modern Applications 11th Edition* Pearsons, Canada, 2017.

As visual presentation is an integral part of the learning process, the excerpts from the above worksheets and textbooks are presented in the same format as the original source material.

Proportional reasoning

Both Zumdahl and Petrucci have similar expositions on proportional reasoning (see below). Both books begin with a section on unit conversion and dimensional analyses. They consider the scientific concept of density as a place where unit conversion can be applied. Finally, at the end of the chapter, both books present problems requiring the chain application of unit conversion in involved settings.

Our service course treatment is different. We begin by stating the key concept, which in our eyes is not unit conversion, density, or the chain application of unit conversion but simply proportionality itself.

In our worksheet, students learn that proportionality can involve either the $\propto$ sign or the = sign. The first question asked in the worksheet: using sensible letters, to write an equation representing the proportionality relation between "money spent at the gas pump and amount of gas pumped" illustrates the difficulty of the concept itself.

Initial student answers are uniformly incorrect. First answers are for example: $ = c x gallons. In this wrong answer, $ is a unit, gallons is a unit of volume (not amount) and c (for cost) is not the proportionality constant, p (for price). Students eventually arrive at a formula which properly distinguishes units from quantities and reasonable from unreasonable quantities, producing answers such as M = PV (Money spent = the Price of gas times the Volume of gas pumped).

The problem set continues to force students to think proportionally. For ideal gases, where pV=nRT, we ask if T and p are kept constant, if one doubles n, what happens to V. The absence of numbers continues forces the student to think proportionally.

When number values are finally introduced, students are asked to consider the proportional relations between the values given.

xii. A sealed flask contains an ideal gas. The flask initially is at STP (0 ^oC and 1 atm). The flask is 14.9 L big. The flask is heated until it reaches a pressure of 3 atm. What is its final temperature? (What is the relation between 22.4 and 14.9?)

Complex and convoluted unit conversion problems are not asked of the student. Our focus is on a very different aspect of the cognitive complexity engendered by proportional reasoning.

Excerpts from the Zumdahl, Petrucci, and the service course worksheets are given on the following pages.

Zumdahl:

Significant Figures and Unit Conversions (p. 34a)

45. Perform the following unit conversions.

   a. Congratulations! You and your spouse are the proud parents of a new baby, born while you are studying in a country that uses the metric system. The nurse has informed you that the baby weighs 3.91 kg and measures 51.4 cm. Convert your baby's weight to pounds and ounces and her length to inches (rounded to the nearest quarter inch).

   b. The circumference of the earth is 25,000 mi at the equator. What is the circumference in kilometers? in meters?

   c. A rectangular solid measures 1.0 m by 5.6 cm by 2.1 dm. Express its volume in cubic meters, liters, cubic inches, and cubic feet.

Density (p. 34c)

74. At room temperature the element bromine, Br_2, is a liquid with a density of 3.12 g/cm^3. Calculate the mass of 125 mL of bromine. What volume does 85.0 g of bromine occupy?

75. A sample containing 33.42 g of metal pellets is poured into a graduated cylinder initially containing 12.7 mL of water, causing the water level in the cylinder to rise to 21.6 mL. Calculate the density of the metal.

76. The density of pure silver is 10.5 g/cm^3 at 20°C. If 5.25 g of pure silver pellets is added to a graduated cylinder containing 11.2 mL of water, to what volume level will the water in the cylinder rise?

(p. 34h)

Integrative Problems

These problems require the integration of multiple concepts to find the solutions.

127. The U.S. trade deficit at the beginning of 2005 was $475,000,000. If the wealthiest 1.00% of the U.S. population (297,000,000) contributed an equal amount of money to bring the trade deficit to $0, how many dollars would each person contribute? If one of these people were to pay his or her share in nickels only, how many nickels are needed? Another person living abroad at the time decides to pay in pounds sterling (£). How many pounds sterling does this person contribute (assume a conversion rate of 1 £ = $1.869)?

128. The density of osmium is reported by one source to be 22,610 kg/m^3. What is this density in g/cm^3? What is the mass of a block of osmium measuring 10.0 cm × 8.0 cm × 9.0 cm?

129. At the Amundsen-Scott South Pole base station in Antarctica, when the temperature is −100.0°F, researchers who live there can join the "300 Club" by stepping into a sauna heated to 200.0°F then quickly running outside and around the pole that marks the South Pole. What are these temperatures in °C? What are these temperatures in K? If you measured the temperatures only in °C and K, can you become a member of the "300 Club" (that is, is there a 300.-degree difference between the temperature extremes when measured in °C and K)?

Petrucci:

Units of Measurement (p. 27)

29. Perform the following conversions from non-SI to SI units. (Use information from the inside back cover, as needed.)
(a) 68.4 in = ____ cm
(b) 94 ft = ____ m
(c) 1.42 lb = ____ g
(d) 248 lb = ____ kg
(e) 1.85 gal = ____ dm^3
(f) 3.72 qt = ____ mL

30. Determine the number of the following:
(a) square meters (m^2) in 1 square kilometer (km^2)
(b) cubic centimeters (cm^3) in 1 cubic meter (m^3)
(c) square meters (m^2) in 1 square mile (mi^2) (1 mi = 5280 ft)

Density (p. 28)

54. A vinegar sample is found to have a density of 1.006 g/mL and to contain 5.4% acetic acid by mass. How many grams of acetic acid are present in 1.00 L of this vinegar?

55. Calculate the mass of a block of iron ($d = 7.86\ g/cm^3$) with dimensions of 52.8 cm × 6.74 cm × 3.73 cm.

(p. 30)

Integrative and Advanced Exercises

74. A typical rate of deposit of dust ("dustfall") from unpolluted air was reported as 10 tons per square mile per month. **(a)** Express this dustfall in milligrams per square meter per hour. **(b)** If the dust has an average density of 2 g/cm^3, how long would it take to accumulate a layer of dust 1 mm thick?

75. In the United States, the volume of irrigation water is usually expressed in acre-feet. One acre-foot is a volume of water sufficient to cover 1 acre of land to a depth of 1 ft (640 acres = 1 mi^2; 1 mi = 5280 ft). The principal lake in the California Water Project is Lake Oroville, whose water storage capacity is listed as 3.54×10^6 acre-feet. Express the volume of Lake Oroville in **(a)** cubic feet; **(b)** cubic meters; **(c)** U.S. gallons.

81. The Greater Vancouver Regional District (GVRD) chlorinates the water supply of the region at the rate of 1 ppm, that is, 1 kilogram of chlorine per million kilograms of water. The chlorine is introduced in the form of sodium hypochlorite, which is 47.62% chlorine. The population of the GVRD is 1.8 million persons. If each person uses 750 L of water per day, how many kilograms of sodium hypochlorite must be added to the water supply each week to produce the required chlorine level of 1 ppm?

Cornell service course worksheet 2 excerpt:

(b) For each of the pairs of quantities below, please determine if a proportionality relation exists. If a proportionality relation does exist:

i. Write the equation relating the quantities. Choose sensible letters for each of the quantities (eg., p for pressure and V for volume). You will need to identify the proportionality constant, giving this constant an appropriate letter designation as well. Remember the letter describes the quantity, **not** the unit of the quantity.

ii. If the proportionality relation is approximate use the $\approx$ symbol instead of the $=$ sign.

iii. Next to each quantity and proportionality constant write its typical unit.

iv. Write the proportionality relationship using the proportionality symbol $\propto$. Note constants do not appear in proportionality relations.

i. The money spent at the gas pump and the amount of gas pumped.

ii. Mass of a chemical sample of a molecule and the number of moles in the same sample.

iii. The distance one travels on a US interstate and the amount of time one drives (assume one drives at a steady pace).

iv. The temperature of an ideal gas and the volume of the same gas (assume the number of moles and the pressure are both constant).

v. The temperature outside and the amount of clothes one wears.

vi. The radius of a sphere and the volume of the same sphere.

xxi. The length of the side of a square and the area of the square.

xxii. The specific heat of a substance and the mass of the substance.

xxiii. The number of books on a bookshelf and the amount of available shelf space.

xxiv. Consider the amount of the solute sodium dissolved in sea water. The Molarity of the solute and the number of moles of the solute.

xxv. Consider a 1 L sample of water in which varying amounts of NaCl have been dissolved. The Molarity of solute and the number of moles of solute.

(c) Proportionality relations can be used to answer the following questions. No calculators please.

i. An ideal gas with T and p constant: one doubles n, what happens to V?

ii. One heats a kg of iron metal tripling its energy: what happens to the specific heat?

iii. An ideal gas in a sealed flask: one halves the pressure, what happenes to the volume?

iv. One halves the number of molecules in a sample of solid iron: what happens to the volume of the sample?

ix. An ideal gas in a sealed balloon in a lab : one heats the balloon until temperature doubles:, what happens to p, n, and V?

x. One mole of any ideal gas at STP ie., $0^oC = 273$ K and 1 atm pressure, always occupies 22.4 L. (Please remember the volume 22.4 L) A sealed flask contains an ideal gas. The flask initially is at STP (O ^{o}C and 1 atm). The flask is 2240 L big. How many moles of gas does the flask contain?

xi. The amount of energy, in Joules, required to heat a gram of substance 1 degree Celsius is the specific heat in J/g^{o}C . The specific heat of water is 4.18 J/g^{o}C. How many Joules are required to heat 1 kg of water 10 degrees Celsius?

xii. A sealed flask contains an ideal gas. The flask initially is at STP (0 ^{o}C and 1 atm). The flask is 14.9 L big. The flask is heated until it reaches a pressure of 3 atm. What is its final temperature? (What is the relation between 22.4 and 14.9?)

xv. When one strikes a metal coin with a hammer, energy is transferred from the hammer to the coin. A gold coin struck by a hammer gets warm quicker than a copper coin. Does gold or copper have a higher specific heat?

xvi. Ethyl alcohol has a density 4/5 that of water. How much less does 3 L of ethyl alcohol weigh than 3 L of water?
