# Supplementary material for: Patching a leak in an R1 university gateway STEM course
Source: PLoS One. 2018 Sep 6;13(9):e0202041. doi: 10.1371/journal.pone.0202041 (PMC6126828; doi:10.1371/journal.pone.0202041)
Supplement: S2 File — (PDF) [file pone.0202041.s004.pdf]

Meta-analysis of gen-chem teaching initiatives at R1 universities

Foreword 2
University of Texas at Austin 3
Illinois Urbana-Champaign .... 4 – 5
Columbia .... 6 – 7
Michigan State .... 8 – 9
Florida State .... 10 – 11
California Berkeley 12
Texas Tech .... 13 – 14
North Carolina State .... 15 – 16
South Florida 17
Duke .... 18 – 19
Washington St. Louis .... 20 – 22

Foreword:

Active learning has been identified as the cornerstone of modern pedagogy, but it can not wholly substitute for lack of knowledge and preparation. In this section, we analyze learning initiatives at R1 universities with respect to gen-chem, the sole gateway science course (at Cornell) which typically presupposes at least one year of at least one year of high school same-subject-science instruction. Our reanalysis distinguishes between bridge courses, which we define as semester-long courses placed before the primary gen-chem class, and service courses, which we define as semester-long courses that run parallel to the primary gen-chem class.

Our analysis on R1 university gen-chem interventions relies only on external metrics, such as course grades, course scores, and exam scores. Student attitudes and self-perceptions are not considered due to variability of metrics between studies. Where appropriate, progressive improvement scores and/or two-tailed *p* values were calculated, assuming unpaired samples with unequal variances. A summary of the results is reported in Table S1.

Included in our reevaluations are the Treisman-based calculus programs at Texas Austin and Illinois Urbana-Champaign, where impressive improvements in student final grades were observed. The only gen-chem service course that has achieved results similar to those of earlier programs is the Peer-led Team Learning Program at Washington University at Saint Louis, where a *z*-score improvement of 0.59 was obtained. Bridge courses fared much better: the bridge courses implemented by Texas Tech and Duke were both effective at preparing students for gen-chem.

For each of the nine R1 gen-chem interventions, we also summarize the degree to which the given intervention resembles a Treisman-based intervention. In particular we consider the frequency and length of workshop classes, the collaborative spirit in which the workshops are carried out, the difficulty of the workshop material, and the degree to which the workshop aims at complete student preparation for actual in-class examinations.

In our reanalysis, recent service course that implement active learning (at Florida State, California Berkeley, North Carolina State, South Florida, and Duke), when compared to similar control groups, are found to have p-values of 0.73, 0.94, 0.26, 0.044, and 0.65 respectively. In the sole case where the *p*-value indicates a statistical difference (the South Florida score of 0.044) the external exam score improvement was a somewhat modest 0.13 increase on *z*-score.

These results point both to the strengths and current limitations of active learning with respect to gen-chem, one of the primary gateway STEM courses at all R1 universities. The excellent outcome in gen-chem bridge courses, compared to the weaker outcomes in the service courses suggest that more complete programs may help poorer prepared students more successfully resolve initial information or cognitive-skill gaps. A greater focus on the exact material used in interventions may help allow a better understanding of differences in intervention outcomes.

18. Moreno SE, Muller C, Asera R, Wyatt L, Epperson J (1999) Supporting Minority Mathematics Achievement: The Emerging Scholars Program at the University of Texas at Austin. *J. Women Minor. Sci. Eng.* 5:53–66.

This study conducted at the University of Texas at Austin compares the freshman calculus performances of students in the Emerging Scholars Program (ESP) to that of non-ESP students. This study states that ESP students are more successful than non-ESP students. To confirm this statement, we use data from Table 1 of this paper:

	**ESP**	**Non-ESP**
Mean calculus grade	3.193	2.137
Standard deviation	1.015	1.402
Total students	445	1120

The data from the above table can be used to calculate a *t*-score and a *p*-value to quantify the difference in performance of the students between the two methods. The formula to find the *t*-score is

$$t_{score} = \frac{\bar{x}_1 - \bar{x}_2}{\sqrt{\frac{{S_1}^2}{N_1} + \frac{{S_2}^2}{N_2}}}$$

where $\bar{x}_1$ and $\bar{x}_2$ are the mean grades, S_1 and S_2 are the standard deviations, and N_1 and N_2 are the number of students. The calculated *t*-score is 16.5524 and the degrees of freedom can be calculated by the Welch–Satterthwaite equation:

$$\nu \approx \frac{\left(\frac{s_1^2}{N_1} + \frac{s_2^2}{N_2}\right)^2}{\frac{s_1^4}{N_1^2(N_1 - 1)} + \frac{s_2^4}{N_2^2(N_2 - 1)}}$$

yielding a value of 1117. These values correspond to a *p*-value of 3.32×10^{-55}. Since $p < 0.05$, there is a significant difference between the grades of the ESP and Non-ESP students.

To calculate the improvement of ESP students relative to the non-ESP students, the class standard deviation of 1.388 was used (from Table 1 of the article) and the following calculation was made:

$$\frac{3.193 - 2.137}{1.388} = +0.76$$

This calculation shows that ESP students performed on average 0.76 standard deviations higher than non-ESP students, which confirms the analysis from this paper.

17. Murphy TJ, Stafford KL, McCreary P (1998) Subsequent Course and Degree Paths of Students in a Treisman-style Workshop Calculus Program. *J. Women Minor. Sci. Eng*. 4:381–396.

The paper describes the work at University of Illinois at Urbana-Champaign to answer critical questions pertaining to academic prosperity and inequality. This study targeted the under-represented minority students to improve their grades in calculus. The paper measures success by comparing the mean grades of the Merit Workshop and the Non-Workshop. In our analysis, we compare the difference in the performance of students in the Merit Workshop and the non-workshop for both semesters and calculate average progressive improvement.

Statistical analysis was conducted on this data for progressive improvement of the workshop compared to the class. Using the given mean of the Merit workshop (2.79) and the non-workshop groups (2.42), the standard deviation of the entire class' first semester grades was calculated:

$$\sigma = \sqrt{\frac{\Sigma(x-\mu)^2}{N}} = 1.07$$

The improvement was calculated using the difference between the merit workshop and non-workshop divided by the standard deviation.

Progressive improvement: $\frac{2.79-2.40}{1.07} = +0.36$

We also conducted analysis on Figure 3 in their paper to calculate progressive improvement of the second semester of calculus.

Figure 3 revised

**Grade**	**# of students in Merit Workshop**	**# of students in non-workshop**
A (4)	0.40 x 95 = 38	0.19 x 248 = 47
B (3)	0.36 x 95 = 34	0.31 x 248 = 76
C (2)	0.17 x 95 = 16	0.32 x 248 = 79
D (1)	0.06 x 95 = 6	0.10 x 248 = 25
F (0)	0.01 x 95 = 1	0.03 x 248 = 7
**Average GPA**	3.07	2.56

The students in the "others" category were omitted because the data from the study of this paper also omitted students who withdrew.

Standard deviation of entire class GPA (second semester): 1.03

Progressive Improvement: $\frac{(3.07-2.56)}{2} = +0.50$

Average improvement of 1st and 2nd semester calculus: $\frac{0.36+0.50}{2} = +0.43$

The Merit Workshop showed an improvement of +0.43 (which translate to almost half a letter grade increase).

Data from Figure 2 (first semester calculus) were additionally analyzed to determine the impact of the supplemental workshop on semester grades for the initial semester.

Figure 2 revised

Grade	# of students in Merit Workshop	# of students in non-workshop
A (4)	0.27 x 98 = 26	0.16 x 94 = 15
B (3)	0.36 x 98 = 35	0.33 x 94 = 31
C (2)	0.28 x 98 = 27	0.33 x 94 = 31
D (1)	0.09 x 98 = 9	0.12 x 94 = 11
F (0)	0.01 x 98 = 1	0.06 x 94 = 6
**Average GPA**	2.78	2.40
**Standard deviation**	0.98	1.09

The students in the "others" category were omitted because our study omitted students who withdrew.

The impact of the workshop on the first semester of calculus was determined by calculating the *t*-score and *p*-value. The formula to find the *t*-score is

$$t_{score} = \frac{\bar{x}_1 - \bar{x}_2}{\sqrt{\frac{{S_1}^2}{N_1} + \frac{{S_2}^2}{N_2}}}$$

where $\bar{x}_1$ and $\bar{x}_2$ are the average GPAs, S_1 and S_2 are the standard deviations of the GPAs, and N_1 and N_2 are the number of students. The degrees of freedom for unequal variances can be approximated by the Welch–Satterthwaite equation:

$$\nu \approx \frac{\left(\frac{s_1^2}{N_1} + \frac{s_2^2}{N_2}\right)^2}{\frac{s_1^4}{N_1^2(N_1 - 1)} + \frac{s_2^4}{N_2^2(N_2 - 1)}}$$

The *t*-score is calculated at 2.537 and the degree of freedom is 185. These data correspond to a *p*-value of 0.0120. Since $p < 0.05$ and the program has exhibited strong progressive improvement, we confirm that the program at University of Illinois at Urbana-Champaign led to statistically significant results.

23. Pickering M (1975) Helping the High Risk Freshman Chemist. *J. Chem. Educ.* 52:513–514.

The Columbia supplemental course targeted around 60 students with a Math SAT score of 610 or lower. The program was lecture-based with occasional problem solving sessions. Lectures were focused on problem solving techniques. Brief homework assignments were also required for each meeting.

According to their metrics, the authors state that their supplementary course "did not result in drastic changes in performance." However, they also report that "the difference in mean [course] grade is 0.29 on a four point scale…the *t* value of 2.54 tells us that the grade improvement is significant." Since we measure success based on improvement in the course, in class-exams performance, or final grade performance, we focused on the latter statement by the authors.

Our verification of their statistics is included below. The course was contrasted with a historical control from 1972 of students with math SAT scores lower than 610—when the class did not exist. A revised version of their grade distribution is included as Table 1.

Table 1. Grade Distribution– Revised

**Grade in Lecture Course (GPA)**	**Passed Supplementary Course**	**Historical Control**
A (4)	8.33%	0.00%
B (3)	34.52	32.39
C (2)	42.86	43.66
D (1)	14.29	19.72
F (0)	0.00	4.22
**Average GPA**	2.369	2.042
**Standard deviation**	0.835	0.837

*New percentages were calculated for each grade category as a proportion of total students who received a letter grade (84% and 71% for the supplementary course and historical control, respectively). This is shown in Sample Calculation 1
*Average GPA calculated according to Sample Calculation 2

Sample Calculation 1. Grade Distribution Recalculation

$$New\ Percentage = \frac{Old\ Percentage}{Total\ Included} \times 100\% = \frac{7\%}{84\%} \times 100\% = 8.33\%$$

Sample Calculation 2. Average GPA Calculation

$$Average\ GPA = 4(0.0833) + 3(0.3452) + 2(0.4286) + 1(0.1429) = 2.3689$$

Since the paper indicated that approximately 60 students were included in supplemental instruction but approximately five students had dropped out, n = 55 and n = 60 were used for students with and without supplemental instruction, respectively. Using data from Table 1 above and these sample sizes, a Student's t-test was run on the two groups via the sum of squares method, with each grade category being weighed by the number of students in that category (percent times n). The resulting p-value indicated a significant difference in average GPA between the two groups. Looking at the difference between these groups, these calculations suggest a GPA increase of +0.33 points in lecture course grade between students in supplemental instruction versus historical control. Since our number is greater than theirs, we accept their value of +0.29 GPA points.

The t-score can be calculated using an unpaired t-test:

$$t_{score} = \frac{\bar{x}_1 - \bar{x}_2}{\sqrt{\frac{{S_1}^2}{N_1} + \frac{{S_2}^2}{N_2}}}$$

where $\bar{x}_1$ and $\bar{x}_2$ are the mean GPAs, S_1 and S_2 are the standard deviations of the course GPAs, and N_1 and N_2 are the number of students. The degrees of freedom can be calculated with the Welch–Satterthwaite equation.

$$\nu \approx \frac{\left(\frac{s_1^2}{N_1} + \frac{s_2^2}{N_2}\right)^2}{\frac{s_1^4}{N_1^2(N_1 - 1)} + \frac{s_2^4}{N_2^2(N_2 - 1)}}$$

The *t*-score is 2.095, degree of freedom of 112, and a *p* value of 0.0384.

Thus, by our metric, the program at Columbia was successful in helping students improve at general chemistry because it significantly increased the students' final grade performance.

This intervention predates Treisman's published work. It largely consisted of lectures on problem recognition and algorithmic methods of problem solving. The number of times the lectures met was not reported but the course was a two-credit course, a significant work load may have been expected. Lectures were occasionally replaced with a problem session where students worked through problems on the blackboard. Rather than having students work in groups, homework assignments of about 30 to 60 minutes were required prior to each meeting. No sample problems were provided in the published paper although the authors mention that, in the future, course problems will be drawn from Sienko's *Chemistry Problems*. A review of this book reveals classic problems which test the mastery of a given subject.

24. Nocera DC, Harrison JF, Reed DA, Roberts CH (1996) Enhanced Performance in Chemistry by Minorities at the University Level. *J. Chem. Educ.* 73:1131–1137.

The Michigan State University Tutorial Assistance in Chemistry (TAC) program in concert with the Drew Program targets under-represented groups in science. Data spanning from 1990-1994 of average grade point difference between Drew-TAC students and entire introductory chemistry class were used to measure the success of this program.

The paper states that "TAC sessions offer personalized instruction with the goal of challenging students with the material covered in lecture by providing additional background and exploring topics with greater scope. In many ways, TAC resembles the successful teaching models developed by Shabazz and Triesman in mathematics instruction of minority students." The paper notes the significant measure of success in that "twenty-nine African-American students have graduated since the inception of the program, with B.S. degrees in chemistry, biochemistry, or chemical engineering." The goal of our reanalysis is to determine a *p*-value for the students' performance in general chemistry relative to the entire chemistry class using either their course grades or their exam scores.

Using Figure 6 in their paper, the average grade point differentials were estimated and tabulated below.

Year	Average grade point differential
1990	0.22
1991	0.26
1992	0.30
1993	0.30
1994	0.43
**Average**	0.30

The average grade differential calculated was approximately +0.30. The standard deviation of the five data points alone suggests statistical significance, but without full class numbers, or full class standard deviations, we are not able to run a Student *t*-test.

As an additional metric, we could however assess the performance of students in introductory chemistry during the first year of implementation (1990). Data from Figure 5 were carefully measured and quantified.

Course grade	Drew-TAC students (%)	All students (%)
0	0.000	9.067
1	4.742	6.240
1.5	14.278	9.547
2	14.278	12.907
2.5	14.278	18.507
3	28.609	20.960
3.5	23.815	17.760
4	0.000	5.013
**Average**	2.596	2.377
**St Dev**	0.784	1.079
**Number of students**	21	1907

The number of total students were not reported in the article and assumed to be the total enrolled students in CEM 141 based on the description of the course. Historical records of enrollment were acquired through Michigan State's Office of Planning and Budgets from 1994 to 1998. An average of 1907 ± 117 students enrolled in CEM 141 each year over this span of time.

Based on these data, the *t*-score can be calculated by the following:

$$t_{score} = \frac{\bar{x}_1 - \bar{x}_2}{\sqrt{\frac{S_1{}^2}{N_1} + \frac{S_2{}^2}{N_2}}}$$

where $\bar{x}_1$ and $\bar{x}_2$ are the average grades, S_1 and S_2 are the standard deviations of the course grades, and N_1 and N_2 are the number of students. The degrees of freedom for unequal variances can be approximated by the Welch–Satterthwaite equation.

$$\nu \approx \frac{\left(\frac{s_1^2}{N_1} + \frac{s_2^2}{N_2}\right)^2}{\frac{s_1^4}{N_1^2(N_1 - 1)} + \frac{s_2^4}{N_2^2(N_2 - 1)}}$$

The *t*-score is 1.2669, with a degree of freedom of 20. The *p* value of 0.2197 shows that the Drew-TAC program did not significantly affect the course grade for the treatment group in the first year of implementation alone. However, if we assume similar total course enrollments and year-to-year standard deviations, both for the treatment and control groups, then a derived *p*-value below 0.05 is found for the full five years of data.

According to the authors, the TAC program involved intensive study/problem solving/"drill sessions" of 8 - 12 Drew students guided by a "Merit-level" graduate teaching assistant. These sessions provided additional background information on lecture material and explored topics in greater depth than in lecture and are seen as similar to Treisman workshops. The authors did not mention the time commitment of the course or provide examples of problem sets used. The MSU workshops have a research lab component, which implies a significant level of cognitive work. The intervention pedates the use of flipped classes, and class flipping was not an issue.

25. Dougherty RC, et al. (1995) Cooperative learning and enhanced communication: Effects on student performance, retention, and attitudes in general chemistry. *J. Chem. Educ.* 72:793–797.

In this study at Florida State, methods of cooperative learning were used in teaching general chemistry. The study divided the introductory chemistry class into three groups: a Control group, an Unstructured-Cooperative learning group, and a Structured-Cooperative learning group. The Control group was placed in weekly non-cooperative recitation sections, during which a variety of problems chosen by the recitation leaders and course instructors were solved. In the Unstructured-Cooperative learning group, cooperative learning methods were applied during the recitations: students worked in groups of three or four solving challenging problems posed to them by the recitation leaders and instructors. Finally in Structured-Cooperative learning, in addition to the recitation sections, students worked in structured groups, outside of recitation class, in which they cooperatively solved homework problems as well as other group assignments.

The paper states "it is possible to improve significantly the success rate in university chemistry by the introduction of a limited set of low-cost interventions. Improvement happened when the instructor with the lowest performance in instructional evaluation introduced the interventions. The pedagogical changes used in this study were cooperative learning and enhanced communication." The paper measures its success based on retention and on students performing at a C-grade level or better.

In our reanalysis, we measure improvement in course outcome, solely from course grade or exam outcomes generated by exams external to the service course in question. In the case of Florida State, these external results are the common questions solved by all three groups as opposed to exam results on questions given to the cooperative learning groups alone. These common exam problems were all of multiple-choice type and were all modeled after ACS exam questions. Each of the four exams had seven common questions, while the final exam had 18. The data on these exam questions was extracted from Figure 3 of the paper and are presented below.

**Exam**	**S-Coop (average # correct on exam)**	**U-Coop (average # correct on exam)**	**Control (average # correct on exam)**
1	52.943% × 7 = 3.706	60.535% × 7 = 4.237	48.843% × 7 = 3.419
2	44.187% × 7 = 3.093	42.415% × 7 = 2.969	34.620% × 7 = 2.423
3	52.184% × 7 = 3.653	60.636% × 7 = 4.245	59.573% × 7 = 4.170
4	41.605% × 7 = 2.912	52.690% × 7 = 3.688	56.638% × 7 = 3.965
Final	58.055% × 18 = 10.450	60.080% × 18 = 10.814	54.563% × 18 = 9.821
Number of students	240	180	180
**Average total # of correct questions**	23.814	25.953	23.798

Structured-Cooperative and Unstructured-Cooperative learning program results were averaged to represent the program as a whole. The difference between performances between this treatment group and the control was then calculated for each exam.

Exam	Average (S-Coop + U-Coop)	Control	Difference (average - control)
1	3.972	3.419	0.553
2	3.031	2.423	0.608
3	3.949	4.170	-0.221
4	3.300	3.965	-0.665
Final	10.632	9.821	0.811

Mean difference: $\frac{(0.553+0.608+-0.221+-0.665+0.811)}{5} = 0.2171$

Standard deviation of difference: $\sigma = \sqrt{\frac{\Sigma(x-\mu)^2}{n-1}} = 0.6301$

z-score: $z = \frac{x-\mu}{\sigma} = \frac{0.2171}{0.6301} = 0.3445$

The resulting *z*-score from the difference mean and difference standard deviation was 0.3450, which corresponds to a two-tailed *p*-value of 0.73 at a significance level of 0.05. Therefore, there was no significant difference between the treatment and control groups.

A similar analysis can be conducted on the data from only the Unstructured-Cooperative learning program compared to the Control group (this cooperative learning program, based on the table on the preceding page, has the greatest chance at posting a *p*-value indicating significant difference). Applying an identical treatment to just these 180 unstructured cooperative students and comparing these students to the control group leads to a mean difference of 0.431, a standard deviation of difference 0.526, and a *z*-score of 0.819. This corresponds to a two-tailed *p*-value of 0.41 at a significance level of 0.05. Again, there was no significant difference between the Unstructured and Control groups.

By our metric, the program at Florida State failed to help students improve at general chemistry. The reported paper, using more internal results (such as exam questions written for the cooperative learning students alone) came to a different conclusion.

The program contains aspects of a Treisman program: it focused on cooperative learning and enhanced communication. In the U-Coop section, this was defined by increased cooperative interaction during regular recitations and additional paper mail. In the S -Coop section, this was defined by formal study groups; out-of-class group meetings; graded group homework, quizzes, and chemistry questions via email; and additional emails. The authors did not discuss in great detail the rigor of the homework, quizzes, or additional questions via email, though the authors do mention that in workshop, questions combining two or more concepts were introduced. This lack of emphasis on problem rigor, unlike as in the original Treisman work, suggests a lesser concern in this aspect of the intervention.

26. Gutwill-Wise JP (2001) The Impact of Active and Context-based Learning in Introductory Chemistry Courses: An Early Evaluation of the Modular Approach. *J. Chem. Educ.* 78:684–690.

This study investigates the implementation of ChemConnections modular materials in an introductory chemistry course, which took place at Grinnell College, a small liberal arts college in Iowa, and at the University of California at Berkeley. Table S1 compares solely R1 universities: only results from UC Berkeley are therefore applicable. Furthermore, while researchers in this paper investigated whether students in modular courses of chemistry were as adept at standardized chemistry problems, whether students in modular courses had positive attitudes toward the modules, and whether students in modular courses were better than non-modular students at understanding chemistry, Table S1 reports solely on performance on actual general chemistry exams and final course grades.

Within these contexts, the authors of the paper report that the ChemConnections modular materials led to no significant difference on in-class exam scores with a corresponding *p*-value of 0.94.

We confirm this *p* value using the following data directly extracted from the paper:

**Modular**	**Non-Modular**
$\bar{x}_1$ = 297.54	$\bar{x}_2$ = 297.91
SE_1 = 3.57	SE_2 = 3.20
N_1 = 338	N_2 = 255

The formula to find the *t*-score is:

$$t\text{-score} = \frac{\bar{x}_1 - \bar{x}_2}{\sqrt{\frac{{S_1}^2}{N_1} + \frac{{S_2}^2}{N_2}}},$$

where $\bar{x}_1$ and $\bar{x}_2$ are the mean total scores of in-class exams, S_1 and S_2 are the standard deviations of the in-class exams, and N_1 and N_2 are the number of students. The calculated *t*-score is 0.0772. The degrees of freedom for unequal variances can be calculated by the Welch–Satterthwaite equation:

$$\nu \approx \frac{\left(\frac{S_1^2}{N_1} + \frac{S_2^2}{N_2}\right)^2}{\frac{S_1^4}{N_1^2(N_1 - 1)} + \frac{S_2^4}{N_2^2(N_2 - 1)}}$$

giving a value of 590. These correspond to a *p*-value of 0.938. Since $p > 0.05$, there is no significant difference between the modular group and the non-modular group by in-class exam scores, as stated by the authors.

The reported intervention at Berkeley was one of several different interventions supported by NSF in the 1990s. Other NSF interventions studied Treisman-like workshops and more interactive classes. The Berkeley intervention, by contrast, studied the effect of a modular approach, where students considered several longer modules which framed the issues of more fundamental concepts: for example, a study of airbags was used to develop an understanding of ideal gases and water treatment was considered as a practical problem by which an understanding of acid-base equilibria could be understood. The Berkeley intervention therefore does not measure the effects of a Treisman workshop intervention, but rather emphasizes the role of practical applications in cognitive understanding.

27. Bentley AB, Gellene GI (2005) A Six-Year Study of the Effects of a Remedial Course in the Chemistry Curriculum. *J. Chem. Educ.* 82:125–130.

This study conducted at Texas Tech uses a placement test to identify students who are prepared or unprepared for the general chemistry course. Those who are not prepared are recommended to take a remedial course. From this, three groups are created: those who are prepared for the chemistry course and do not take the remedial course (P), those who take the remedial course and are unprepared (RU), and those who are un-remediated and unprepared (UU). This paper states that "outside of the SAT mathematics score ranges just noted, the various mean score differences were found to be not statistically significant at the 90% confidence level and all differences that were significant at that level failed to be significant at the 95% confidence level".

We have reanalyzed the following data from values extrapolated from Figure 2:

**Math SAT range**	**P – UU (mean score % difference)**	**RU – UU (mean score % difference)**	**P – RU (mean score % difference)**
460 – 500	—	5.708	—
510 – 550	7.413	4.805	—
560 – 600	6.263	3.737	2.505
610 – 650	6.530	—	5.421
660 – 700	6.222	—	4.394
**Mean**	6.735	4.750	4.107
**Standard Deviation**	0.554	0.987	1.479
**Reported Math SAT range groupings**	4	3	3

Since the data are values for mean differences between their respective groups, a one-sample *t*-test compared to zero was used to calculate a *p*-value to determine if the differences were significant. The results to the one sample *t*-tests are below:

**Groups**	***p*-value**
P – UU	0.0002
RU – UU	0.0141
P – RU	0.0406

The significant differences in performance between groups are described below as changes in the final letter grades. Bentley et al. noted that these differences were only statistically significant in a fairly narrow SAT range.

	**P – UU**	**RU – UU**	**P – RU**
Math SAT score	510 – 700	460 – 600	560 – 700
Average letter grade difference	+0.5 to +0.75	+0.25 to +0.5	-0.25 to -0.5

Based on all this information, it can be concluded that there is a significant difference between all three groups, with the UU group scoring lowest, followed by the RU group, and the P group scoring the highest. This shows that the remedial program was effective in improving the scores of underprepared

students. However, when compared to the P group, RU students still scored significantly lower, suggesting that while the remedial program improved the performance of the underprepared students, it was not enough to bring them up to the level of prepared students.

The Texas Tech intervention was a semester long bridge course, required of all students who did not achieve a 50% score on the chemistry placement examination. The authors term the course remedial. Although not explicitly stated in the text, the sense of the intervention course conveyed is of a standard one-semester lecture-based general chemistry course (without laboratory component). The textbook used in this bridge course was Zumdahl's *Basic Chemistry*. By contrast the textbook used in the Texas Tech main general chemistry sequence at the time were the more cognitively advanced Silberberg *Chemistry: The Molecular Nature of Matter and Change* and/or McMurray and Fay *Chemistry*.

It is inferred that the bridge course incorporated neither cooperative learning workshops nor a flipped class. The choice of textbooks supports the notion that less cognitively advanced work was done in the bridge course. Neither the format nor the rigorous content of a Treisman-like program was adopted.

28. Oliver-Hoyo MT, Allen D, Hunt WF, Hutson J, Pitts A (2004) Effects of an Active Learning Environment: Teaching Innovations at a Research 1 Institution. *J. Chem. Educ.* 81:441–448.

This North Carolina State University study explores a new approach to teaching general chemistry called "concept Advancement through chemistry Lab-Lecture", or $cAcL_2$. The program incorporates active learning with classroom management/design skills and collaborative work in groups of nine students. Hand-on activities, demonstrations, and problem sets were designed to cover similar topics in the conventional lecture but in a more interactive format.

The authors implement statistical tests to assess the effectiveness of $cAcL_2$. Use of a three-way ANOVA model allowed them to determine sources of variation, including whether the students' major or class section had an affect on exam performance. Of the four exam score means, they find only exams 2 and 4 show a statistically significant positive improvement for the $cAcL_2$ students. The authors note that student scores in both exams 1 and 3 show no significant difference and ascribe the poor performance of exam 1 to the students being unaccustomed to the format of the $cAcL_2$ classes. Yet, overall, the authors claim that students under the $cAcL_2$ method performed significantly better than students under the conventional method.

However, the performance differences between service-course students and non-service-course students need to be measured by all available external exams. Therefore, all results should be calculated and interpreted as a whole. Data from Table 2 of the paper were used to create the following table:

**Class**	**Conventional Average (SD)**	**$cAcL_2$ Average (SD)**
Exam 1	63.4 (19.8)	57.6 (22.5)
Exam 2	65.4 (13.9)	72.0 (12.8)
Exam 3	58.5 (18.2)	55.6 (19.1)
Exam 4	57.0 (18.8)	66.1 (18.2)
**Sum of Exam Means**	244.3	251.3
**Combined Exam SD**	35.64	36.96
**Number of Students**	48	119

Combined exam means were calculated by summing up the means of the four exams. Combined exam standard deviations were calculated by square rooting the sum of the squares of the four exam standard deviations. The number of students in each class is reported in the paper.

The data from the above table can be used to calculate a *t*-score and a *p*-value to quantify the difference between the two methods. The formula to find the *t*-score is

$$t\text{-score} = \frac{\bar{x}_1 - \bar{x}_2}{\sqrt{\frac{S_1{}^2}{N_1} + \frac{S_2{}^2}{N_2}}},$$

where $\bar{x}_1$ and $\bar{x}_2$ are the combined exam means, S_1 and S_2 are the combined exam standard deviations, and N_1 and N_2 are the number of students. The calculated *t*-score is 1.136. Assuming unequal variances, the degrees of freedom are calculated from the Welch–Satterthwaite equation

$$\nu \approx \frac{\left(\frac{s_1^2}{N_1} + \frac{s_2^2}{N_2}\right)^2}{\frac{s_1^4}{N_1^2(N_1 - 1)} + \frac{s_2^4}{N_2^2(N_2 - 1)}}$$

giving a value of 89. These values correspond to a *p*-value of 0.2588. Since $p > 0.05$, there is no significant difference between the conventional and $cAcL_2$ methods for overall performance.

The NCSU $cAcL_2$intervention is one based on interactive learning. Students in the intervention work in an open space collaborative format, meeting three times a week, at a hundred minutes per session versus students not in the intervention, who met just twice a week for seventy-five minute lectures. $cAcL_2$ students therefore had twice as many contact hours as students not in the intervention.

Higher order cognitive skills were stressed in the $cAcL_2$ classes. Five examples of chemistry problems are provided in the paper (covering a range of general chemistry topics) as examples of the higher-order cognitive skills required. Given the apparent adherence of the NCSU intervention to the principles of Treisman workshop pedagogy, the lack of a significant measurable difference between the conventional and $cAcL_2$ class performance is especially noteworthy. A detailed study comparing pedagogical methods with that of other Treisman-like interventions could prove informative.

29. Lewis SE, Lewis JE (2008) Seeking Effectiveness and Equity in a Large College Chemistry Course: An HLM Investigation of Peer-Led Guided Inquiry. *J. Res. Sci. Teach.* 45:794–811.

This study explores a peer-led cooperative learning method of teaching general chemistry, Peer-Led Guided Inquiry (PLGI) at the University of South Florida. Each week, PLGI students had one of their lectures supplanted with a PLGI session such that total instructional time was comparable to non-PLGI students. These sessions involved working in small groups on guided inquiry activities that were facilitated by peer leaders. Activities aimed to introduce concepts before they were explained in the main lecture course. All PLGI students were expected to complete assignments in preparation for each session and were quizzed regularly on the previous week's information.

The paper states that PLGI "was associated with statistically significant improvement over traditional pedagogy." This claim was based on the externally constructed ACS Exam as well as four internally constructed midterm exams. Specifically, "PLGI was associated with improved performance on the ACS Exam... [and] experience a much less severe drop in performance as the semester progresses." The paper states this effect translates in an increase in the fourth midterm exam score of 4.7 percentage points, approximately 0.25 of a standard deviation.

The authors implement two separate regression models, a hierarchal linear model (HLM) and a time series model, to assess the effectiveness and equity of PLGI. The models controlled for the effect of student SAT scores on achievement, aiming to show that PLGI is able to both improve overall student academic performances and to equate the disparity of achievements between student groups. Their analyses show that enrollment in PLGI resulted in an overall 1.19 pt increase on the ACS exam but did not significantly improve student achievement for low SAT scorers; students who were underprepared were not fully remediated by the program. Thus, the authors conclude that PLGI was effective at improving student performance but was not able to promote equity.

In our reanalysis, we evaluated their results by implementing the same standard methods used for assessing the other programs. Since the in-class exams were written in part by the PLGI instructor, the internal measure may be considered an internal rather than an external metric. Therefore, we based our verification of the effectiveness of their program on the external ACS Exam scores. The following table is created from the ACS Exam score data in Table 1 of the paper:

Group	Mean	Standard Deviation	Number of Students
PLGI	22.8	6.9	287
Non-PLGI	21.9	6.8	1453
All Students	22.0	6.8	1740

Using these values, we ran a Welch's t-test on the PLGI and non-PLGI groups, which resulted in t-score = 2.0241, df = 403, and p = 0.0436. There was an improvement of 0.13 (assuming a population standard deviation of 6.9). This statistically significant difference supports the claim that PLGI improved student performance in general chemistry albeit to a small degree.

Non-PLGI groups had three traditional 50 minute lectures a week; PLGI students had the same number of contact hours each week with two traditional 50 minute lectures and one small group peer-led guided inquiry each week. While this intervention, with its cognitively-based collaborative work sessions follows some components of a Treisman intervention, but with only one hour instead of four hours a week of such workshops, does not match the full amplitude of a Treisman program. Being a PLGI, the workshops would have been dedicated to scientific inquiries rather than adhering to the three points outlined by Treisman in a rigorous workshop program.

8. Hall DM, Curtin-Soydan AJ, Canelas DA (2014) The Science Advancement through Group Engagement Program: Leveling the Playing Field and Increasing Retention in Science. *J. Chem. Educ.* 91:37–47.

Duke's gen-chem service course is part of its SAGE program. The SAGE program, itself, is designed to benefit students who are less experienced in science, measured by their math SAT scores and lack of AP courses in math or science. The placement criteria for SAGE is less than one year of high school chemistry or a math SAT less than or equal to 630. The SAGE intervention occurs over the first two years of classes. The program measures success in terms of improvements on exams and final grades in courses.

During their first semester of college, all SAGE participants enroll in "Introduction to Chemistry," a course designed to prepare students for "General Chemistry 1." Introduction to Chemistry has the hallmarks of a bridge course: less well-prepared students enroll in the bridge course before subsequently joining a greater number of better prepared gen-chem students the following semester. Importantly for this bridge course designation, the bridge course students join the better prepared students in their very first university chemistry course.

Hall, Curtin, and Canelas investigate three groups for General Chemistry 1: SAGE, Control 1, and Control 2. The SAGE group consists of SAGE participants who took Introduction to Chemistry and participated in General Chemistry 1, as well as the SAGE gen-chem service course. Control 1 consists of students who had an SAT score greater than 630 and had at least one year of high school chemistry and therefore were never participants in SAGE and never took the SAGE bridge course (Introduction to Chemistry) or the subsequent SAGE service course. Control 2 includes the students who dropped out of the SAGE program after taking Introduction to Chemistry and did not take the SAGE gen-chem service course.

Hall et al. demonstrate that the SAGE bridge course "Introduction to Chemistry" was successful at preparing students for General Chemistry 1. The effectiveness of this bridge course can be assessed by comparing the course grades of the non-SAGE students to those of the SAGE students.

**SAGE**	**Non-SAGE**
$\bar{x}_1 = 3.3$	$\bar{x}_2 = 3.2$
$S_1 = 0.6$	$S_2 = 0.8$
$N_1 = 29$	$N_2 = 64$

The corresponding *t*-score of 0.6679, degrees of freedom of 70, and *p* value of 0.506 signify that the Introduction to Chemistry course was able to ameliorate performance differences between SAGE students and prepared students.

Interestingly, despite the efficacy of their bridge course, Hall et al. reported no statistical difference engendered by their spring semester SAGE gen-chem service program, which ran concurrent to General Chemistry 1, as attested by a comparison of scores between the SAGE and Control 2 groups.

To verify the reported gen-chem service course efficacy, we reexamined the final General Chemistry 1 course grades of the SAGE group with the Control 2 group based on the data Hall et al. presented in their paper:

SAGE	Control 2
$\bar{x}_1$ = 2.8	$\bar{x}_2$ = 2.9
S_1 = 1.0	S_2 = 0.7
N_1 = 31	N_2 = 33

The data from the above table can be used to calculate a *t*-score and a *p*-value to quantify the difference between the two methods. The *t*-score is

$$t\text{-score} = \frac{\bar{x}_1 - \bar{x}_2}{\sqrt{\frac{{S_1}^2}{N_1} + \frac{{S_2}^2}{N_2}}} \ ,$$

where $\bar{x}_1$ and $\bar{x}_2$ are the mean final course grades, S_1 and S_2 are the final course grade standard deviations, and N_1 and N_2 are the number of students. The degrees of freedom for unequal variances can be approximated with the Welch–Satterthwaite equation.

$$\nu \approx \frac{\left(\frac{s_1^2}{N_1} + \frac{s_2^2}{N_2}\right)^2}{\frac{s_1^4}{N_1^2(N_1 - 1)} + \frac{s_2^4}{N_2^2(N_2 - 1)}}$$

The calculated *t*-score is 0.4607 and the degrees of freedom are 53. These data correspond to a *p*-value of 0.647. Since $p > 0.05$, there is no significant difference between course grades for SAGE and Control 2, as stated by Hall et al.

SAGE is a cooperative learning based intervention, where students meet in small groups with a peer team leader (PLTL). although no actual problems were reported, from the overall description, efforts were made to develop appropriately challenging work. The absence of any reported new posed problems suggest however that changes in problem type were not a primary focus of the intervention.

The two reported gen chem SAGE interventions each had different apparent outcomes: intervention in the the preliminary Introduction to Chemistry class led to statistically measurable improvement while a seemingly similar program in General Chemistry 1, as analyzed above, did not appear to lead to outcome improvement. The former intervention had two PLTL sessions per week while the latter had only one (with PLTL sessions running each at 90 minutes long).

The program adopted several components of the Treisman method: workshops were an integral part of the intervention, and at least in the Introduction to Chemistry course, the workshops were at the Treisman compatible biweekly level. Unlike in the original Treisman method, the workshop problem sets do not appear to be the most important part of the program.

30. Hockings SC, DeAngelis KJ, Frey RF (2008) Peer-Led Team Learning in General Chemistry: Implementation and Evaluation. *J. Chem. Educ.* 85(7):990–996.

The Peer-Led Team Learning (PLTL) was adopted at Washington University in St. Louis as a supplemental course where students self-selected themselves into the course. The paper states "Peer-led team learning introduces students to effective group study by supplementing the lecture with formalized study groups that enforce active learning."

Our verification of the statistics is included below. The final grades of these students were compared to their non-PLTL peers. The average final grade reported for the PLTL students is 2.94 while their non-PLTL peers had an average final grade of 2.74. The paper measures success by comparing the final grade mean between PLTL students and non-PLTL students. There were 450 PLTL students and 675 non-PLTL students with average mean grades of 2.94 and 2.74 respectively. We can compare their performance by looking at the difference:

$$2.94 - 2.74 = 0.2$$

The difference between their averages is +0.2, which amounts to almost a difference in letter grade sign change. This shows that their program succeeded in improving their students' ability in chemistry and had an effective approach.

Although we do not know the standard deviations of the two groups, we can assume that the standard deviation of a grade distribution will be no greater than 1. Using this assumed standard deviation, we can calculate an estimated *t*-score using an unpaired *t*-test:

$$t\text{-score} = \frac{\bar{x}_1 - \bar{x}_2}{\sqrt{\frac{{S_1}^2}{N_1} + \frac{{S_2}^2}{N_2}}}$$

The estimated *t*-score is 3.2863, degree of freedom 962, and a *p*-value of 0.0011. Thus, by our metric, the PLTL program was successful in helping students improve at general chemistry and significantly increased their students' final grade performance. A further report on the Washington University intervention is given in the paper below. Discussion of the Treisman characteristics of the Washington University intervention are therefore given below as well.

7. Shields SP, et al. (2012) A Transition Program for Underprepared Students in General Chemistry: Diagnosis, Implementation, and Evaluation. *J. Chem. Educ*. 89:995–1000.

The Washington University in St. Louis gen-chem service course is based on peer-led team-learning (PLTL). PLTL differentiates itself through beginning coursework in the summer before class, diagnosing underprepared students at the beginning of the year, and providing supplementary material that is tightly intertwined with the general chemistry class. Shields et al. analyzed the effectiveness of PLTL on the population of general chemistry predicted to be in the bottom two quintiles. They used data from fall semester general chemistry classes in 2007, 2008, and 2009. They separate the studied population into four groups, as defined below.

- Group 1: Extended recitation and PLTL
- Group 2: Extended recitation, PLTL, and peer mentoring
- Group 3: Regular recitation, PLTL, and peer mentoring
- Group 4: Regular recitation only

The study compares each group's performance to group 4 through effect size, defined as the marginal mean difference reported between a particular group and group 4. Effect sizes are listed in Table 1.

Table 1: The effect size for groups 1 through 3 compared to group 4.

Group	Effect Size
1 vs 4	0.76
2 vs 4	1.22
3 vs 4	0.92

We computed a *t*-score where all PLTL groups (ie., group 1-3) were placed into a single grouping and compared with group 4 as a control group. Calculations used to determine this *z*-score are seen below.

Table 2: The performance of each group in fall general chemistry classes.*

Group	Final Score Mean	SD on Mean	Number of participants
1	65.76	7.25	68
2	70.25	6.54	96
3	69.61	8.51	189
4	61.62	12.61	73

*Data taken from Table 1 of original article

Weighted mean of groups 1 through 3: 69.04
Standard of deviation of groups 1 through 3: 7.75
t-score (unpaired): 4.8419
df (unequal variances): 83
p value (two tailed, $\alpha = 0.05$) = 5.879×10^{-6}
z-score (improvement) = +0.59

To calculate the mean of groups 1 through 3:

$$\mu_{1,2,3} = \frac{n_1\mu_1 + n_2\mu_2 + n_3\mu_3}{n_1 + n_2 + n_3}$$

where μ are the final score means and n are the number of students per group.

To calculate the standard deviation of groups 1 through 3:

$$S_i = \sqrt{\frac{\sum_j (x_{i,j} - \mu_i)^2}{N_i - 1}}$$

$$\Rightarrow \sum_j (x_{i,j} - \mu_i)^2 = S_i^2 * (N_i - 1)$$

$$S_{1,2,3} = \sqrt{\sum_j \frac{(x_{1j} - \mu_{1,2,3})^2}{n_1 + n_2 + n_3} + \sum_j \frac{(x_{2j} - \mu_{1,2,3})^2}{n_1 + n_2 + n_3} + \sum_j \frac{(x_{3j} - \mu_{1,2,3})^2}{n_1 + n_2 + n_3}}$$

To calculate the *z*-score of PLTL groups compared to group 4:

$$\frac{\mu_{1,2,3} - \mu_4}{\sigma} = \frac{69.04 - 61.62}{12.61} = +0.59$$

As the population standard deviation is unknown, we overestimate σ with the largest reported standard deviation of the final scores.

The Washington University intervention, by statistical measure, is the most successful of the nine R1 gen-chem programs analyzed in this section. At first glance, however, the intervention seems no different from interventions reported elsewhere. Students met once a week in small peer-led groups, in this program in 2-hour workshops. In describing the workshops, Shields et al. write, " In each (2-hr) session, the group discusses concepts covered that week in class, works collaboratively on homework, PLTL problems, or any question students may have, and discusses techniques for transitioning to a university-level curriculum." From the description it is difficult to measure the cogntive demands of the workshop program.
