# Supplementary material for: Patching a leak in an R1 university gateway STEM course
Source: PLoS One. 2018 Sep 6;13(9):e0202041. doi: 10.1371/journal.pone.0202041 (PMC6126828; doi:10.1371/journal.pone.0202041)
Supplement: S1 Table — (PDF) [file pone.0202041.s005.pdf]

Table S1: Summary of teaching initiatives at R1 universities: Emerging Scholars programs vs. gen-chem programs.

University	Program Name (type)	Subject	Population served	Years reported	Control group	*p*-value and/or improvement [1]
Texas Austin[18]	Emerging Scholars	calculus	predominantly URM (66%)	1988-1996	remaining class	$p = 3.32 \times 10^{-55}$; +0.76 (final grade)
Illinois Urbana-Champaign[17]	Merit Workshop (Emerging Scholars)	calculus	predominantly URM (> 70%)	1990-1992	remaining class	$p = 0.012$; +0.43 (final grade)
Columbia[23]	supplemental course	gen-chem	< 610 math SAT	1972-1974	<610 math SAT (historical control)	$p = 0.038$; +0.29 (final grade)
Michigan State[24]	Drew-TAC (grad TA-led workshops)	gen-chem	URM	1990-1994	entire class	*p* value assumed < 0.05 [2] $p = 0.22$ (for 1990 alone); +0.30 (final grade)
Florida State[25]	Cooperative learning	gen-chem	all	1995	remaining class	$p = 0.73$ (program-wide exam problems)
California Berkeley[26]	Context-based learning	gen-chem	all	1998	entire class	$p = 0.94$ (in-class exam)
Texas Tech[27]	extra semester bridge course	gen-chem	below bar pretest & SAT math 560-700	1996-2001	below bar pretest & SAT math 560-700 (historical control)	$p = 0.014$; [3] +0.25 to +0.5 (letter grade)
North Carolina State[28]	$cAcL_2$ (active learning)	gen-chem	all	2001	remaining class	$p = 0.26$ (in-class exams)
South Florida[29]	PLGI (peer-led guided inquiry)	gen-chem	all	2002-2005	remaining class	$p = 0.044$ (ACS final exam) +0.13 (ACS exam score)
Duke[8]	SAGE (extra semester bridge class)	gen-chem	little prev. chem. & <630 math SAT	2010	remaining class	$p = 0.506$ (final grade) High *p*-value suggests cohort equalization [4]
Duke[8]	SAGE (peer-led parallel service class)	gen-chem	little prev. chem. & <630 math SAT	2010	remaining little prev. chem & <630 math SAT	$p = 0.65$ (final grade) High *p*-value suggests limited educational gain
Washington St. Louis[30]	Peer-led team learning	gen-chem	all	2003-2004	remaining class	$p = 0.0011$; +0.20 (final grade)
Washington St. Louis[7]	Peer-led team learning	gen-chem	pretest lowest two quintiles	2007-2009	remaining lowest two quintiles	$p = 5.88 \times 10^{-6}$; +0.59 (final score)
this study	flipped + peer-led	gen-chem	non-priority	2015-2016	remaining non-priority	$p = 1.69 \times 10^{-12}$ +0.61 (*z*-score course total)
this study	flipped + peer-led	gen-chem	priority	2015-2016	remaining priority	$p = 6.18 \times 10^{-9}$ +0.62 (*z*-score course total)
this study	flipped + peer-led	gen-chem	priority	2015-2016	non-service class	$p = 0.57$ +0.05 (*z*-score course total) [4]

[1] Improvement metric listed if $p < 0.05$.

[2] Insufficient data to allow *p* value determination for the five reported years. We calculate a higher *p* value using the final course grade from the initial year of the study (1990).

[3] Texas Tech results can be viewed in a positive light, but original TTU authors conclude that the "program as implemented at TTU is providing little or no significant academic benefit." [27,42]

[4] Statistical differences between confirmed groups removed. Initially less prepared students perform similarly to remaining or non-service-class students.
